# *Waptia fieldensis* Walcott, a mandibulate arthropod from the middle Cambrian Burgess Shale

**DOI:** 10.1098/rsos.172206

**Published:** 2018-06-20

**Authors:** Jean Vannier, Cédric Aria, Rod S. Taylor, Jean-Bernard Caron

**Affiliations:** 1 Université de Lyon, Université Lyon 1, ENS de Lyon, CNRS, UMR 5276 LGL-TPE, Bâtiment Géode, 2, rue Raphaël Dubois, Villeurbanne 69622, France; 2 State Key Laboratory of Palaeobiology and Stratigraphy, Nanjing Institute of Geology and Palaeontology, Chinese Academy of Sciences, 39, East Beijing Road, Nanjing 210008, People's Republic of China; 3 Manuels River Hibernia Interpretation Centre, 7 Conception Bay South Highway, CBS, Newfoundland, Canada A1W 3A2; 4 Department of Earth Sciences, Memorial University of Newfoundland, St John's, Newfoundland, Canada A1B 3X5; 5 Department of Natural History (Palaeobiology Section), Royal Ontario Museum, 100 Queen's Park, Toronto, Ontario, Canada M5S 2C6; 6 Department of Ecology and Evolutionary Biology, University of Toronto, Toronto, Ontario, Canada M5S 3B2, Toronto, Ontario, Canada M5S 3B2; 7 Department of Earth Sciences, University of Toronto, Toronto, Ontario, Canada M5S 3B1

**Keywords:** Burgess Shale, Cambrian, arthropoda, mandibulata, crustacea, *Waptia*

## Abstract

*Waptia fieldensis* Walcott, 1912 is one of the iconic animals from the middle Cambrian Burgess Shale biota that had lacked a formal description since its discovery at the beginning of the twentieth century. This study, based on over 1800 specimens, finds that *W. fieldensis* shares general characteristics with pancrustaceans, as previous authors had suggested based mostly on its overall aspect. The cephalothorax is covered by a flexible, bivalved carapace and houses a pair of long multisegmented antennules, palp-bearing mandibles, maxillules, and four pairs of appendages with five-segmented endopods—the anterior three pairs with long and robust enditic basipods, the fourth pair with proximal annulations and lamellae. The post-cephalothorax has six pairs of lamellate and fully annulated appendages which appear to be extensively modified basipods rather than exopods. The front part of the body bears a pair of stalked eyes with the first ommatidia preserved in a Burgess Shale arthropod, and a median ‘labral’ complex flanked by lobate projections with possible affinities to hemi-ellipsoid bodies. *Waptia* confirms the mandibulate affinity of hymenocarines, retrieved here as part of an expanded Pancrustacea, thereby providing a novel perspective on the evolutionary history of this hyperdiverse group. We construe that *Waptia* was an active swimming predator of soft prey items, using its anterior appendages for food capture and manipulation, and also potentially for clinging to epibenthic substrates.

## 1. Introduction

Our current understanding of early animal evolution and ecology has seen major improvements in recent years. Such advances were made possible owing in part to spectacular new fossil discoveries from several Cambrian Burgess Shale-type deposits, reappraisals of existing fossil collections, and the use of improved photographic and analytical techniques. In recent years, the reinterpretation of iconic taxa from the Burgess Shale biota such as *Odontogriphus* [1], *Hurdia* [2], *Isoxys* [3], *Nectocaris* [4], *Pikaia* [5], *Ottoia* [6], *Wiwaxia* [7], *Hallucigenia* [8] and *Branchiocaris* [9] has shed new light on the early history of animal phyla and has also revolutionized our vision of ecological diversity and trophic structure in Cambrian marine ecosystems. The integration of fossil and neontological datasets using modern cladistic approaches has helped clarify the wider evolutionary relationships of many previously problematic fossil taxa, although a consensus has yet to emerge as to the phylogenetic position of many clades.

Future progress in this field remains more than ever critically dependent on adding robust fossil evidence. In this context, several iconic fossil forms remain, perhaps surprisingly, still largely unknown despite their potential role in revealing key insights into early animal evolution and the ecological structure of Cambrian communities. This is the case of the shrimp-like arthropod *Waptia fieldensis* [10], which, despite being a familiar member of the Burgess Shale community, remains certainly one of the least studied Cambrian arthropods and as a consequence its phylogenetic relationships have remained largely unexplored. A revision is especially timely given the recent reappraisal of the bivalved arthropod *Branchiocaris* and its close relatives from the Burgess Shale (protocaridids) as early mandibulates [9], implying that many of the species with bivalved carapaces (including *Waptia* as part of the order Hymenocarina, revised here) may likewise shed light on the early radiation of mandible-bearing euarthropods.

*Waptia* first appeared in Charles Walcott's field notes at the beginning of the last century [10,11] but had never been the focus of a comprehensive anatomical and interpretative treatment. Recent studies detailing the discovery of neural tissues [12–15] and brood care [16] based on a limited number of specimens have opened an exciting new field of investigation into the neuroanatomy and reproductive strategies of ancient arthropods.

Based on a much larger dataset, this study challenges previous interpretations concerning *W. fieldensis*. It is based on a thorough investigation of all *W. fieldensis* specimens available from the two major Burgess Shale repositories, around 860 specimens from the Smithsonian Institution (National Museum of Natural History), Washington DC, and around 1000 specimens from the Royal Ontario Museum, Toronto. Digital photography using interference lighting, scanning electron microscopy (SEM) and elemental mapping are used in combination to explore the external and internal anatomy of *W. fieldensis*, in particular its appendages and internal organs, including digestive and putative nervous systems and sensory organs. Based on these new data, we reconsider the possible lifestyle of *W. fieldensis,* in particular its locomotion, sensory perception and possible feeding mechanism, and discuss the role of this arthropod in the Cambrian trophic web. Detailed information on its appendage structure and internal anatomy is also coded into a comprehensive cladistic analysis that includes a large number of other arthropod groups. This cladistic approach aims to resolve the phylogenetic relationships of *W. fieldensis* and its relatives among extinct and extant arthropod groups, testing various hypotheses put forward by previous authors, notably its possible affinities with crustaceans.

## 2. Previous work

*Waptia fieldensis* Walcott, 1912 [10] is one of the first fossils from the Burgess Shale discovered by Charles D. Walcott, appearing as a sketch in his 1909 field book [11]. Walcott further collected hundreds of well-preserved specimens of this arthropod from a 2 m interval of the Burgess Shale subsequently referred to as the ‘Phyllopod Bed’ (part of the Walcott Quarry today; electronic supplementary material, S1). He published the first succinct description of *W. fieldensis* in 1912 [10], which he tentatively assigned to branchiopod crustaceans, calling it ‘one of the most beautiful and graceful of the remarkable crustaceans from the Burgess Shale’. In this brief study, Walcott mentioned that *W. fieldensis* occurred in ‘relative abundance’ [10, p. 182] although only two specimens were figured ([10]; plate 27, figs 4, 5). He regretted that no specimen revealed the arrangement of its anteriormost appendages. Ironically, a third specimen, wrongly attributed to the primitive arthropod *Burgessia bella* [17] and figured in the same original monograph, shows the anterior appendages relatively well ([10]; plate 30, fig. 4).

A revised and slightly improved description using many additional specimens and showing the first reconstructions (ventral and lateral views) of *W. fieldensis* appeared in a posthumous publication almost two decades later ([18]; figs 6 and 7).

Many questions remained unanswered, however, as evidenced by the reconstructions which, for example, still showed unspecified cephalic limbs between the antennules and the posterior appendages. Walcott's specimens were later re-examined by Simonetta [19] and Simonetta & Delle Cave [20] who figured a large number of additional specimens from Walcott's collections but provided no crucial new information on the appendage structure. Hughes (*in* [21]) gave the most recent definition of the body plan of *W. fieldensis* in which the trunk would consist of three different tagmata: four segments with walking appendages, six with gill-like branches (the so-called ‘blade-shaped filaments'), followed by six apodous telescopic segments and with the terminal somite bearing a pair of fan-like flattened lobes. This work—a single published page in a general account on the Burgess Shale fauna, with no detailed illustration of specimens—remained preliminary. Hughes intended to work on a large monograph, which was never published.

If we except the long multisegmented antennules and the prominent eyes that are often well-preserved, the detailed appendage morphology of *W. fieldensis* has thus remained unclear for more than a century, allowing only limited discussion on the possible phylogenetic affinities of this arthropod within [22–24] or outside crustaceans. More recently, Strausfeld [12–15] revisited *W. fieldensis* based on a limited number of specimens, describing an array of putative remains of neural tissues, notably represented by traces of brain ganglia, antennule nerves, nervous swellings within the eye peduncle (optic neuropils) and possible frontal organs (ocelli). Some of these neural structures were put forward to suggest a mandibulate affinity of *Waptia,* supposedly within Pancrustacea or even close to Hexapoda. In addition, other morphological features (e.g. appendages, sensillae) were used by Strausfeld [12,15] to infer various behavioural traits of *W. fieldensis* (e.g. locomotion and chemical attraction to food or mates).

The most recent work on *W. fieldensis* [16] revealed the capacity of this arthropod to brood eggs and embryos under the lateral flaps of its bivalved carapace providing direct evidence for parental care among Cambrian arthropods.

## 3. Material and methods

### 3.1. Origin of the fossil material

The fossil material studied here comes from collections of the National Museum of Natural History, formerly the United States National Museum (USNM), Washington DC, USA and the Royal Ontario Museum (ROM), Toronto, Canada, as well as a limited number of specimens from the Geological Survey of Canada (GSC). *Waptia* specimens are all from the Burgess Shale Formation (Cambrian Series 3, Stage 5; around 508 Ma; see [11,25–29] for geology and stratigraphy) exposed near Field, southwest part of British Columbia, Canada (electronic supplementary material, S1). They were recovered from the following localities and horizons (from older to younger stratigraphic units): (i) Kicking Horse Shale Member (SG locality), (ii) Campsite Cliff Shale Member (Tulip Beds and Trilobite Beds localities), (iii) Greater Phyllopod Bed in the Walcott Quarry (WQ) Shale Member, (iv) Raymond Quarry (RQ) Shale Member and (v) the Odaray Shale Member. The fossil material was collected by Charles D. Walcott himself starting in 1909 [29], Percy Raymond in 1930, the Geological Survey of Canada in 1966 and 1967 and, from 1975 to 2000, by ROM parties led by Desmond Collins, through successive seasons of excavations especially from the Walcott Quarry on Fossil Ridge. Additional material was collected during the 2010 ROM party led by one of us (J.-B.C.) near Odaray Mountain. Charles D. Walcott [10] described the distribution of *Waptia* as ‘limited to a band of dark siliceous shale about 4 feet in thickness forming a part of the Burgess Shale member of the Stephen Formation’ in his quarry [10, p. 181] which corresponds to the layers 5 to 12 of his Phyllopod Bed section. *Waptia fieldensis* appears in layer 10, and ‘of more and less frequent occurrence’ in layer 12 (also known as the ‘Great *Marrella* Layer’) at the base [10, p. 153 and 181] (electronic supplementary material, S1). About 1000 specimens are deposited in the USNM collections. Excavations conducted by the ROM within the Greater Phyllopod Bed have yielded around 800 additional specimens of *W. fieldensis* through a total thickness of about 6 m, the highest numerical abundance being recorded at levels 1.2 m (74), 1.3 m (333), 1.5 m (44) and 2.1 m (97) below the base of WQ (ROM database; electronic supplementary material, S1). *Waptia fieldensis* also occurs in the Raymond Quarry, about 20 m above the base of WQ. From one to six specimens were collected (RQ) nearly every 10 cm through a thickness of about 6.5 m for a total of 70 specimens. Altogether about 1870 USNM and ROMIP (Royal Ontario Museum, Invertebrate Palaeontology) specimens were available for study.

### 3.2. Methods

#### 3.2.1. Fossil material

Microscopic observations and light photography of fossil specimens were made in Lyon, Washington DC and Toronto with stereomicroscopes (Leica MZ125 and M205C stereomicroscopes equipped with Plan 1.0-and Planapo 1.6-lenses, digital camera and Leica LAS 3.7.0 imaging system with multifocus option; Nikon SMZ 1500 stereomicroscope) and digital cameras (D3X-Nikon camera with Nikon Micro-Nikkor 60 mm lens; Canon EOS 5DsR digital SLR with macro lenses). We used interference cross-polarized light techniques to see anatomical features through increased contrast among carbon-rich layers, metamorphic clay phases and shale matrix. Some specimens were immersed in water before being observed under the stereomicroscope or SLR camera and photographed. SEM (with FEI Quanta FEG 250) was performed at the Centre Technologique des Microstructures (CTµ), Université Claude Bernard, Lyon 1 to study the detailed morphology of the fossil and extant species. Images were acquired with secondary electron and backscattered electron detectors at 15 kV and 10 kV and under high vacuum. No sputter coating was used with the fossil material from the Burgess Shale. An environmental scanning electron microscope (FEI Quanta 200 FEG) was used (JBC) at the University of Windsor, Canada to obtain elemental maps of selected specimens. These maps were created using an energy scanning spectroscopy X-ray detector and octane plus silicon drift detector (using Team software v. 4.1).

#### 3.2.2. Biological material

Specimens of *Nebalia bipes* (Crustacea, Leptostraca) and *Crangon crangon* (Crustacea, Decapoda) were purchased from the Roscoff Marine Station (Centre de ressources biologiques marines, Roscoff, Brittany, France) for anatomical comparisons with *W. fieldensis* (e.g. visual and sensory organs, gills). They were fixed and kept in 70% ethanol before being dehydrated by using hexamethyldisilazane or via the critical point method (Leica EM CPD 300 at CTµ, Lyon) prior to examination under the SEM.

#### 3.2.3. Phylogenetic methods

Cladistic analyses based on a Bayesian probabilistic method were performed with MrBayes v. 3.2.6 [30] on two datasets: (i) one of 85 adult panarthropod taxa (fossils and extant) and 219 characters, and modified from [9] (see §6); (ii) another of 97 adult and larval panarthropod taxa (fossils and extant) and the same 219 characters.

In each case, characters were unordered and unweighted, and inapplicable entries were treated as uncertainties. The outgroup was set to be Priapulida and the monophyly of all taxa apart from Priapulida and Nematoda was constrained, with the offset exponential rooting set between 540 and 550 Myr. Parameters were configured according to the Mkv + Γ model [31]. The analysis produced trees during four runs of 20 000 000 generations with four parallel chains, a tree sampled every 1000 generations and burn-in of 20%. A backbone constraint was enforced based on a consensus of recent molecular results [32,33], including a recent phylogenomic reinvestigation of Myriapoda [34]. In addition, the tree was time calibrated for fossils and some key extant taxa (Pycnogonida, Scorpiones, Ostracoda and Odonata; see §6).

A Bayesian approach was preferred to parsimony in part because recent studies have found Bayesian topologies to be more accurate (as in [35,36]). Estimating the likelihood of an evolutionary model was also preferred to parsimony to try and better account for character evolvability—resulting in patterns such as character states being variable in one part of the tree (leading to homoplasies) but constrained as apomorphic in another. Such a dynamic evolutionary model is difficult to conciliate with the rigour of parsimony, especially when further downweighting homoplasies with implied weights [37,38]. Using a topological backbone is a methodological alternative that synthesizes sound and consensual results obtained from various molecular as well as morphological studies focusing on extant taxa, without the need to enter a massive number of question marks in the fossil-inclusive dataset, as would be the case with a traditional total-evidence approach (see also [9,35]). Instead of forcing the software to assume the states of a myriad of unfossilized characters in extinct taxa, these taxa are instead placed on the known topology using only the available evidence.

### 3.3. Statistical analyses

Statistical tests were performed with R (R Core Team 2017), with the help of added functionality from the package sm. Specifically, sm.density.compare was used to plot Kernel density curves and generate a bootstrapped reference band while investigating sexual dimorphism.

### 3.4. Institutional abbreviations

GSC, Geological Survey of Canada, Ottawa; ROMIP, Royal Ontario Museum Invertebrate Palaeontology Collections, Toronto, Ontario, Canada; USNM, National Museum of Natural History, Smithsonian Institution, Washington DC, USA.

## 4. Preservation

**Figure 1.**
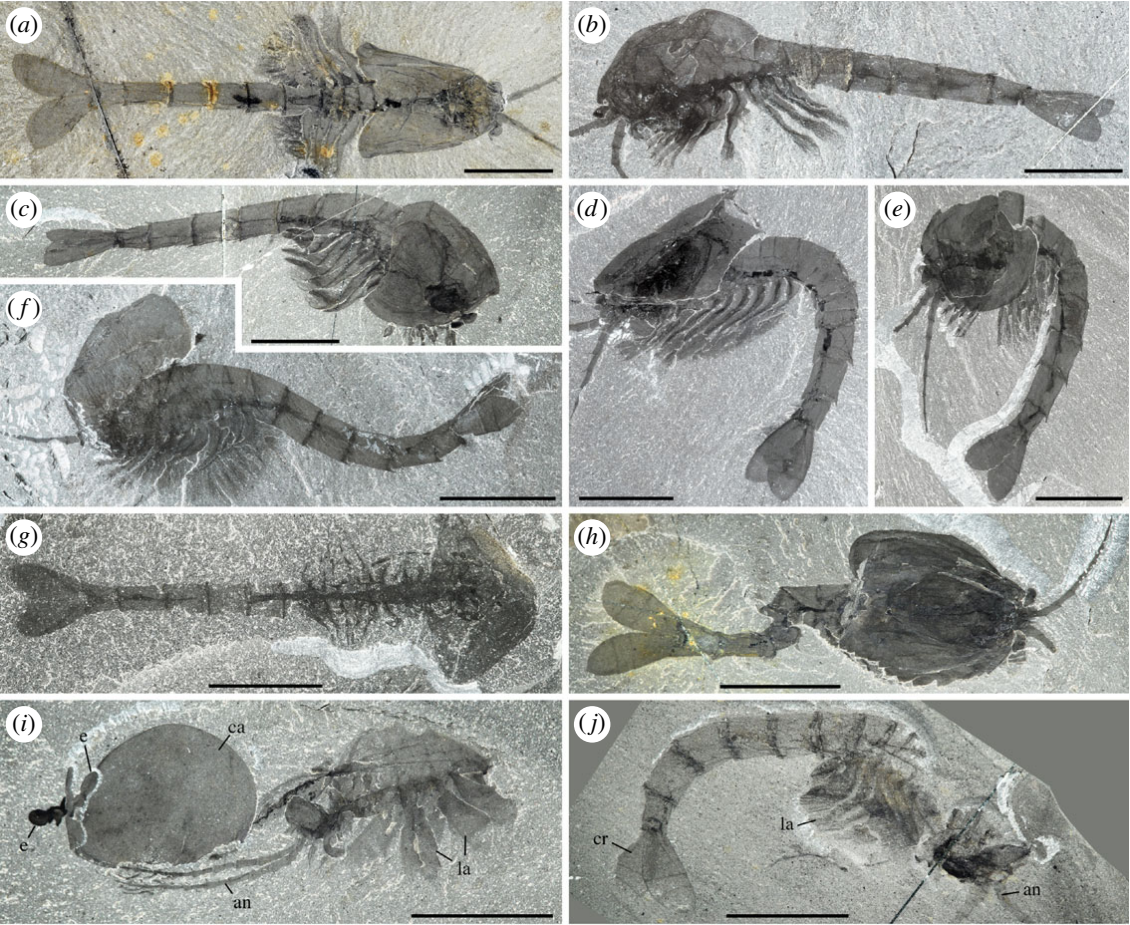
*Waptia fieldensis* Walcott, 1912 [10] from the middle Cambrian (Series 3, Stage 5) Burgess Shale, British Columbia, Canada; preservational aspects. (*a,b*) USNM 57681a, USNM 139214; posterior part of body with a straight profile in dorsal and lateral views, respectively. (*c–f*) ROMIP 56421, USNM 268270, ROMIP 64281, USNM 529131, body with slightly curved, recurved and sigmoidal profile in lateral view. (*g*) ROMIP 64282 with carapace tilted downwards and most swimming (lamellate) appendages decayed. (*h*) ROMIP 64283, with broken abdominal region. (*i*) ROMIP 56427, specimen with disarticulated cephalic elements (carapace detached from the rest of the body; see also figure 6). (*j*) ROMIP 64284 with the anterior part detached from the rest of the body. All images are photographs taken under cross-polarized light. Abbreviations are as follows: an, antennule; ca, carapace; cr, caudal ramus; e, eye; la, lamellate post-cephalothoracic appendage. Scale bars: 1 cm.

**Figure 2.**
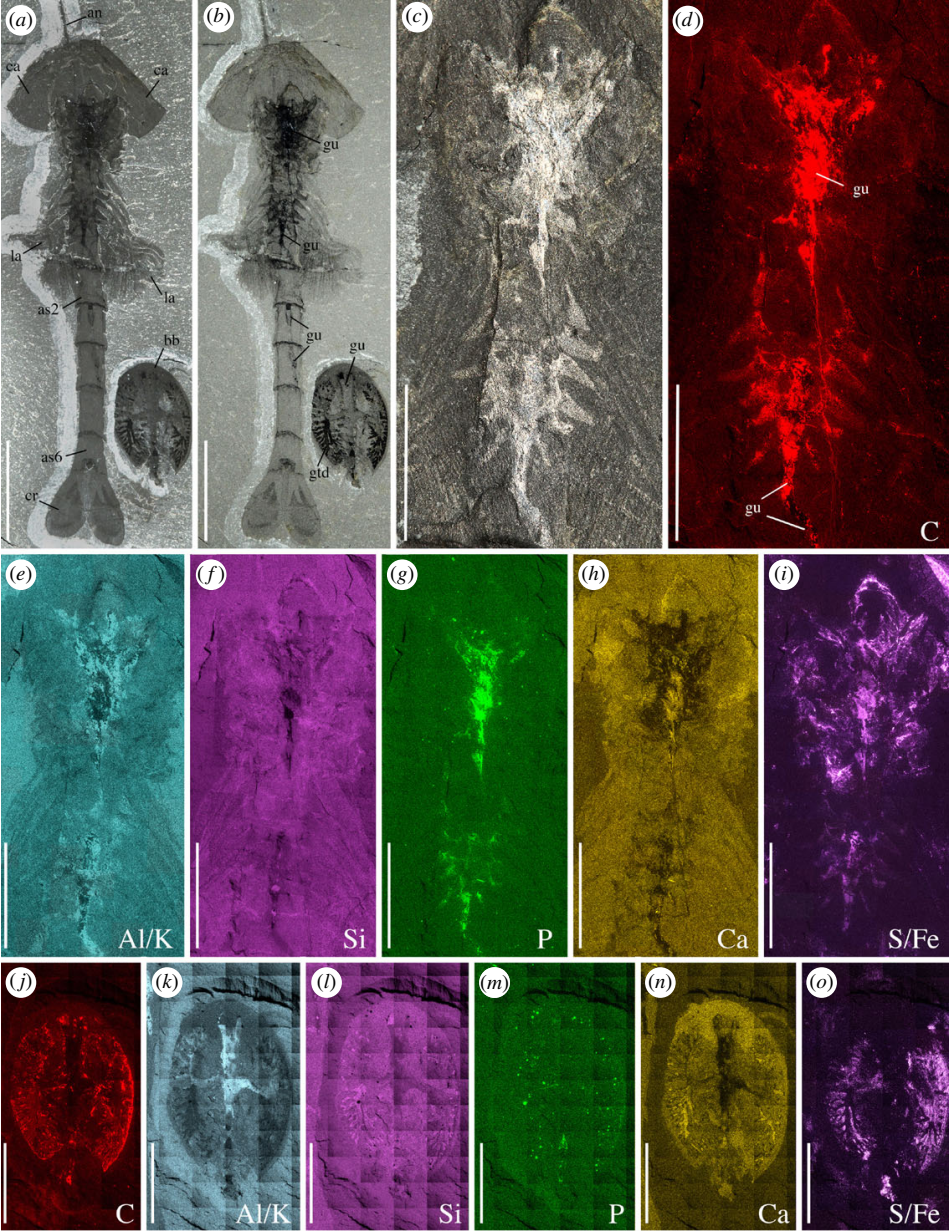
*Waptia fieldensis* Walcott, 1912 [10] from the middle Cambrian (Series 3, Stage 5) Burgess Shale, British Columbia, Canada. ROMIP 64295, elemental maps. (*a–c*) General views and details of cephalothoracic and post-cephalothoracic regions in polarized light. (*d–i*) Elemental maps of the cephalothoracic and post-cephalothoracic regions. (*j–o*) *Burgessia bella* (see location in *a,b*; Arthropoda; [17]). Elemental maps of *Waptia fieldensis* and *Burgessia bella* are provided to show that both arthropod species preserve in similar ways. Abbreviations are as follows: Al, aluminium; an, antennule; as, abdominal segment; bb, *Burgessia bella*; C, carbon; Ca, calcium; ca, carapace; cr, caudal ramus; Fe, iron; gtd, gut diverticula; gu, gut; K, potassium; la, lamellate post-cephalothoracic appendages; P, phosphorus; Si, silicon; S, sulfur. Scale bar: 1 cm in *a,b*; 5 mm in *c–o*.

*Waptia fieldensis* is typically preserved like other Burgess Shale-type fossils [39–41] as two-dimensional carbonaceous and aluminosilicate films (figures 1 and 2). Topological variations of these films across the body suggest potential original variations in tissue composition and diagenetic history. For example, exoskeletal features, such as the carapace, are mainly replicated in silicon and calcium while the original carbon film is only faintly visible as a result of diagenetic volatilization (figure 2). However, internal structures tend to be richer in carbon and tend to be partially or entirely replicated in aluminium, potassium and phosphate, suggesting that such structures might have mineralized early on. Some structures might also be more prone to partial pyritization (sulfur + iron; figure 2). Different internal organs tend to preserve in similar ways and teasing them apart based on their elemental signatures alone is difficult. The internal part of the eyes (optic neuropils) is rich in carbon.

Specimens show nearly all possible configurations within the matrix (figure 1), the most frequent one being that of the animal lying laterally with its sagittal plane parallel to the bedding. Other specimens are oriented with their sagittal plane perpendicular to the bedding, revealing details of their dorsal or ventral morphology (e.g. appendages). The prevalence of preservation in lateral aspect probably stems from the fact that the body of *W. fieldensis* was laterally compressed as in various modern shrimps in contrast to a number of dorsoventrally flattened Burgess Shale arthropods such as trilobites, *Sidneyia* and *Naraoia*. Some specimens are preserved in various intermediate and oblique positions, including near-frontal views which would be consistent with transport within a turbulent flow as previously interpreted for other Burgess Shale organisms such as *Marrella splendens* [42,43]. Straight (figure 1*a,b*), slightly curved (figure 1*c*), sigmoidal (figure 1*f*) or recurved (figure 1*d*,*e*) profiles may also represent a combination of life attitudes at the time of burial, escape reactions during or after entombment, or muscle relaxation or tissue degradation immediately after death. Possible reactions to burial have also been suggested for other Burgess Shale animals (e.g. [5]).

Specimens showing varying degrees of decay and disarticulation are frequent. The carapace is often displaced, tilted downwards (figure 1*f*,*g*) or torn free from the body (figure 1*i*,*j*), exposing underlying appendages. Displacements of internal features and appendages are also common. These configurations most probably result from the effect of decay prior to burial, limited transport and possibly minor displacements during final entombment (e.g. *in situ* dissociation [44]). Taphonomy experiments conducted with *Nebalia bipes* (Crustacea, Malacostraca, Leptostraca) show that decay quickly results in the detachment and rotation of the carapace (electronic supplementary material, S2) as seen in fossils. All in all, these patterns provide support for low- to relatively high-energy depositional events that smothered a life assemblage at or above the water–sediment interface and at the same time entombed partially decayed specimens (death assemblage) deposited on the bottom, consistent with results from quantitative biostratinomic analyses [41].

*Waptia fieldensis* ranks as the 10th most abundant arthropod species in the Walcott Quarry overall, and represents an indicator species of a group of bedding assemblages containing a particularly diverse and well-preserved fauna relative to other bedding assemblages from the Walcott Quarry (Group 3; see [28]). *Waptia* occurs alongside a variety of other animals such as the chordate *Pikaia*, the arthropods *Alalcomenaeus*, *Molaria*, *Plenocaris*, *Marrella* and *Burgessia*, the enteropneust worm *Spartobranchus*, the lobopodian *Aysheaia* and the polychaetes *Peronochaeta*, *Burgessochaeta* and *Canadia*. Several rock slabs showing multiple individuals of *W. fieldensis* preserved together (electronic supplementary material, S3) may suggest a gregarious habit.

## 5. Palaeontological descriptions and discussions

### 5.1. Systematic palaeontology

Phylum Arthropoda von Siebold, 1848 [45]

Phylum Euarthropoda Lankester, 1904 [46]

Subphylum Mandibulata Snodgrass, 1938 [47]

Order Hymenocarina Clarke, 1882 (emended Raymond 1935) [48]

*Diagnosis (emended from Aria & Caron, 2017* [9])*.* Mandibulate euarthropods with the following characters: bivalved carapace with a highly convex cross section covering the cephalothoracic region; cephalothorax bearing well-developed multisegmented antennules and endopods with well-developed paired terminal claws; limb basis enditic and externally subdivided; frontalmost inter-ocular complex composed of a median sclerite flanked by lobate protrusions; post-antennular pair of appendages generally not developed; mandibles broad and rounded with uniform masticatory margins; posterior tagma (abdomen) with segments forming tergo-pleural rings; tailpiece bearing well-developed caudal rami.

*Taxa included.* Protocarididae Miller, 1889 (emended Aria & Caron, 2017 [9]), Waptiidae Walcott, 1912 [10], *Plenocaris* Walcott, 1912 [10], Canadaspididae Novozhilov, 1960 [49]), Odaraiidae Simonetta & Delle Cave, 1975 [20], Perspicarididae Briggs, 1978 [50], *Clypecaris pteroidea* Hou, 1999 [51], *Jugatacaris agilis* Fu & Zhang, 2011 [52], *Nereocaris* Legg *et al.* 2012 [53]. Possibly includes: *Pectocaris spatiosa* Hou, 1999 [51], *Yunnanocaris megista* Hou, 1999 [51], *Occacaris oviformis* Hou, 1999 [51], *Forfexicaris valida* Hou, 1999 [51] and *Clypecaris serrata* Yang *et al.* 2016 [54]. We do not include ‘*Loricicaris*’ Legg & Caron, 2014 [55] because this name is not currently properly established (see Aria & Caron, 2017 [9], electronic supplementary material, Discussion).

Family Waptiidae Walcott, 1912 [10]

*Diagnosis* (*emended from Walcott 1912* [10])*:* Hymenocarines with the following characters: elongate body capped anteriorly by a bivalved carapace with an elliptical outline in lateral view. Body made of 21 somites organized into three tagmata: (i) a cephalothorax of nine somites composed of a pair of large sub-ovate lateral eyes, a median triangular sclerite, one pair of long multisegmented antennules, one pair of mandibles, one pair of maxillules and four pairs of uniramous appendages; (ii) a post-cephalothorax bearing six pairs of annulate appendages fringed with numerous lanceolate lamellae; (iii) an abdomen made of five limbless, cylindrical segments and a tailpiece bearing a pair of flattened caudal rami.

*Genera included: Waptia* Walcott, 1912 [10]; *Pauloterminus* Taylor, 2002 [56]; *Chuandianella* Hou & Bergström 1991 [57–59]; possibly *Synophalos* Hou *et al.* 2009 [60,61].

*Type genus: Waptia* Walcott, 1912 [10].

*Type species: Waptia fieldensis* Walcott, 1912 [10].

*Species included:* Only *Waptia fieldensis* Walcott, 1912 [10].

*Diagnosis* (*emended from Walcott, 1912* [10])*.* Waptiid arthropod with the following characters: smooth bivalved carapace covering most of the cephalothorax (no external dorsal split or hinge-like structure); inter-ocular area bearing median triangular sclerite flanked by two lobe-like body projections; antennules with ten elongated podomeres bearing stiff setae at their distal margins; rounded mandibles with three-segmented setose palps; maxillules stenopodous with distal podomere bearing multiple setae and a pair of claws; post-maxillular section composed of four pairs of appendages; first three pairs made of five-segmented endopods (including distalmost claws) attached to four-segmented basipods bearing elongate endites; fourth pair with five-segmented endopod attached to an annulate basis bearing short lamellae; tip of all four post-maxillular cephalothoracic endopods with two main claws atop a set of several recurved spines and straight setae; post-cephalothoracic somites five and six fused into one segment, so that post-cephalothoracic segment five bears two pairs of lamellate appendages; proximalmost portion of post-cephalothoracic appendages covered by sclerite; lobate caudal rami subdivided into three subequal pieces.

*Waptia fieldensis* Walcott, 1912 [10].

See figures 1–28; electronic supplementary material, S1, S3–S6, S8, S11–S14, S16–S22; S23, S24 (videos)

*Synonymy:* See list in electronic supplementary material, S4.

*Type material:* The original description of *Waptia fieldensis* by Walcott ([10]; pl. 27, figs 4,5) is based on two complete figured specimens (USNM 57681 and 57682; electronic supplementary material, S5). None of them was designated as a holotype. Five additional specimens (USNM 83948a, b, c, d, e) were chosen by Walcott [18, p. 24] as syntypes in a more detailed description of this species. It is Hughes *in* Conway Morris [21] who formally established USNM 57681 to the rank of lectotype.

*Occurrence: Waptia fieldensis* occurs through a stratigraphic succession of about 150 m within the Burgess Shale (‘thick’ Stephen) Formation (Cambrian Series 3, Stage 5; electronic supplementary material, S1). The great majority of specimens are from the Walcott Quarry (Greater Phyllopod Bed) on Fossil Ridge, about 2 km north of Field in Yoho National Park, southeast part of British Columbia, Canada. Outside of the Burgess Shale, *Waptia* cf. *fieldensis* occurs in the younger Spence Shale Member of the Langston Formation, along the west side of the Wellsville Mountains near Brigham City, Box Elder County, Utah [62].

### 5.2. Morphological description and discussion

To preserve the flow of reasoning, the description of each key morphological feature is followed by a short discussion rather than two distinct description and discussion sections.

#### 5.2.1. Size and sexual dimorphism

Only complete non-disarticulated specimens lying parallel to the bedding plane and preserved in lateral, dorsal or ventral aspects were used for accurate measurements of the body and carapace of *W. fieldensis*. The carapace length (Lc) and the total body length (Lb) were measured in a subset of complete specimens (*N* = 28; videos in electronic supplementary material, S6) showing varying degrees of body curvature. Lb is defined as the length of the curve that links the median triangular sclerite to the tips of the caudal rami (electronic supplementary material, S6b). Lb varies from *ca* 13.5 to 66.5 mm with 85% of specimens ranging between 40 and 60 mm. The Lc to Lb plot diagram (electronic supplementary material, S6c) shows four small-sized specimens interpreted as juveniles (with around Lb < 45 mm) and a cluster of subadult or adult individuals spread around the regression line. While the adjusted *R*^2^ for the entire data is 0.77, it is only 0.41 when juveniles are removed, suggesting the presence of more than one Gaussian population. This is confirmed by a Kernel density representation of Lc/Lb, which shows clear bimodality. The non-overlapping separation between points with positive and negative residuals further suggests the presence of two sub-morphs, and therefore distinct groups of subadult or adult specimens interpreted as a possible sexual dimorphism. One complete specimen carrying eggs [16] also plotted in the diagram shows that individuals with positive residuals would be females, while the negative residuals would represent males. A one-way ANOVA finds sex to be a very significant factor of the Lc/Lb ratio (*p*-value 5.00×10^−10^), and a MANOVA on Lc and Lb likewise finds sex to be a very significant discriminator (*p*-value 6.75×10^−9^). For the same body length value, the carapace of females tends to be relatively longer than that of males. Comparable carapace dimorphism occurs in extant crustaceans such as *Nebalia* (e.g. [63]). Additional detailed observations would be required using a larger sample size to detect other potential dimorphic characteristics other than the length of the carapace. Such observations should also be conducted at the scale of individual bedding assemblages to evaluate the amount of variability that existed within particular populations or subpopulations, but such studies are beyond the scope of this paper.

#### 5.2.2. Carapace

**Figure 3.**
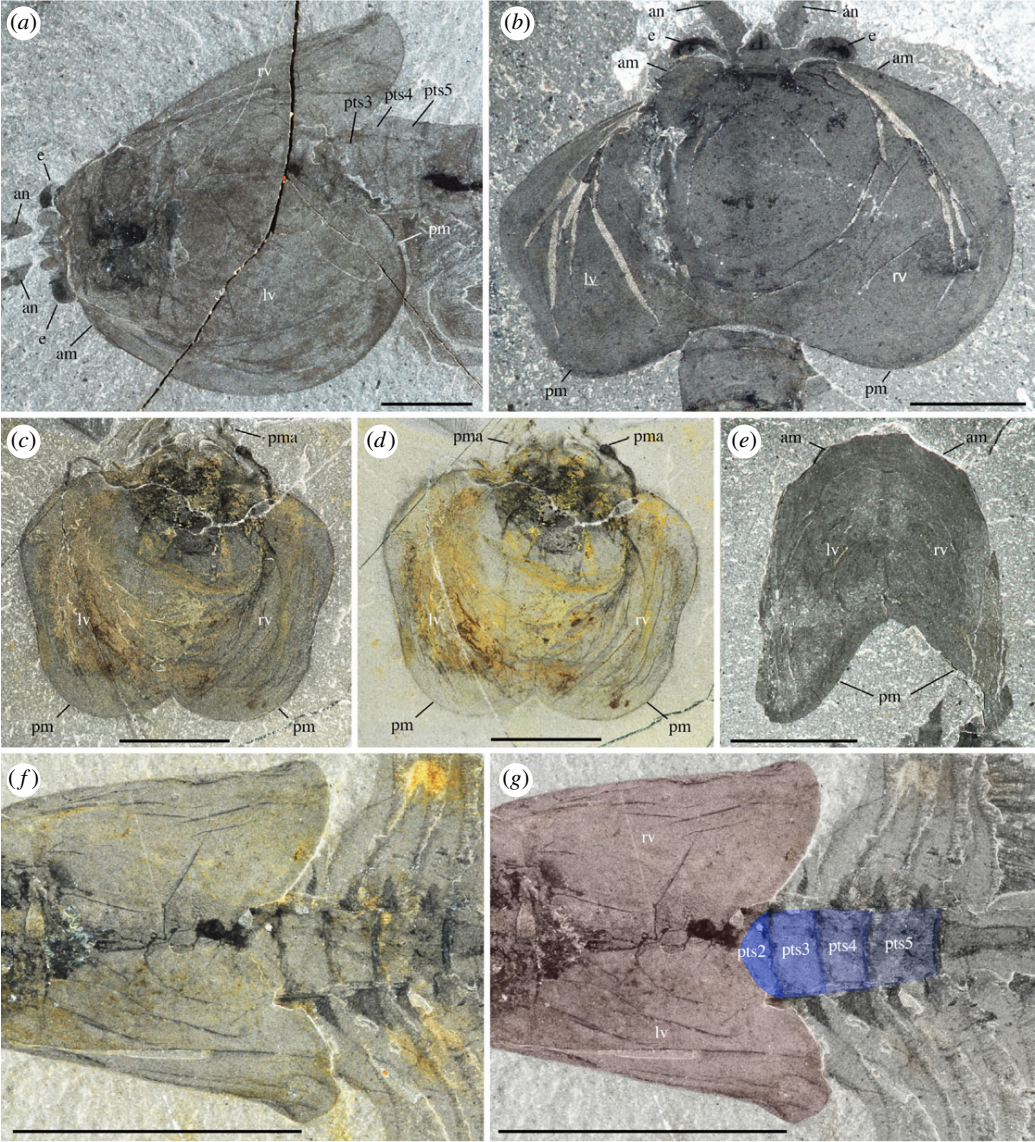
*Waptia fieldensis* Walcott, 1912 [10] from the middle Cambrian (Series 3, Stage 5) Burgess Shale, British Columbia, Canada; carapace outline. (*a*) USNM 114251 showing the left valve in an oblique-lateral view. (*b*) ROMIP 64286 with dorsoventrally compressed carapace (left and right valves). (*c,d*) ROMIP 64385; carapace in an intermediate frontal view. (*e*) ROMIP 64285, isolated complete carapace, tilted downwards in dorsal view. (*f,g*) USNM 57681a showing the indentation of the posterodorsal margin of the carapace (carapace and thoracic segments highlighted in red and blue, respectively). All images are photographs taken under cross-polarized light (*d*, immersed in water). Abbreviations are as follows: am, anterior margin; an, antennule; e, eye; lv, left valve; pm, posterior margin; pma, post-maxillular cephalothoracic appendage; rv, right valve; pts1–5, 1st to 5th post-cephalothoracic segments. Scale bars: 1 cm in *f,g*; 5 mm in *a–e*.

The ‘bivalved’ carapace of *W. fieldensis* is best described as a non-mineralized, thin and saddle-like cuticular structure extending over the anterior part of the body and offering a lateral protection mostly to the cephalothorax as well as for brooding eggs and embryos [16]. Its outline in lateral view is sub-elliptical, tapers anteriorly and slightly expands posteroventrally into a slightly wider and more rounded lobe (figure 3*a*,*e*). The dorsal margin is slightly convex and terminates at approximately 90° with both the anterior and posterior margins. Dorsoventrally flattened specimens exposed in dorsal view (e.g. figure 3*e*) show no distinct boundary such as a hinge line or a cuticular split between the right and the left valves. The fusion of the valves is corroborated by the fact that they are never found separated, even in specimens with obvious carapace displacement. The right valve is a mirror image of the left one (figure 3*c*,*e*). The carapace seems to be attached to the body via a relatively narrow area near the anterior end of the head (figure 3*a*,*c*,*d*). This narrow attachment zone is also suggested by the fact that the posterior end of the carapace tends to be tilted upwards in many specimens, while the front end retains a tighter connection with the body (e.g. figure 1*f*). The carapace loosely covers most of the anterior part of the body. In dorsal view, the posterodorsal margin of the carapace forms a relatively deep indentation reaching the second post-cephalothoracic segment bearing the second pair of lamellate appendages (figure 3*a*,*e*,*f*; see definition of post-cephalothorax in §5.2.3). In lateral view, the valves cover up to the fourth or fifth post-cephalothoracic segment. In an idealized transverse section the carapace would appear as a continuous, inverted parabolic structure, but the two-dimensional preservation of the fossil specimens did not allow for accurate measurements of its lateral convexity and opening angle. Lateral variations in width with the valves tucked closely around the body or far apart from it (e.g. figures 1*a* and 3*b*) suggest that the valves were thin. Despite the lack of a dorsal hinge line, they could flex relative to each other. Frequent wrinkles and concentric folds (figure 3*b*–*d*) also confirm that the carapace was non-mineralized and flexible. This type of carapace may, for instance, be compared with that of extant leptostracan crustaceans such as *Nebalia bipes* (electronic supplementary material, S7a,c). The carapace of *N. bipes* is translucent (electronic supplementary material, S2a) with an around 5 µm thick external outer lamella made of laminated layers of chitin (electronic supplementary material, S7d,e), separated from the inner lamella by epidermal cells. Unlike *Nebalia*, however, the frontal part of the carapace of *W. fieldensis* lacks a hinged rostral plate (electronic supplementary material, S2a and S7a,b).

#### 5.2.3. Tagmatization

The body of *W. fieldensis* can be divided into three broad regions: (i) a cephalothorax encompassing prominent stalked eyes, long antennules, mandible-like gnathal appendages with a three-segmented palp, stenopodous appendages interpreted as maxillules, followed by a series of four uniramous appendages, the three anterior ones bearing proximal endites; (ii) a post-cephalothorax of five segments bearing six pairs of homonomous appendages fringed with elongate lamellae; (iii) an abdomen characterized by limbless ring-shaped segments and flattened caudal rami. As is often the case in extant euarthropods, this subdivision in tagmata coincides with groupings of appendages and segments into morphofunctional units: anterior tagma for sensing and feeding, middle tagma for respiration and swimming and posterior abdominal tagma for locomotion and steering (see discussion on lifestyles). The cephalothoracic, post-cephalothoracic and abdominal tagma represent approximately 15%, 20% and 65% of the total body length, respectively.

The cephalothorax itself would comprise two tagmata, that is, the cephalon and the thorax; however, the boundary between the two is difficult to establish based on external anatomy only. In particular, it is unclear whether the first pair of enditic limbs (coined pma1 below) should be considered a ‘maxilla’ and thus be part of the head tagma. If this is the case, *Waptia* would possess a diagnostic mandibulate head with six somites, as likely is the case in protocaridids [9]. This would also be the case if an additional, reduced maxilla were present behind the maxillules but difficult to identify in the fossils. With such a configuration, the body tagmatization of *Waptia* would be almost identical to that of malacostracans (see section on phylogeny below).

#### 5.2.4. Ocular and inter-ocular regions

**Figure 4.**
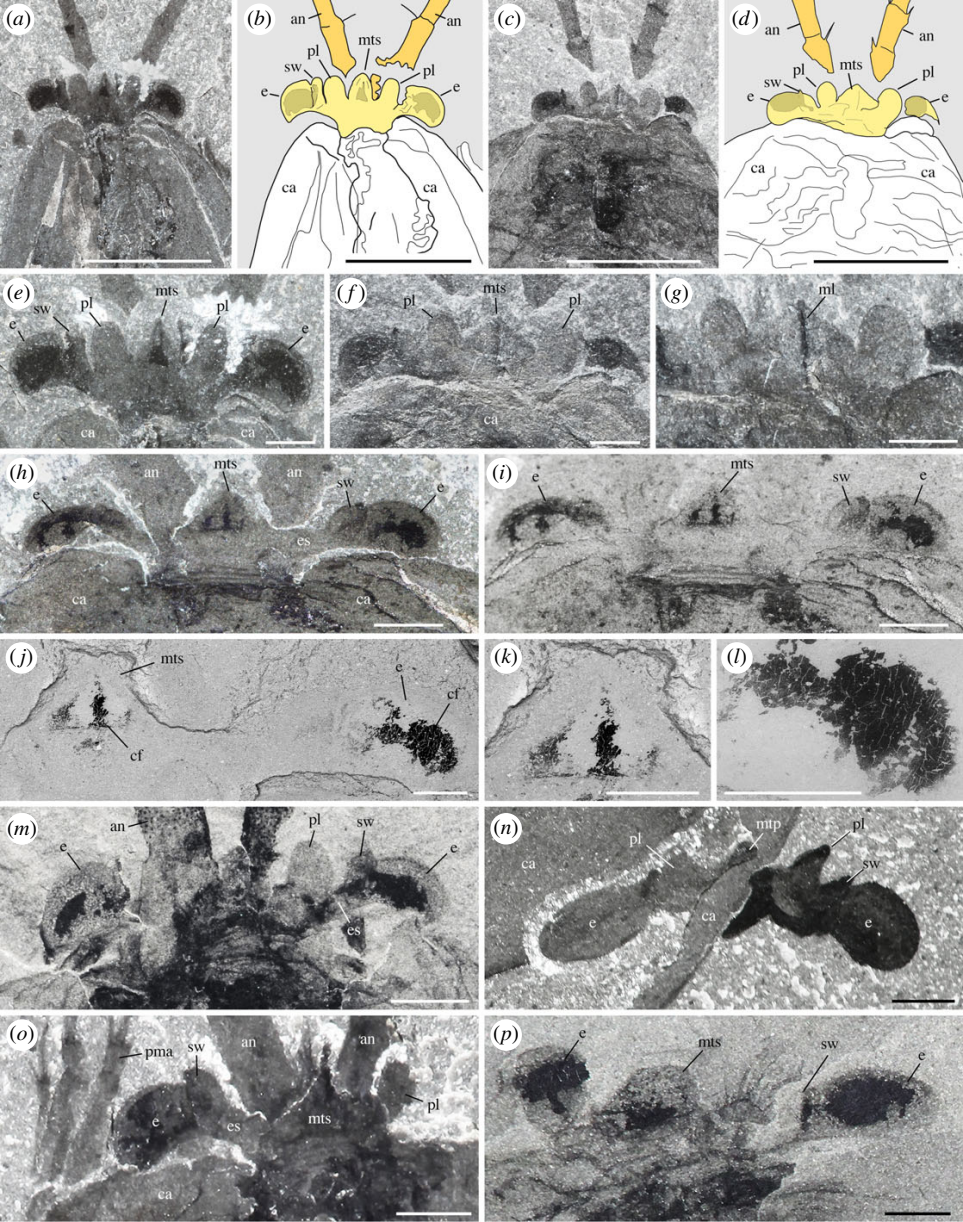
*Waptia fieldensis* Walcott, 1912 [10] from the middle Cambrian (Series 3, Stage 5) Burgess Shale, British Columbia, Canada; ocular and inter-ocular regions. (*a,b,e*) USNM 83948j in dorsal view. (*c,d,f,g*) USNM 114251 in dorsal view. (*h–l*) ROMIP 64286, general dorsal views and details showing remains of carbonaceous films (dark areas) within eyes and median triangular sclerite. (*m*) USNM 138231 in ventral view. (*n*) ROMIP 56427, ocular and inter-ocular regions detached from the body (see also figure 1*i*). (*o*) ROMIP 64283 in dorsal view. (*p*) ROMIP 64287, details of dark areas in eyes and the median triangular sclerite (see also figure 2*b*). *a,c,e,g,h* and *m–p* are photographs taken under cross-polarized light; *i–l, n, p* are backscattered SEM images. Ocular and inter-ocular regions and antennules in yellow and light orange, respectively. Abbreviations are as follows: an, antennule; ca, carapace; cf, carbonaceous film; e, eye; es, eye stalk; ml, median line; mts, median triangular sclerite; pl, peduncular lobe; pma, post-maxillular cephalothoracic appendages; sw, swelling. Scale bars: 5 mm in *a–d* and *n*; 1 mm in *e–i*, *m*, *o* and *p*; 500 µm in *j–l*.

The frontalmost body part of *W. fieldensis* forms a laterally widened unit bearing three elements: a pair of prominent kidney-shaped eyes, a pair of small lobe-like projections and a median triangular sclerite covering remains of soft tissues. This frontal unit protrudes beyond the anterior margin of the carapace as seen in specimens preserved in dorsal view (figure 4*a*–*e*,*h*; electronic supplementary material, S8), and clearly sits above and in front of the basal part of the antennules.

##### 5.2.4.1. Eyes

*Description.* Eyes have a reniform shape (long axis around 1 mm). They extend beyond the carapace margin anteriorly and laterally and display a relatively wide hemispherical section. Each eye is mounted on a short undivided probably cylindrical stalk. The distal part of the stalk is slightly enlarged to accommodate the eye lobe and forms a small anterior rim-like swelling (figure 4*a*–*e*,*i*,*m*–*o*). The central area of the eye lobes and the internal part of the eye stalks often contain remains of highly reflective carbon films which appear as black oval or more elongated features under crossed polarized light or backscattered electron microscopy, and which probably correspond to the preservation of neural tissues (i.e. neuropils; see below).

**Figure 5.**
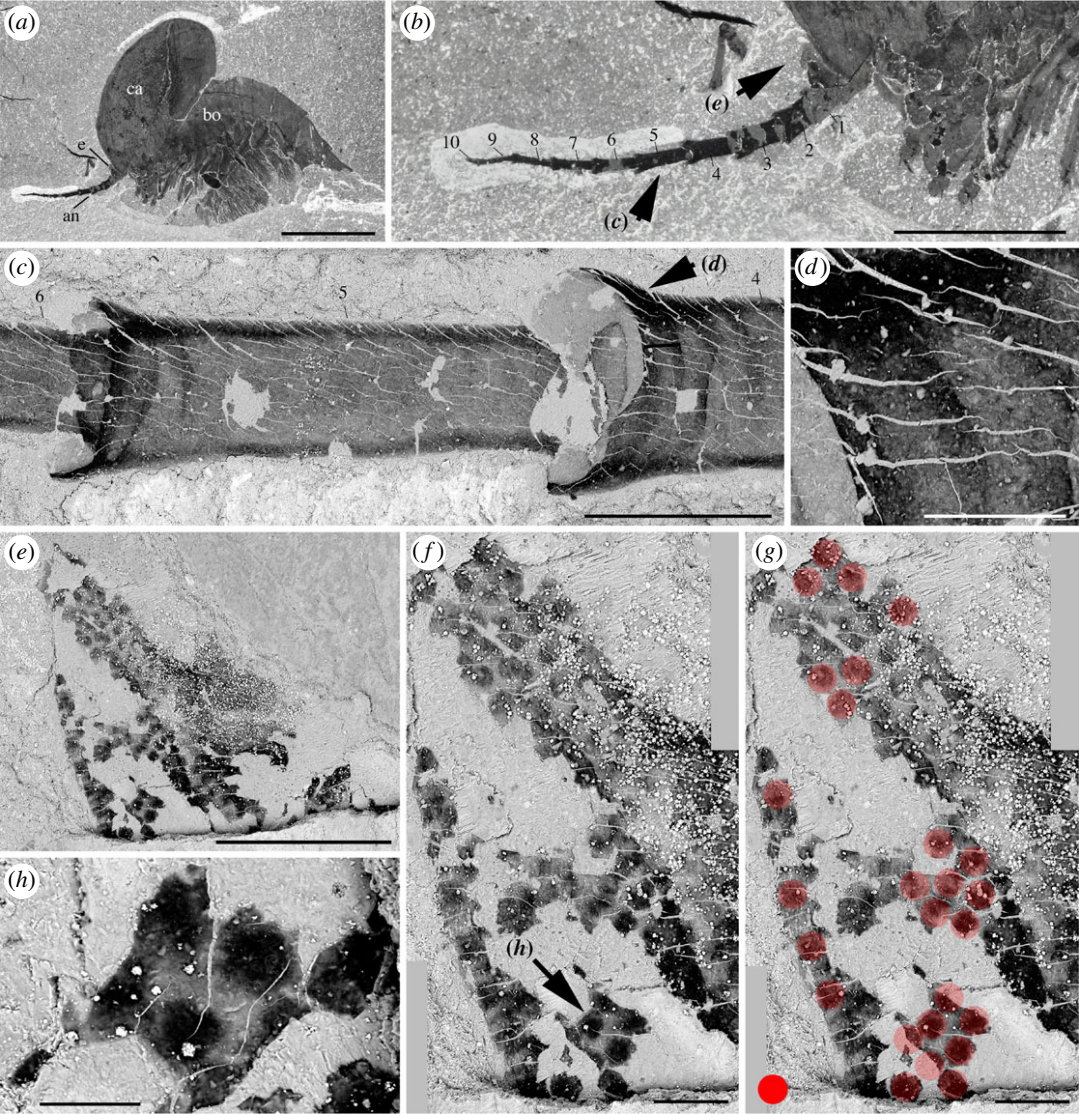
*Waptia fieldensis* Walcott, 1912 [10] from the middle Cambrian (Series 3, Stage 5) Burgess Shale, British Columbia, Canada; details of eye and antennular cuticular features in ROMIP 64288. (*a*) General view. (*b*) Antennule. (*c,d*) Details of antennular podomeres showing thickened carbon films along margins. (*e*) General view of eye (see *b* for location). (*f–h*) Black spots of carbon film interpreted as the remains of ommatidial structures. *a,b* are photographs taken under cross-polarized light. *c–h* are backscattered SEM images. Red dots highlight the outline of assumed ommatidia. Abbreviations are as follows: an, antennule; bo, body; ca, carapace; e, eye; 1–10, antennule podomeres 1 to 10. Scale bars: 1 cm in *a*; 5 mm in *b*; 500 µm in *c* and *e*; 100 µm in *d,g* and *h*; 50 µm in *f*.

The eye of a single specimen (ROMIP 64288, figure 5) shows closely packed circular spots (around 40 µm in diameter) made of carbonaceous films. These structures are interpreted here as the compressed remains of external ommatidial structures, possibly corneal lenses lined with a very thin cuticle. This represents the first direct and unambiguous evidence of ommatidia reported from the Burgess Shale. Systematic observations of the eyes of additional specimens using SEM would undoubtedly reveal additional evidence in the future. Underlying structures, such as receptor cells, are not preserved. The concentration of facets over the spherical visual surface of *W. fieldensis* may be estimated to around 600 per mm^2^ (approximation based on SEM images; figure 5). Facets with a comparable diameter and density occur in extant crustaceans such as *Nebalia* (e.g. [58], fig. 2). However, it is impossible to estimate the total number of ommatidia per eye in *W. fieldensis* and no information is available on the underlying neural features (e.g. [64]).

*Discussion.* Although the visual performance of *W. fieldensis* cannot be inferred from our fossil specimens, the location, orientation and hemispherical shape of the eyes (figure 4*a*,*h*,*m*) suggest that this arthropod had a relatively large frontal and lateral field of vision and the capacity to explore its environment. The mobility of the eyes is conjectural because no articulation is visible between the eye stalk and the frontal part of the body. Compound eyes are reported to occur in *Chuandianella ovata,* an early Cambrian waptiid from the Chengjiang biota ([65]; pl. 1f) which closely resembles *W. fieldensis*. However, the clustered, rounded (diameter 33–45 µm) features distributed over the visual surface in *C. ovata* are more likely to be iron oxide artefacts derived from original pyrite than original lenses (see [65], plate 1, figs f1, f2). Inferring the visual acuity, sensitivity and ecological adaptation of waptiid eyes [60] from such fossil specimens remains problematic.

Small hair-like features regularly distributed along the convex margin of the eye of USNM 268199 were considered by Strausfeld [13] as possible indicators of a layer of closely packed vertically arranged lenses. He proposed that *W. fieldensis* may have had 120 ommatidia per eye. Although numerous extant insects (but no crustaceans) do have small inter-ommatidial setae, the re-examination of weathered USNM 268199 (electronic supplementary material, S8e,g) indicates that these hair-like features are most probably artefacts. The yellowish minerals covering the entire body most likely result from the weathering of ferrous iron. The setae-like structures would then represent a by-product of alteration—possibly microdendrites of manganese oxide—which occur elsewhere on the appendages and carapace. Strausfeld ([15], fig. 3) also described putative distorted lenses along the eye convex margin of USNM 83948j (erroneously recorded as USNM 57682). New observations of this specimen under cross-polarized, normal light (figure 4*a*,*b*; electronic supplementary material, S8a,b) and SEM do not support this interpretation. Neither the left nor the right eye is lined with regularly spaced well-delimited lens-like features. Instead, the lenticular aspect seems to be attributable to the irregular preservation of the distalmost neuropil (see section on nervous system, below).

##### 5.2.4.2. Frontal projections

*Description.* A pair of small but thick lobe-like projections, about 1 mm in length, with a broad base and an elliptical outline (figure 2*e*–*g*,*m*) slightly overhangs the eye stalks. They seem to be relatively flat and featureless and do not show any basal folded or jointed feature, suggesting that they are projections from the inter-ocular region of the body itself.

A median triangular projection as long as the pair of peduncular lobes (figure 4*f*–*h*) sits in the central part of the inter-ocular unit. This broad-based pointed element is lined with a cuticle but is not attached to the anterior margin of the carapace unlike, for example, the rostral plate of *Nebalia bipes* (electronic supplementary material, S7a and S7b). In the dorsal view, this projection often shows a median split or carina (figure 4*g*). A triangular patch of carbonaceous films, possibly bilateral, often concentrate in its central part on both sides of a central low-relief axis (figure 4*j*–*l*) or form a more irregular central deposit (figure 4*p*).

Discussion.

*Peduncular lobes.* Seemingly homologous para-ocular features occur in the hymenocarine *Canadaspis perfecta* (Walcott, 1912) [10,50], although they have an elongate morphology in this taxon. Comparable but probably convergent lobate projections also occur in a variety of stem and crown-group representatives of Euarthropoda such as *Pambdelurion*, *Cambropycnogon* (Cambrian pynogonid), *Tanazios* (as argued by Boxshall [66]) and extant remipede crustaceans (e.g. [67]). Although authors have generally assumed they were sensory organs (e.g. [50,68]), the segmental origin of these lobes has been debated. While Briggs [50] had originally described them as crustacean antennules (A1) in *Canadaspis*, Aria & Caron [69] suggested that these paired projections in hymenocarines may represent vestigial homologues of the frontalmost appendages of more basal panarthropods, arising from the ocular protocerebral somite. Ortega-Hernández & Budd [68] regarded those features across panarthropods as deeply homologous and also assigned them to the protocerebral somite, but considered them as non-appendicular in origin.

The presence of identical peduncular lobes in *Fuxianhuia* and its relatives (e.g. [70]) indicates that this feature was shared among early mandibulates (see below), and thus was more than an occurrence of parallelism between distantly related taxa. Although their developmental origin remains unclear, the shape and location of these peduncular lobes suggest that they may have accommodated hemi-ellipsoid bodies, as do extant crustaceans, as we discuss in §5.2.9.1.

*Median triangular projection.* Distinct from the peduncular lobes, the median triangular projection of *W. fieldensis* is probably homologous with the so-called ‘anterior sclerite’ of other Cambrian arthropods [58,70,71]. It is also similar, although smaller, to the sub-triangular frontal sclerite of protocaridids [9]. Importantly, protocaridids display paired dark patches underneath this structure, as does *W. fieldensis*, in which the underlying traces are often highly reflective (figure 4*e*,*h*–*k*,*p*). The artiopodan *Helmetia expansa* Walcott, 1918 [72] was also shown to possess such a sclerite [71], likewise showing paired highly reflective spots interpreted as the remains of neural tissues, possibly the equivalent of frontal sensory organs. Although we cannot confirm the presence of an anterior sclerite in *Odaraia alata* Walcott 1912 [10], this bivalved arthropod sports a triplet of tiny reflective spots [71] that may recall the median eyes of present-day crustaceans such as Branchiopoda [73,74]. An anterior sclerite is also present in *Canadaspis* [9,51], but underlying tissues have not yet been described.

Strausfeld [15] observed reflective spots within the triangular projection of *W. fieldensis* and interpreted them as the remains of a sensory organ possibly homologous with the insect ocelli and the crustacean nauplius eye. Although a sensory function is plausible, specific comparisons with ocelli or the nauplius eye are more conjectural. USNM 268199 chosen by Strausfeld ([15], fig. 3B; camera lucida drawing) to support the presence of ocelli does not show any clear subdivision (electronic supplementary material, S8a–d). The nauplius eye of extant branchiopod crustaceans such as *Lynceus* [74,75] does have a similar frontal location, an overall triangular shape and bilateral symmetry, and houses a complex of four cups (including two reniform lateral ones) separated from one another by pigment layers. Whether the symmetrical elements within the median triangular projection structures of *W. fieldensis* are subdivided into smaller structures cannot be determined.

Recently, Aria & Caron [9] interpreted the bipartite frontal protrusion of the early mandibulate *Tokummia* and *Branchiocaris* as a possible labrum retaining an appendicular morphology. The labrum is a flap-like structure located just in front of the mouth of most extant Euarthropoda and is a key functional element of the mouth parts of numerous crustaceans (e.g. myodocopid ostracods and isopods; electronic supplementary material, S9 and S10) and insects. The backward migration of the labrum from the anteriormost location [76,77] and expression of homeotic genes during embryogenesis [78,79] suggest indeed that the labrum of early arthropods may have been a frontal appendicular structure [9]. The labrum is also known to have a bipartite external and internal configuration in the adults of certain extant myodocopid ostracods such as *Vargula* (e.g. [80]; electronic supplementary material, S9). The frontal structure of protocaridids was also compared to the chelicerate epistome-labrum, similarly composed of a dorsal/anterior sclerite and of a fleshy outgrowth in the form of bipartite ‘lips’ in some arachnid orders [81].

This evidence suggests that the median triangular projection and associated tissues of *W. fieldensis* are part of a ‘labral complex’ in which sensory organs would be closely associated with the fleshy protrusion of a crustacean-like labrum proper, as is also the case in ‘Orsten’ crustaceomorph larvae (e.g. [82]) and perhaps also in agnostids [83].

*Implications.* The perspective provided by the redescription of *W. fieldensis* has two main consequences. First, *Waptia* firmly demonstrates that the frontalmost, median sclerite and its associated tissues constitute an important and conserved anatomical feature during the early radiation of mandibulates, and is arguably inherited from more ancestral euarthropods. Second, *Waptia* shows that another anterior feature, here termed the peduncular lobes, is also present across Cambrian taxa that may have had a close phylogenetic relationship during the emergence of Mandibulata.

As it remains difficult to directly trace their origin among stem-group euarthropods (in particular, lobopodians and radiodontans), it seems that these frontalmost features could be secondarily developed sensory adaptations of the protocerebrum, based perhaps on ancestral Anlagen that were vestigial in the first euarthropods (i.e. isoxyids and ‘megacheirans’). While there is no rationale to support the idea that both features were originally appendicular, it seems equally improbable that none of them bore any relationship to protocerebral organs present among lobopodians. This could be the case if the dinocaridid frontal appendages—being allegedly themselves protocerebral and homologous to onychophoran antennae—evolved into the ‘hypostome-labrum’ [62,84,85]. However, this hypothesis remains contradicted by all external morphological and anatomical evidence currently available, which shows a clear phylogenetic continuity of the frontalmost appendages between dinocaridids, isoxyids and megacheirans [9,69,86]. Instead, protocerebral organs elaborated during the evolution of the lobopodian grade must have been present in reduced form among dinocaridids, isoxyids and megacheirans, and later co-opted as sophisticated sensory organs during the early evolution of mandibulates, when they also served as the foundation from which the labrum originated. The lack of evidence for a fully formed crustacean-like labrum in hymenocarines as well as the known morphology of ‘epistome-labrum’ organs in chelicerates point to the ancestral morphology of this feature among euarthropods.

#### 5.2.5. Other cephalothoracic components

##### 5.2.5.1. Antennules

**Figure 6.**
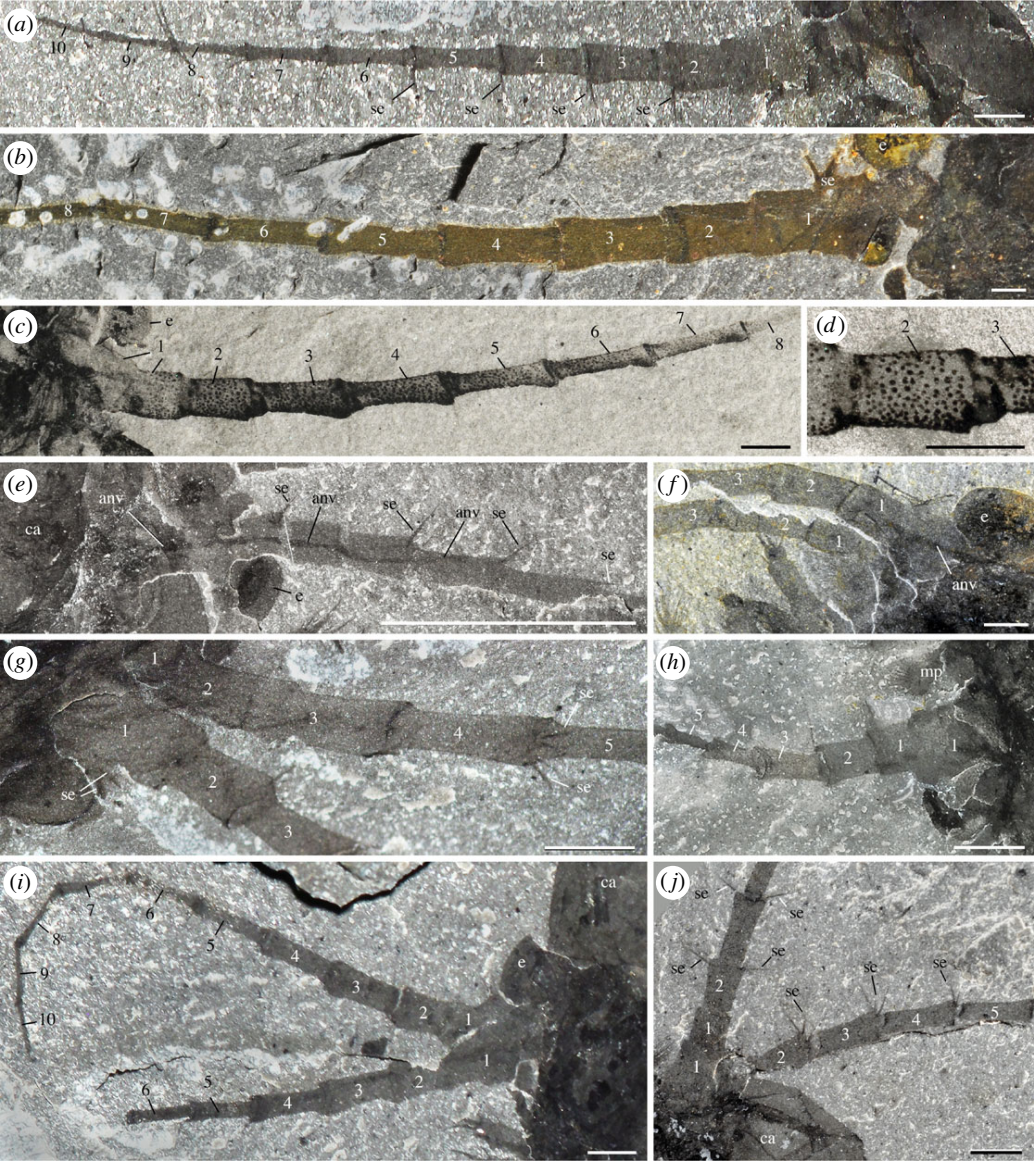
*Waptia fieldensis* Walcott, 1912 [10] from the middle Cambrian (Series 3, Stage 5) Burgess Shale, British Columbia, Canada; details of antennule. (*a*) ROMIP 64284, complete 10-segmented antennule. (*b*) USNM 511397 showing well-preserved basal podomere. (*c,d*) USNM 138231, general view and details of micro-ornament. (*e,f*) USNM 529135 and USNM 529189 showing antennal nerves. (*g–j*) ROMIP 64281, USNM 57682, ROMIP 64289, USNM 83948e, showing setae. All images are photographs taken under cross-polarized light. Abbreviations are as follows: anv, antennal nerve; ca, carapace; e, eye; mp, mandibular palp; se, setae; 1–10, antennular podomeres 1 to 10. Scale bars: 1 mm in *a–d, f–j*; 5 mm in *e*.

*Description.* The anteriormost appendages in *W. fieldensis* have ten elongated podomeres. The proximalmost podomere looks like a small turret mounted on a broad, roughly trapezoidal base (figures 5*a*–*d* and 6*b*,*g*,*h*). The basal part of this podomere bears a small protuberance from which two stout diverging spines and possibly additional setae are projecting, seemingly directed towards the sagittal plane of the body. The remaining nine podomeres form a relatively long and flexible antennular ramus, as long as or slightly longer than the carapace. The podomeres of the antennule are all cylindrical, elongated and splayed anteriorly, nesting within each other via a well-developed articulated joint marked by a reinforced cuticular rim (figure 5*c*). Their diameter decreases gradually towards the tip of the appendage. The mid-length diameter of the podomere to its length ratio, calculated from the best preserved appendages (figure 6), passes from 0.7 (segment 2) to 0.05 (podomere 10), the length of podomeres 3 to 10 being almost constant. The distal end of each podomere (i.e. around the anterior rim), except for the terminal one, is crowned by a radiating bundle of at least four stiff setae around 500–700 µm long (figure 6*g*). These setae stand at an angle of around 75° to 95° with the main axis of the appendage (e.g. figure 6*j*) and seem to have a basal articulation.

As for protocaridids [9], we interpret the anteriormost pair of appendages as antennules (=A1), owing to their uniramous and multisegmented morphology. We find no compelling argument to consider these appendages as antennae (=A2) behind an undeveloped pair of antennules.

Specimens observed in dorsal or ventral view indicate that the antennules have diverging axes and form a V-shaped structure of about 45–55° (eight specimens measured; e.g. figures 1*a*,*h* and 4*a*,*c*). In the majority of complete specimens preserved in lateral view (e.g. figure 5*a*) the antennules are pointing forwards and have a gently curved outline. None of them is recurved backwards along the carapace. This configuration does not seem to have resulted from compaction or post-mortem displacement but more probably indicates the natural orientation of antennules in life. The strong articulated joints (figures 5*c* and 6*d*) and slender shape of the antennules suggest that they were movable, flexible and robust.

**Figure 7.**
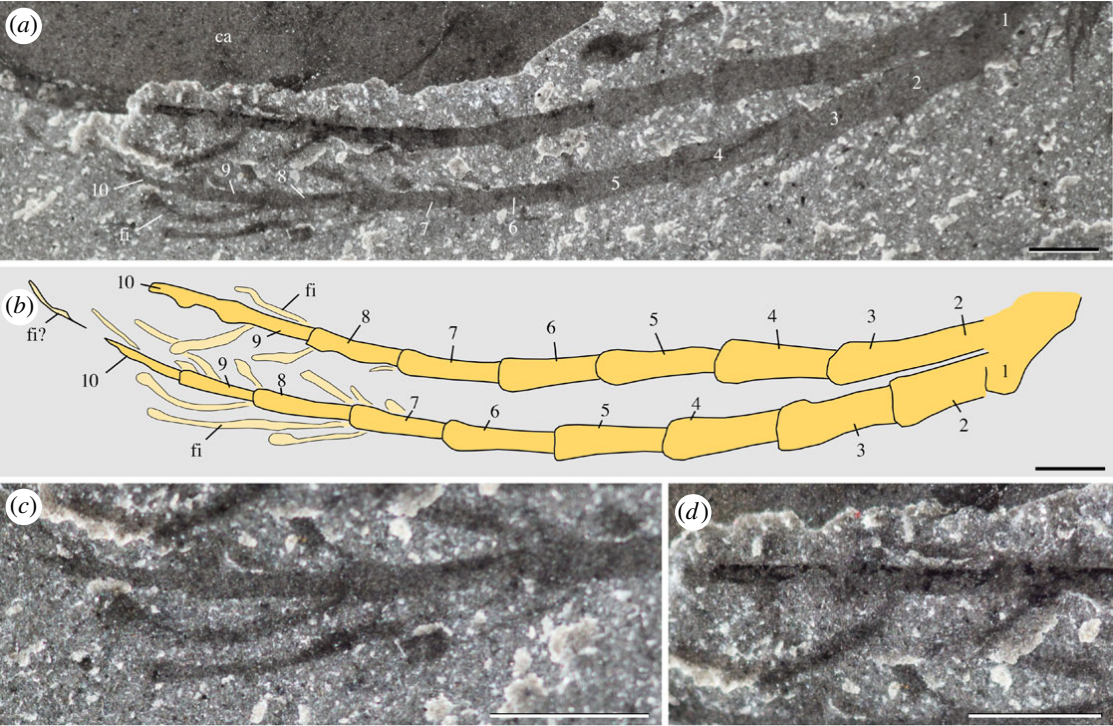
*Waptia fieldensis* Walcott, 1912 [10] from the middle Cambrian (Series 3, Stage 5) Burgess Shale, British Columbia, Canada; distal part of antennule. (*a–d*) ROMIP 56427, general view of both antennules and details of filament-like distal features (see also figure 1*i*). *a, c* and *d* are photographs taken under cross-polarized light. Abbreviations are as follows: ca, carapace; fi, filaments; 1–10, antennule podomeres 1 to 10. Scale bars: 1 mm.

The well-preserved antennules of USNM 138231 (figure 6*c*,*d*) display a dense concentration of evenly spaced tiny pustule-like cuticular features over the surface of podomeres (except on articular joints), but nowhere else on the cuticle of the animal. We interpret them as possible biological features such as microscales, cupules or hooded sensillae rather than preservational artefacts. A dark, sharply defined tract goes through the proximal part of several antennules (e.g. figure 6*e*). It is interpreted as the antennal nerve (see section on nervous system). ROMIP 56427 (figures 1*i* and 7) shows a pair of displaced antennules with filament-like structures attached to the most distal podomeres (7–10). Most of these structures seem to be inserted around the distal margin of podomeres similarly as the antennular setae but differ from them in their curved outline and apparent lobate termination (figure 7*c*,*d*). Their consistent distribution on both antennules might suggest that they are specialized terminal sensory filaments rather than ectoparasites randomly attached to the cuticle or undetermined organic fragments superimposed. The presence of these filaments has not been confirmed in any other specimens, ruling out a potential sexual dimorphism, and might represent preservation of a rare ecophenotype. We cannot completely rule out that they are not superimposed undetermined organic fragments, although their preservation style matches the antennules and no other similar structures have been observed in isolation.

*Discussion.* Strausfeld [15] argued that the antennules of *W. fieldensis* were provided with a complex set of sensory features such as short and long setae interpreted, respectively, as possible rows of chemosensory aesthetascs and long haptic sensilla with mechanosensory functions (e.g. [87,88]). We see no fossil evidence for two different types of setae in *W. fieldensis*. Only stiff and pointed setae seem to occur at the outer distal margin of the antennular podomeres (e.g. figure 6*j*). By their overall shape and insertion mode, these setae recall those regularly distributed along the antennae of extant crustaceans such as *Nebalia* (electronic supplementary material, S7j–m; [13,89], fig. 3]. We agree with Strausfeld [15] that they resemble the haptic sensilla of modern crustaceans [90]. However, they may have served multisensory functions (e.g. recognizing objects through touch and detecting chemical signals in water) as in many extant crustaceans. Aesthetascs have an olfactory function and typically occur along the antennules of extant crustaceans as bunches of relatively short filaments with blunt tips (e.g. *Nebalia*; electronic supplementary material, S7h). Although no traces of such closely packed filaments are found in *W. fieldensis*, aesthetascs may occur as more isolated filaments near the tips of antennae (ROMIP 56427; figures 1*i* and 7; see description above).

Strausfeld [13,15] also suggested that the antennules of *Waptia* could have borne hooded sensilla [91] although he did not clearly illustrate their presence. These tiny sensory features which are frequent in extant crustaceans might indeed be present in *W. fieldensis* by the regular punctuations on antennular podomeres described above (figure 6*c*,*d*).

##### 5.2.5.2. Mandibles

**Figure 8.**
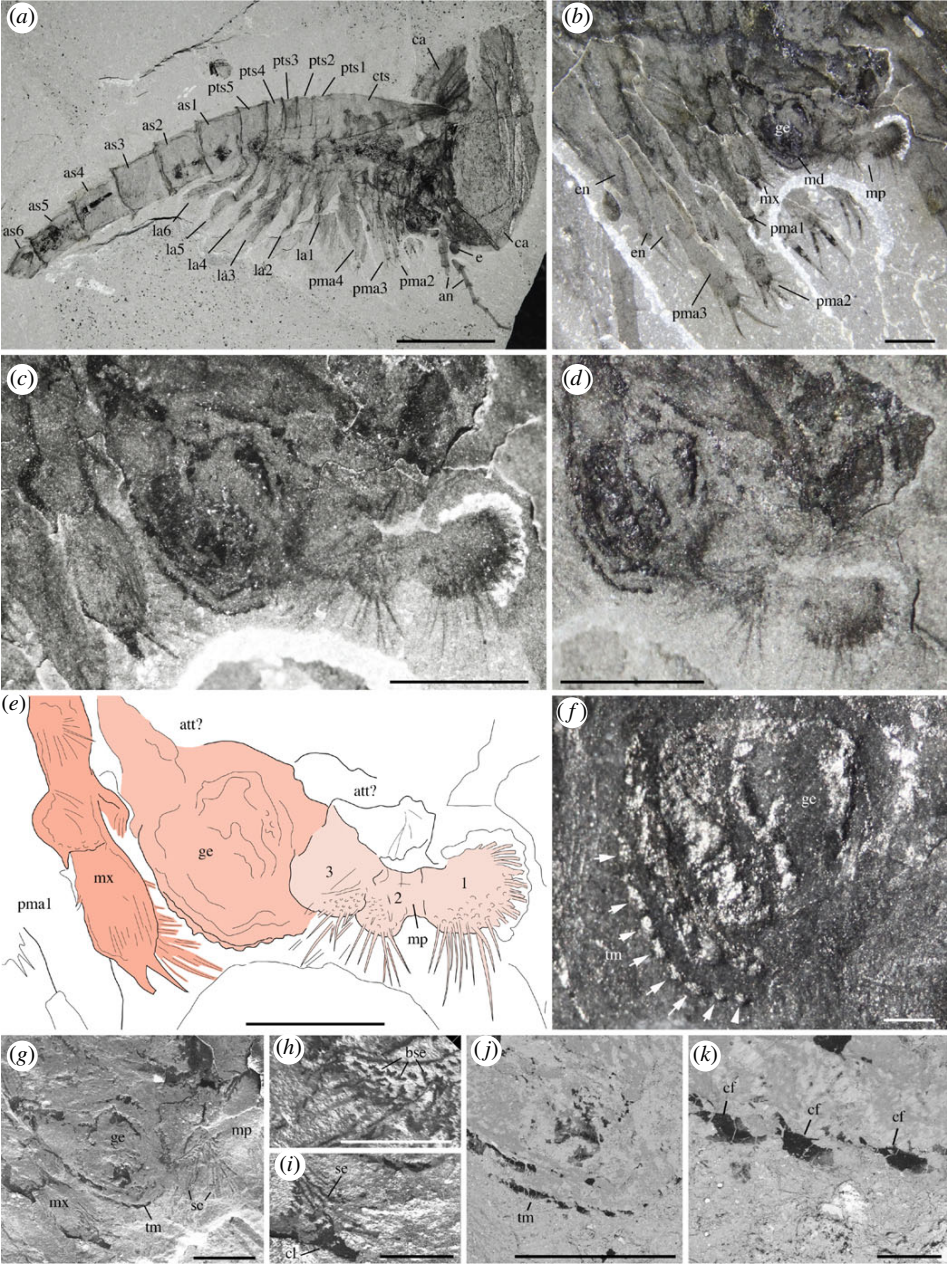
*Waptia fieldensis* Walcott, 1912 [10] from the middle Cambrian (Series 3, Stage 5) Burgess Shale, British Columbia, Canada; ROMIP 56432, mandibles and maxillules. (*a*) General view. (*b*) Detailed view of post-antennular segments and appendages. (*c–e*) Mandible and maxillule in lateral view. (*f*) Toothed margin of mandible (white arrows). (*g–k*) Mandible and maxillules. (*g*) General view. (*h*) Details of setae on mandibular palp. (*i*) Tip of maxillule. (*j,k*) Toothed margin of mandible, general view and details of carbonaceous film. *a–d* are photographs taken under cross-polarized light (*d*, under water); *g–k* are backscattered SEM images. Abbreviations are as follows: an, antennule; as1–6, 1st to 6th abdominal segments; att?, attachment of mandible to body; ca, carapace; cf, carbonaceous film; bse, base of setae; cl, claw; cts, cephalothoracic segments; e, eye; en, endite; la1–6, 1st to 6th lamellate post-cephalothoracic segments; ge, gnathal element of mandible; md, mandible; mp, mandibular palp; mx, maxillule; pma1–4, 1st to 4th post-maxillular cephalothoracic appendages; pts1–5, 1st to 5th post-cephalothoracic segments; se; setae; tm, toothed margin; 1–3, 1st to 3rd podomere of mandibular palp. Scale bars: 1 cm in *a*; 5 mm in *b–e*; 1 mm in *f, g, j*; 500 µm in *h, i*; 100 µm in *k*.

**Figure 9.**
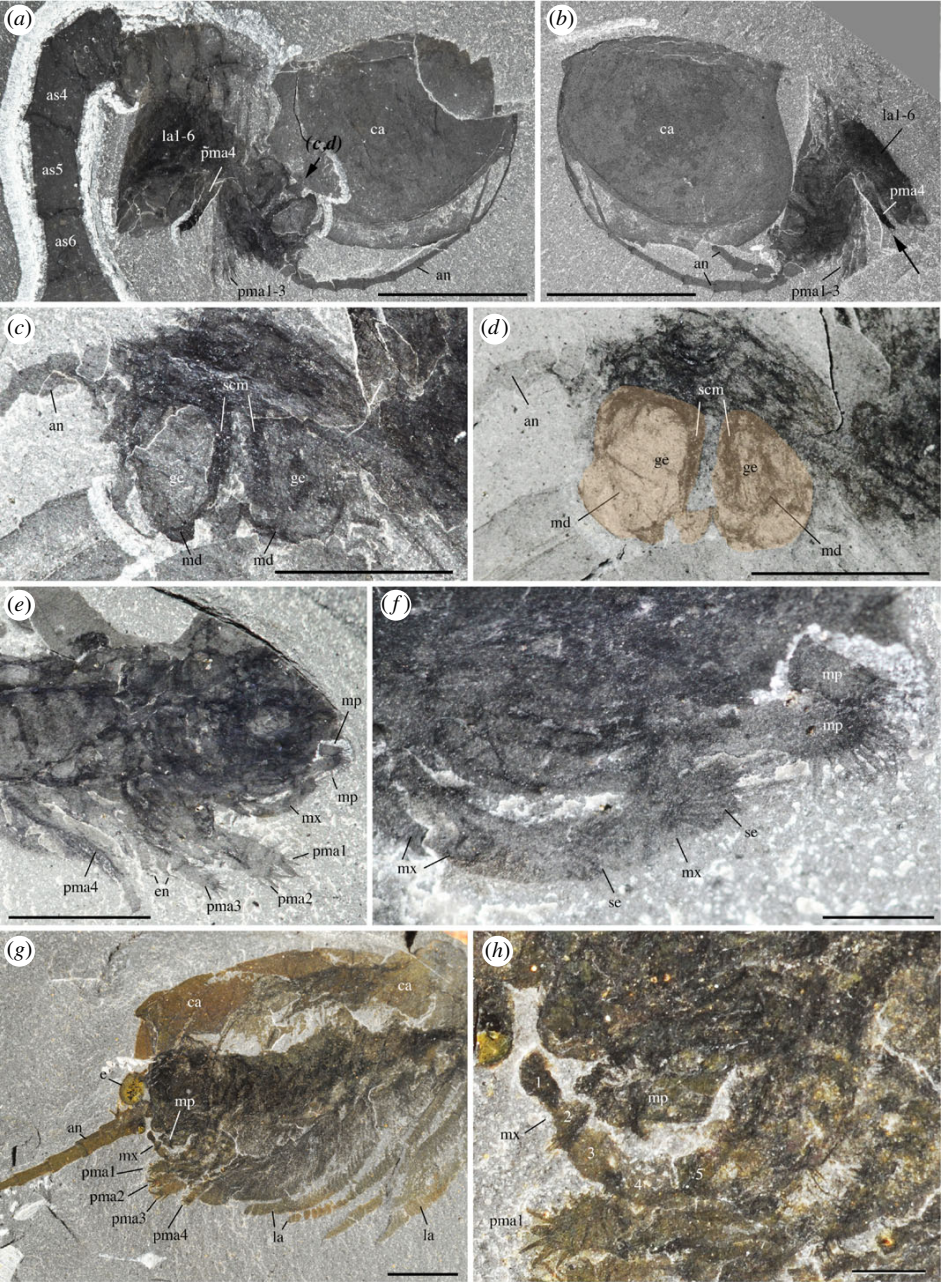
*Waptia fieldensis* Walcott, 1912 [10] from the middle Cambrian (Series 3, Stage 5) Burgess Shale, British Columbia, Canada; mandibles and maxillules. (*a–d*) ROMIP 64294; disarticulated specimen; general views of part and counterpart and details of mandibles in light orange (in *d*). (*e,f*) ROMIP 64578; general view of cephalic appendages and details of mandibular palps and maxillule. (*g,h*) USNM 511397; general view of cephalothoracic appendages and details of maxillule. All images are photographs taken under cross-polarized light (*d*, under water). Abbreviations are as follows: an, antennule; as1–6, 1st to 6th abdominal segments; ca, carapace; en, endite; ge, gnathal element of mandible; la1–6, 1st to 6th lamellate post-cephalothoracic appendages; md, mandible; mp, mandibular palp; mx, maxillule; pma1–4, 1st to 4th post-maxillular cephalothoracic appendages; scm, sclerotized margin; se; setae; 1–5, 1st to 5th podomere of maxillule. Scale bars: 1 cm in *a,b*; 5 mm in *c–e, g*; 1 mm in *f,h*.

**Figure 10.**
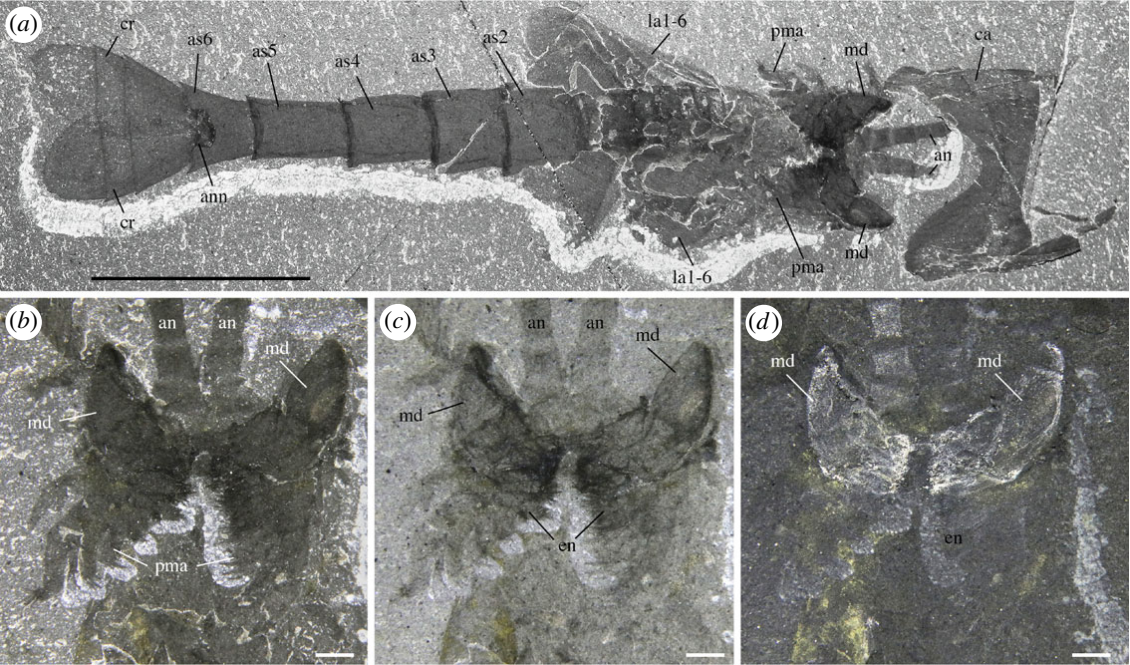
*Waptia fieldensis* Walcott, 1912 from the middle Cambrian (Series 3, Stage 5) Burgess Shale, British Columbia, Canada; anterior appendages in ventral view. (*a–d*) ROMIP 64285, slightly disarticulated specimen, general view and details of mandibles; the dark symmetrical feature most probably results from the overlap of the mandibles and the post-mandibular cephalothoracic appendages bearing endites. Photographs: dry specimen and polarized light in *a, b*; wet specimen and polarized light in *c*; wet specimen and direct light in *d*. Abbreviations are as follows: an, antennule; ann, anal notch; as1–6: 1st to 6th abdominal segments; ca, carapace; cr, caudal ramus; en, endite; la1–6, 1st to 6th lamellate post-cephalothoracic appendages; md, mandible; pma, post-maxillular cephalothoracic appendage. Scale bars: 1 cm in *a,b*; 1 mm in *c,d*.

*Description.* The appendage posterior and adjacent to the antennule is an enlarged, rounded element bearing a three-segmented setose projection (figures 8–10; electronic supplementary material, S11). The mesial margin of this element is lined with an undulated cuticular thickening often preserved in three dimensions (figure 8*f*) and underlined by carbonaceous films (figure 8*j*,*k*). A disarticulated specimen (ROMIP 64294; figure 9*a*,*d*) clearly shows that the right and left mandibles converge ventrally and symmetrically towards the sagittal plane (figure 9*c*,*d*) with their strongly sclerotized margins opposing each other. A comparable ventral convergence is observed in ROMIP 64285 (figure 10) but the outline of the mandibles is partly concealed by the overlapping post-maxillular cephalothoracic appendages.

The three-segmented projection bears numerous setae (figure 8*c*–*k*; electronic supplementary material, S11). In lateral view, the most distal podomere a rounded shape, is fringed with numerous stout radiating setae (figures 8*c*,*d* and 9*e*,*f*) and often extends beyond the anterior margin of the carapace (electronic supplementary material, S11). These setae (up to about 3 mm long; figure 7*e*) are not distributed within a single plane and form a relatively closely spaced comb-like structure (electronic supplementary material, S11). The distalmost segment is followed by a smaller podomere showing at least setation along the ventral surface (figure 8*c*,*d*). The third podomere is about the same size as the most distal one and also bears ventrally directed setae (figure 8*c*–*e*,*g*–*i*). The attachment of the appendage to the body seems to be relatively long but its exact proximalmost morphology remains uncertain.

*Discussion.* This appendage strongly resembles the mandible of extant crustaceans (e.g. isopods; electronic supplementary material, S10a,b) and is interpreted as such. Mandibles are modified appendages generally characterizing Mandibulata (Hexapoda, Myriapoda and Crustacea) and derived from a coxal (pre-basal) podomore. They consist of an enlarged basal gnathal element which bears a segmented palp (endopod) involved in sweeping and manipulating food particles.

Detailed comparative studies [92,93] show that the subdivision of the gnathal edge of extant Mandibulata into an incisor (pars incisivus) and a molar process (pars molaris) is an important homologous structure of the group, although mandibles display considerable morphological variation and specialization exemplified by their gnathal microscopic features (e.g. [92,93]).

Similar to protocaridids [9], the gnathal element of the mandible in *W. fieldensis* is simply round and seems to bear only one undivided masticatory margin (gnathal edge), as opposed to the majority of mandibulates that possess two. In that respect, such mandibles may be compared to those of branchiopods (e.g. [93]) such as *Triops* (Notostraca) and *Lynceu*s (Laevicaudata), which similarly bear a small number (8–13) of regularly distributed hump-like teeth (compare figure 8*f* and figs 7A-D, 8A-D in [93]). Several types of mandibles have been identified in middle and late Cambrian small carbonaceous fossil (SCF) assemblages that display remarkable resemblances to those of extant branchiopods, copepods and ostracods [94]. The mandibles of *Waptia* are easily distinguished from them by their sheer size (about 150–300 µm versus 5–7 mm), teeth (in the case of copepod-like mandibles), palp morphology and (apparent) lack of setose or textural ornamentation ([95], fig. 1); in that, SCF assemblages seem more derived and thus closer to extant forms, but this likeness may be driven by the differences in the mode of preservation. The mandibles of *W. fieldensis* most probably had a biting and grinding function as suggested by their toothed margins and converging ventral arrangement.

Strausfeld [15] reconstructed the mouth parts of *Waptia* as consisting of a set of three pairs of appendages inserted between the antennules and the first pair of leg-like appendages, and identified them as mandibles, first (Mx1) and second maxillae (Mx2). We reinvestigated the specimens on which Strausfeld's reconstructions are based and incorporated data from new fossil material (e.g. figures 8 and 9). Although we also identified a maxillule (Mx2; see §5.2.6), the alleged Mx1 ([15], fig. 2) likely represents the gnathal part of the mandible and therefore does not constitute a distinct appendage. Similarly, the alleged Mx2 [15] (figure 5*f,g*; USNM 83948e) clearly has a setose distal article which probably corresponds to the distal podomere of the mandibular palp (figure 8*c*,*d*).

##### 5.2.5.3. Maxillules

*Description.* A differentiated, elongate appendage is located between the mandible and the first pair of enditic limbs (figures 8*c*,*e* and 9*e*–*h*; electronic supplementary material, S11). By virtue of general terminology across mandibulate arthropods, this appendage is the maxillule (or first maxilla). The distalmost podomere has convex margins bearing many fine setae and is reminiscent of the distalmost podomere of the palp, but bears a pair of strong claws (figure 8*c*). The maxillule is stenopodous and is clearly composed of more than five podomeres (figure 9*g*,*h*). The total number of podomeres may be estimated to nine. However, podomere boundaries in the proximal part of the maxillule are not clearly defined. Bunches of setae occur on the second and third podomeres (figures 8*c*,*e* and 9*f*). The maxillule selectively preserves in lateral specimens which also show the mandibular palps, but not in frontal or latero-frontal specimens showing the tips of the post-maxillular cephalothoracic limbs and part of the gnathal edges of the mandibles. This suggests that the maxillules are inserted in a more mesial location (behind the mandibles) than the succeeding four pairs of limbs and that their tips also converged mesially, rather than spreading laterally. The maxillules were therefore probably assisting food manipulation during the action of the mandibles, as opposed to performing a raptorial or chewing function.

##### 5.2.5.4. Post-maxillular cephalothoracic appendages

**Figure 11.**
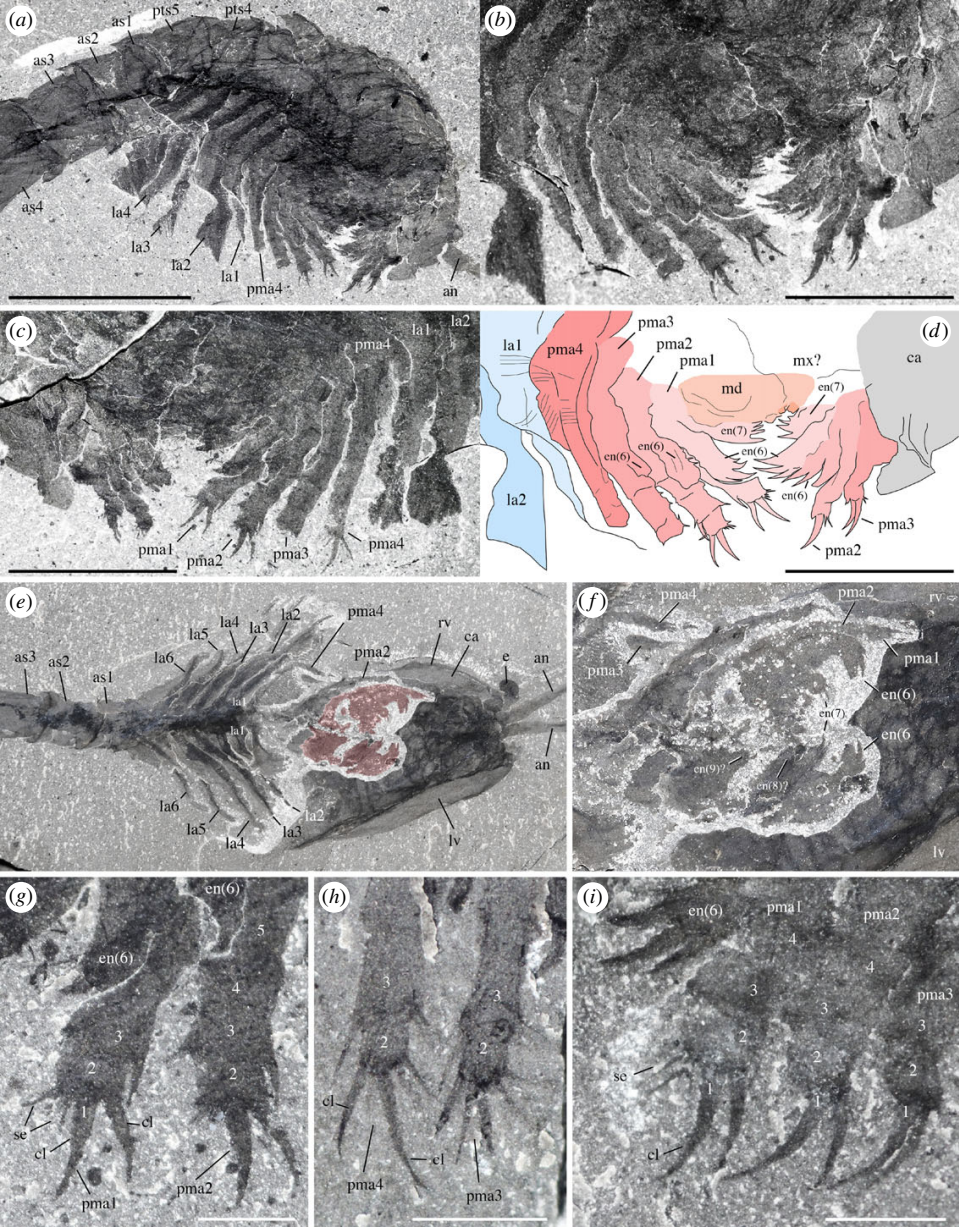
*Waptia fieldensis* Walcott, 1912 [10] from the middle Cambrian (Series 3, Stage 5) Burgess Shale, British Columbia, Canada; post-maxillular cephalothoracic appendages. (*a–d*) ROMIP 64291 showing post-mandibular cephalothoracic appendages in an intermediate frontal view; general view, details, counterpart and drawing (mandible and maxillule in light orange, post-maxillular appendages in light red and lamellate appendages in light blue). (*e,f*) ROMIP 64580 in ventral view showing post-maxillular cephalothoracic and post-cephalothoracic lamellate appendages; general view (first post-maxillular appendages in light red) and details. (*g–i*) Close-ups of distal ends of post-maxillular cephalothoracic appendages and endites, showing strong recurved claws and setae; ROMIP 64291 counterpart (*g*), ROMIP 56432 (*h*), ROMIP 64290 part (*i*), respectively, showing tips of post-maxillular cephalothoracic appendages and endites. All images are photographs taken under cross-polarized light. Abbreviations are as follows: an, antennule; as1–4, 1st to 4th abdominal segments; ca, carapace; cl, claw; e, eye; en(6)-en(9), endite of 6th to 9th podomere of post-maxillular cephalothoracic appendages; la1–6, 1st to 6th post-cephalothoracic lamellate appendages; lv; left valve; md, mandible; mx, maxillule; pma1–4, 1st to 4th post-maxillular cephalothoracic appendages; pts, post-cephalothoracic segment; rv, right valve; se; setae; 1–5, 1st to 5th podomeres of post-maxillular cephalothoracic appendages. Scale bars: 1 cm in *a*; 5 mm in *b–f*; 1 mm in *g–i*.

*Description.* A series of four uniramous leg-like appendages are inserted posterior to the maxillules (figure 11*a*,*c*,*e*; electronic supplementary material, S12 and S13). The first three pairs are entirely segmented, while only the termination is stenopodous in the fourth. We homologize the five-segmented distal termination of the fourth pair with the equivalent five-segmented distal podomeres of the first three pairs, which we interpret as the endopod. The remaining basal part of the first three pairs, the basipod (or basis), consists of at least four segments characterized by the presence of well-developed endites, which are stronger on the first pair, in particular the distalmost endites, inserting along their inner margin. The basipod of the fourth pair has a very different structure, being annulated and fringed with lamellae, thus resembling the post-cephalothoracic lamellate appendages (see §5.2.6).

**Figure 12.**
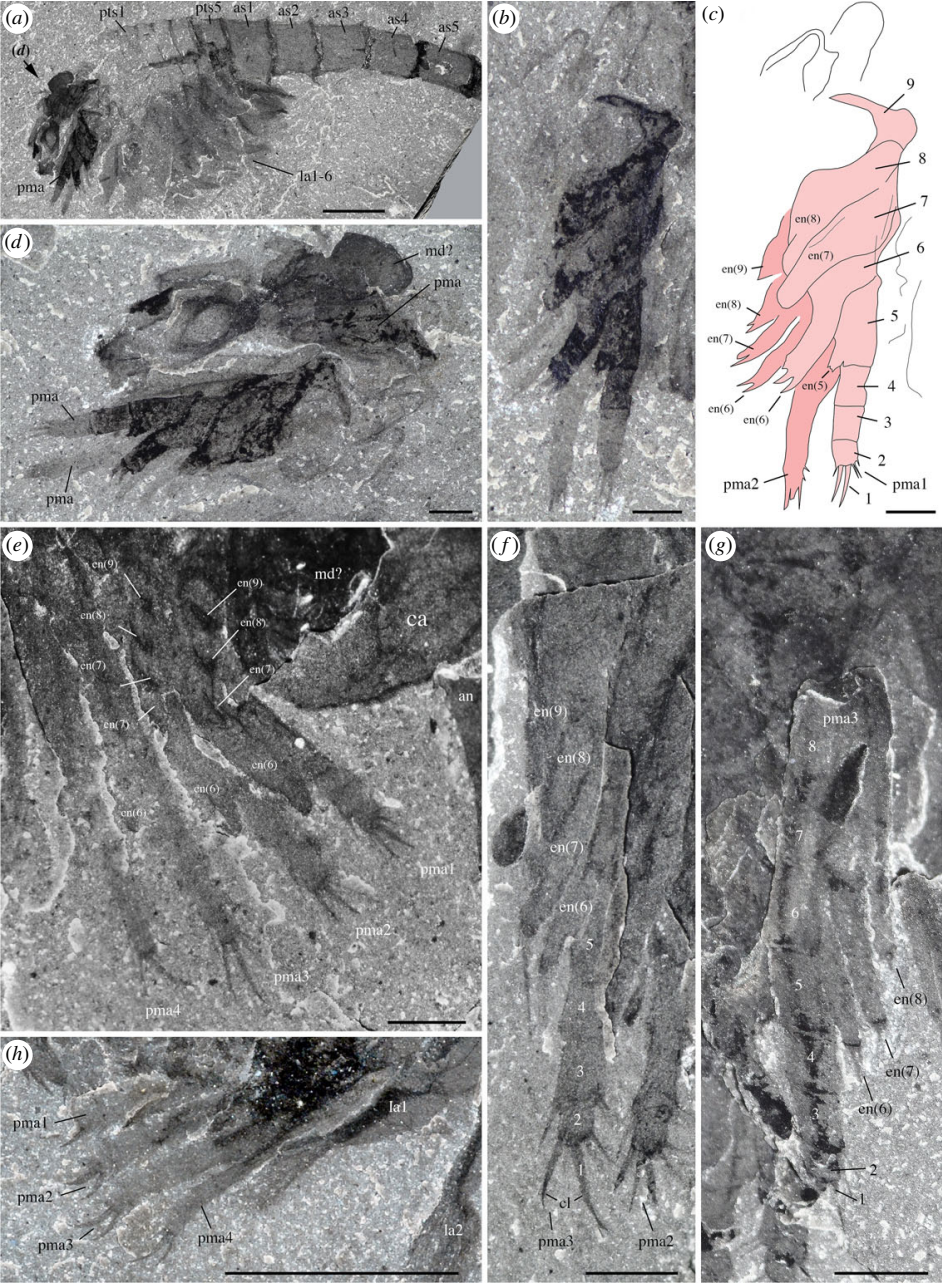
*Waptia fieldensis* Walcott, 1912 [10] from the middle Cambrian (Series 3, Stage 5) Burgess Shale, British Columbia, Canada; details of post-maxillular cephalothoracic appendages. (*a–d*) ROMIP 64292; general view and details of detached appendages, counterpart showing two adjacent appendages (presumably 1st and 2nd pairs) bearing endites. (*e*) ROMIP 56947, 1st to 4th appendages in ventral view. (*f*) ROMIP 56432, 3rd appendage with endites. (*g*) ROMIP 64288, 3rd appendage with endites. (*h*) USNM 139214, 1st to 4th appendages in lateral view. All images are photographs taken under cross-polarized light. Abbreviations are as follows: as1–5, 1st to 5th abdominal segments; cl, claw; en(5)-en(9), endite of 5th to 9th podomere of post-maxillular cephalothoracic appendages; la1–6, 1st to 6th lamellate post-cephalothoracic appendages; md, mandible; pma1–4, 1st to 4th post-maxillular cephalothoracic appendages; pts1–5, 1st to 5th post-cephalothoracic segment; 1–9, 1st to 9th podomere of post-maxillular cephalothoracic appendages. Scale bars: 5 mm in *a, h*; 1 mm in *b–g*.

**Figure 13.**
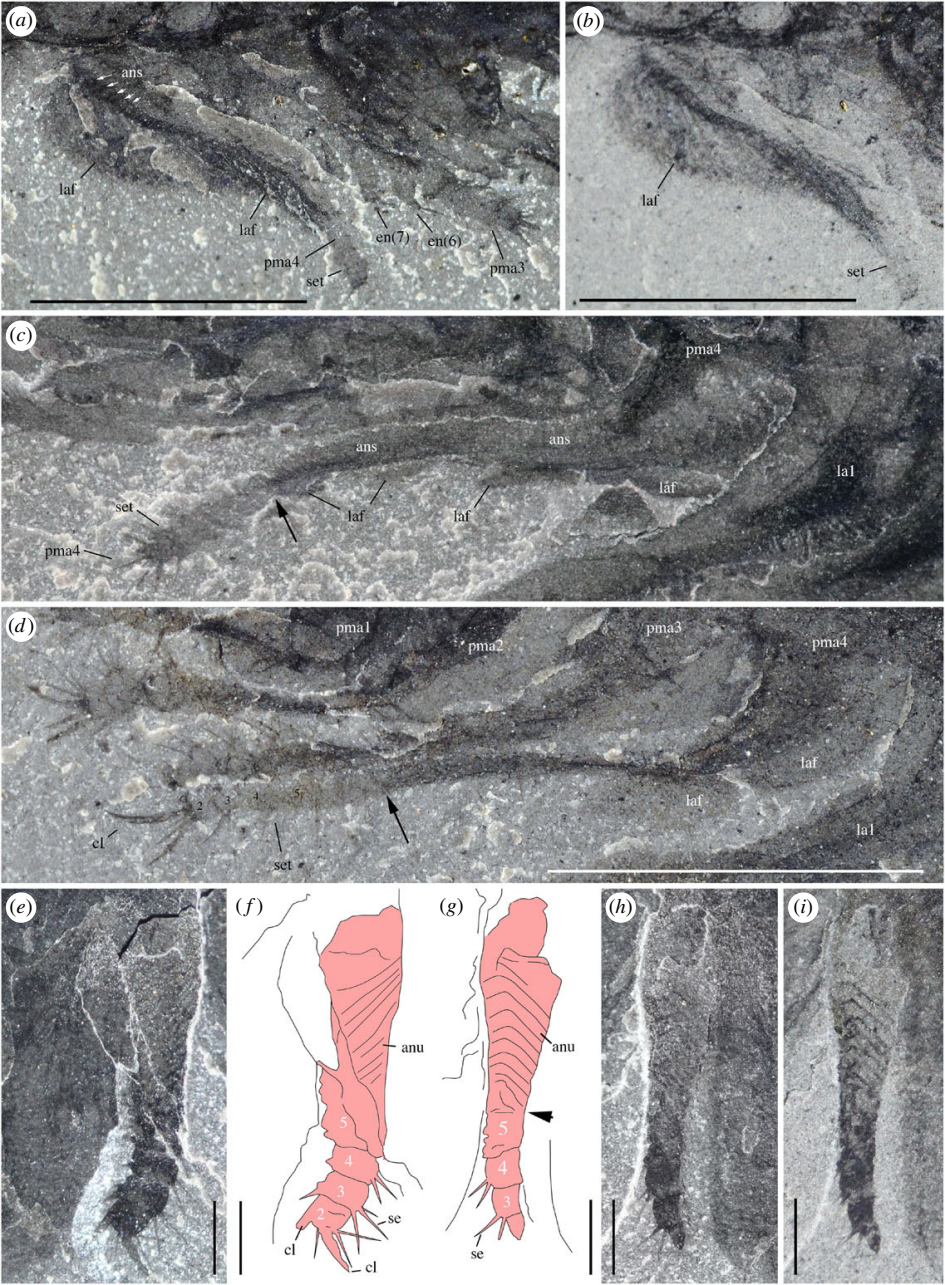
*Waptia fieldensis* Walcott, 1912 [10] from the middle Cambrian (Series 3, Stage 5) Burgess Shale, British Columbia, Canada; fourth post-maxillular cephalothoracic appendage. (*a,b*) ROMIP 64578**,** lateral view showing 3rd and 4th post-maxillular cephalothoracic appendages; small white arrows indicate annulations (*c*) ROMIP 64580, lateral view; black arrow indicates base of lamellate structure. (*d*) USNM 83948e, lateral view showing 1st to 4th appendages. (*e–i*) ROMIP 64294, distal part of 4th appendage, part (*e,f*) and counterpart (*g–i*) (see location in figure 9*b*); black arrow indicates the base of the annulated part. All images are photographs taken under cross-polarized light (*b*,*i* under water). Abbreviations are as follows: ans, annulated stem; anu, annulation; cl, claw; en(6) and en(7), endite of 6th and 7th podomere; laf, lamellate fringe; la1, 1st lamellate post-cephalothoracic appendage; pma1–4, 1st to 4th post-maxillular cephalothoracic appendages; se, setae; set, segmented tip; 1–5, 1st to 5th podomere of the 4th post-maxillular cephalothoracic appendage. Scale bars: 5 mm in *a–d*; 1 mm in *e–i*.

In lateral view the post-maxillular post-cephalothoracic appendages (pma) have a slender, elongate shape slightly protruding beyond the anteroventral margin of the carapace (figure 1*c*,*e*), with their axes directed forwards and lying obliquely or sub-horizontally (figure 1*b*,*f*; electronic supplementary material, S12a,c). Two adjacent appendages (presumably pma1 and pma2), detached from the body (ROMIP 64292, figure 12*a*–*d*) and preserved in mesial view, exhibit a series of four exceptionally well-developed endites projecting obliquely downwards along the basipod inner margin. Their five segmented (1–5), almost cylindrical endopod contrasts markedly with their much thicker basipod made up of at least four, possibly five podomeres (6–9). Uncertainties remain concerning the proximalmost segmented part of these two isolated appendages and their attachment to the body. The same overall appendage and enditic structure is observed, completely or incompletely, in numerous other specimens (figures 11*a*–*f* and 12*e*–*h*; electronic supplementary material, S12d,e and S13). Podomere 1 is a pair of long articulated claws flanked with at least three stiff radiating setae of various sizes, but thinner and shorter than the claws, with a straight or slightly curved outline (figures 11*g*–*i* and 12*e*; electronic supplementary material, S12d). Podomere 2 is the shortest one and has a sub-quadrate outline. Podomeres 3–5 share the same overall cylindrical shape but increase gradually in size towards the basipod. The inner and outer corners of their distal margins bear a bunch of at least three radiating setae and a thicker seta (or cuticular spiny projection as in podomere 3; figure 11*g*), respectively. In ROMIP 64292, the inner distal margin of podomere 5 on pma1 extends into a short, pointed endite-like structure (figure 12*b*,*c*). However, this morphological trait was not identified in other specimens, presumably due to its small size and the lack of other disarticulated limbs where this feature is likely to be evident. pma1 has a remarkably stout, slightly curved endite on its sixth podomere, terminated by three pointed claws and a smaller outer spine (figure 11*b*,*d*). A similar but smaller projection occurs on podomere 7. Podomeres 8 and 9 have comparable but more reduced pointed endites with uncertain morphology. pma2 and pma3 are both characterized by longer, straighter and thinner endites terminated by a short, bifurcated claw-like feature which seems to have a basal articulation (figure 12*f*,*g*). pma4 lacks endites (figure 13), its basipod being evenly annulated (figure 13*e*–*i*) and fringed with relatively short lamellae similar to those of the lamellate appendages (figure 13*a*,*b*) and gradually decreasing in size towards the base of the endopod.

Several specimens preserved obliquely relative to the bedding or with their inner parts displaced shows that pma1–4 converge symmetrically towards the sagittal plane of the animal with the pointed tips of their endites facing each other (figure 11*a*–*d*; electronic supplementary material, S13). This configuration suggests a function related to food processing.

*Discussion.* Although we lack detailed information concerning the proximalmost morphology of pma1–3 and their attachment to the body, it is clear that the basipod of these appendages comprises a succession of well-delimited enditic podomeres as in protocaridids [9]. In *W. fieldensis*, they are characteristically very long. The morphological boundary between the endopod and the basipod, as in these taxa, is marked by the development of endites on basipod podomeres. However, the endopods of *W. fieldensis* have only five podomeres instead of the seven in protocaridids, because a distinction based here on the presence of prominent endites clearly isolates a five-segmented endopod from the basis. This interpretation provides *W. fieldensis* with a typically pancrustacean groundplan endopod composed of (at most) five podomeres [95].

Strausfeld [15] recognized five pairs of anterior ‘walking legs’ in *W. fieldensis* and described them as being biramous with rows of setae along the endopod podomeres and two types of short exopods (three-segmented ones on appendages 1 and 2 and lamellate, arbelos-shaped on appendages 3–5). This interpretation is not confirmed by our observations. The number of post-maxillular cephalothoracic appendages is unquestionably four in *W. fieldensis* (e.g. figures 11 and 12). Their prominent endites seem to have been misinterpreted as exopods by Strausfeld [15]. We see no evidence of additional marginal rows of setae and of more than two claws per appendage. Our study also reveals that the post-maxillular appendages are not primarily ‘walking legs’ but rather play a more important role in feeding (e.g. opposing endites for manipulating and macerating food). A role as ‘walking legs’ is unlikely, *W. fieldensis* being essentially a nektobenthic swimmer (see below).

#### 5.2.6. Post-cephalothoracic tagma

**Figure 14.**
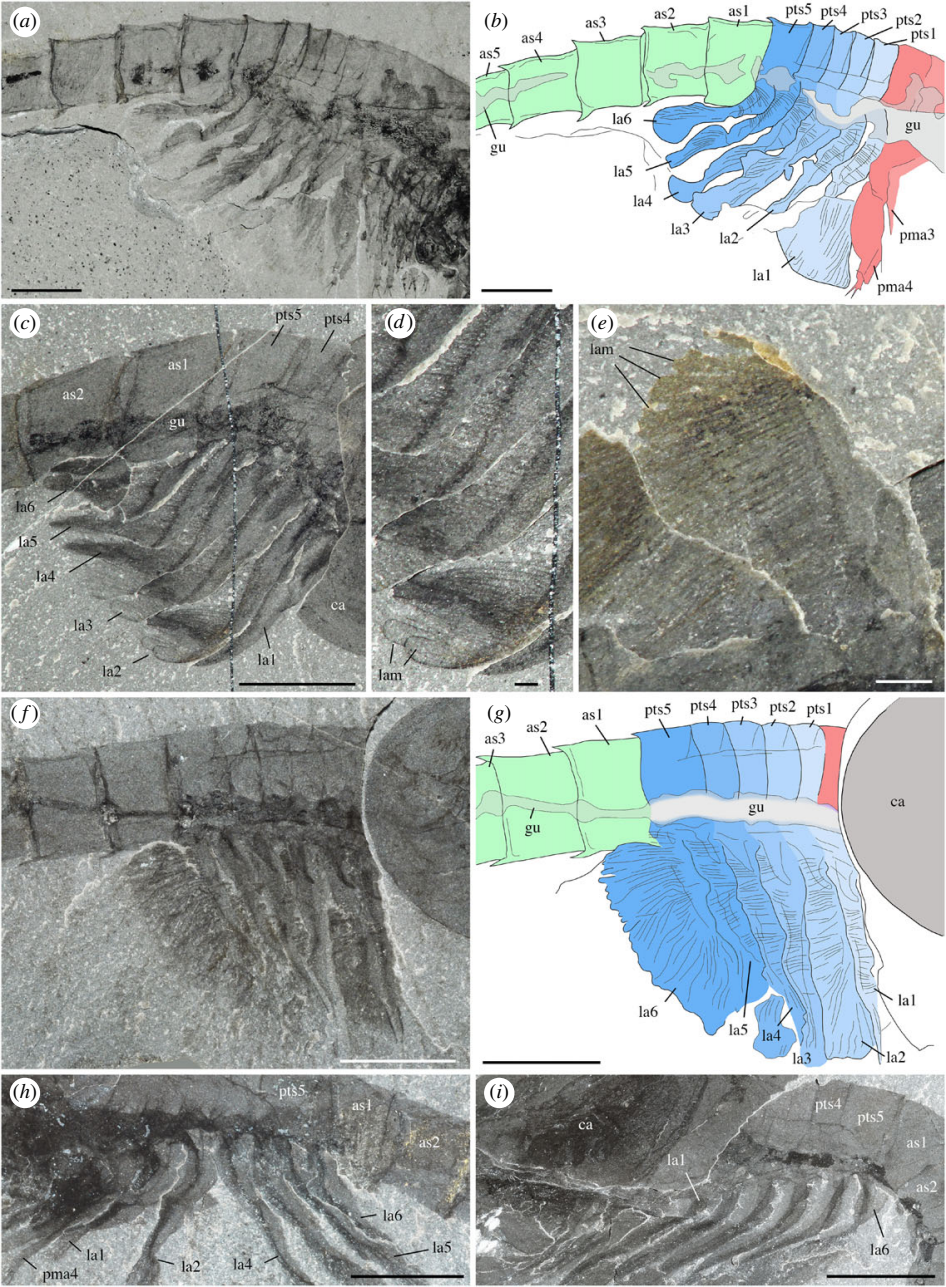
*Waptia fieldensis* Walcott, 1912 [10] from the middle Cambrian (Series 3, Stage 5) Burgess Shale, British Columbia, Canada; post-cephalothoracic lamellate appendages. (*a,b*) ROMIP 56432, lateral view. (*c,d*) ROMIP 56421, general view and details of lamellar structure. (*e*) ROMIP 64293, first post-cephalothoracic lamellate appendage showing details of stem and lamellae. (*f,g*) USNM 83949, lateral view. (*h,i*) USNM 139214 and USNM 268270, respectively; lateral view and proximal part of post-cephalothoracic lamellate appendages. All images are photographs taken under cross-polarized light. Lamellate appendages and corresponding post-cephalothoracic segments in light blue, abdominal segments in light green and post-maxillular cephalothoracic segments in light red. Abbreviations are as follows: as1–5, 1st to 5th abdominal segments; ca, carapace; gu, gut; la1–6, 1st to 6th lamellate post-cephalothoracic appendages; lam, lamella; pma3 and pma4, 3rd and 4th post-maxillular cephalothoracic appendages; pts1–5, 1st to 5th post-cephalothoracic segments. Scale bars: 5 mm in *a–c,f–i*; 1 mm in *d,e*.

**Figure 15.**
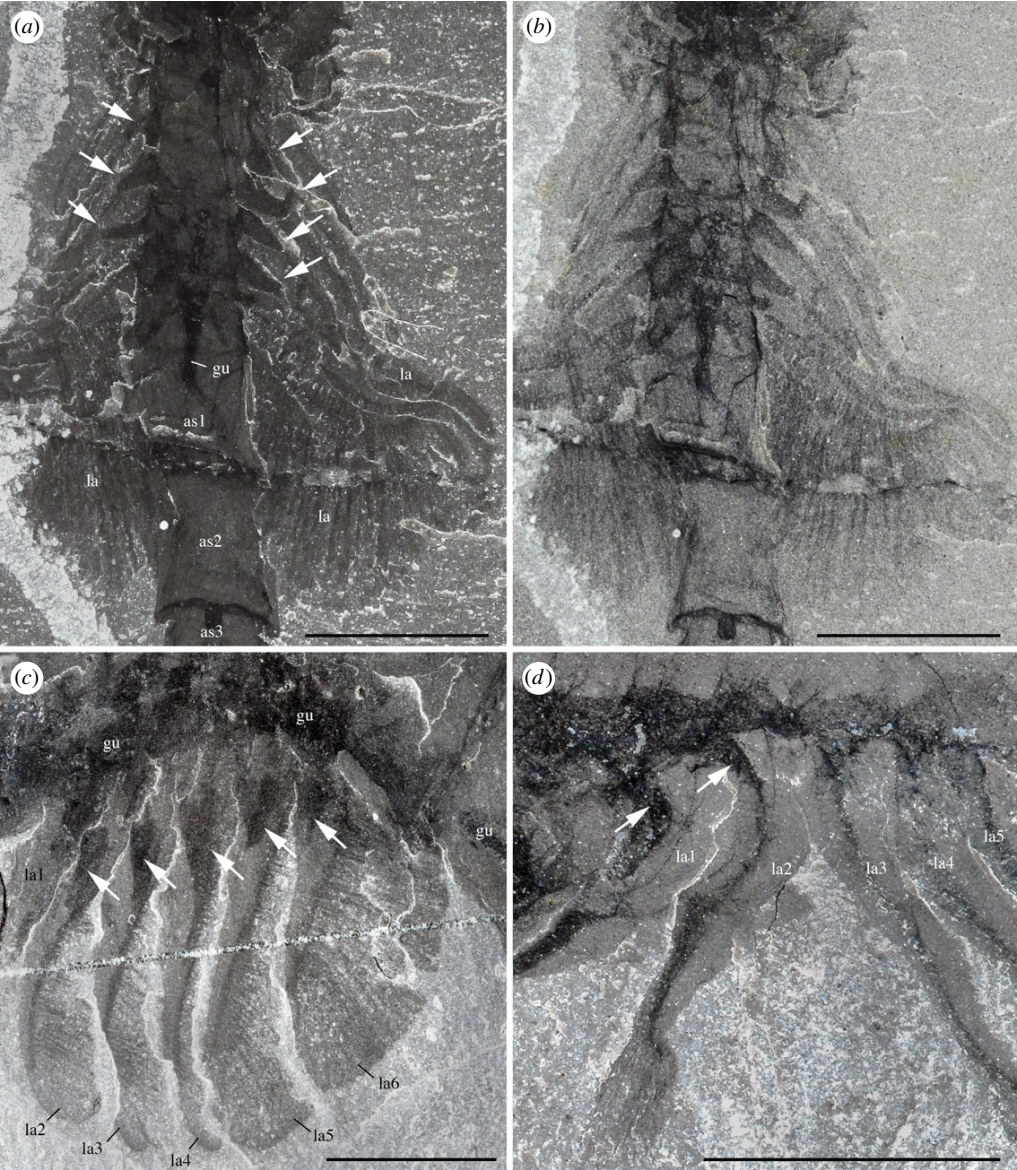
*Waptia fieldensis* Walcott, 1912 [10] from the middle Cambrian (Series 3, Stage 5) Burgess Shale, British Columbia, Canada; lamellate appendages. (*a,b*) ROMIP 64295, ventral view showing gut and triangular dark features (white arrows) within the proximal part of the lamellate appendages (overall views and elemental maps, figure 2). (*c*) USNM 529197. (*d*) USNM 139214 (overall views, figure 1*b* and electronic supplementary material, S13a,c). All images are photographs taken under cross-polarized light (*b* in water). Abbreviations are as follows: ca, carapace; gu, gut; la1–6, 1st to 6th lamellate post-cephalothoracic appendages; as1–5, 1st to 5th abdominal segments. Scale bars: 5 mm.

**Figure 16.**
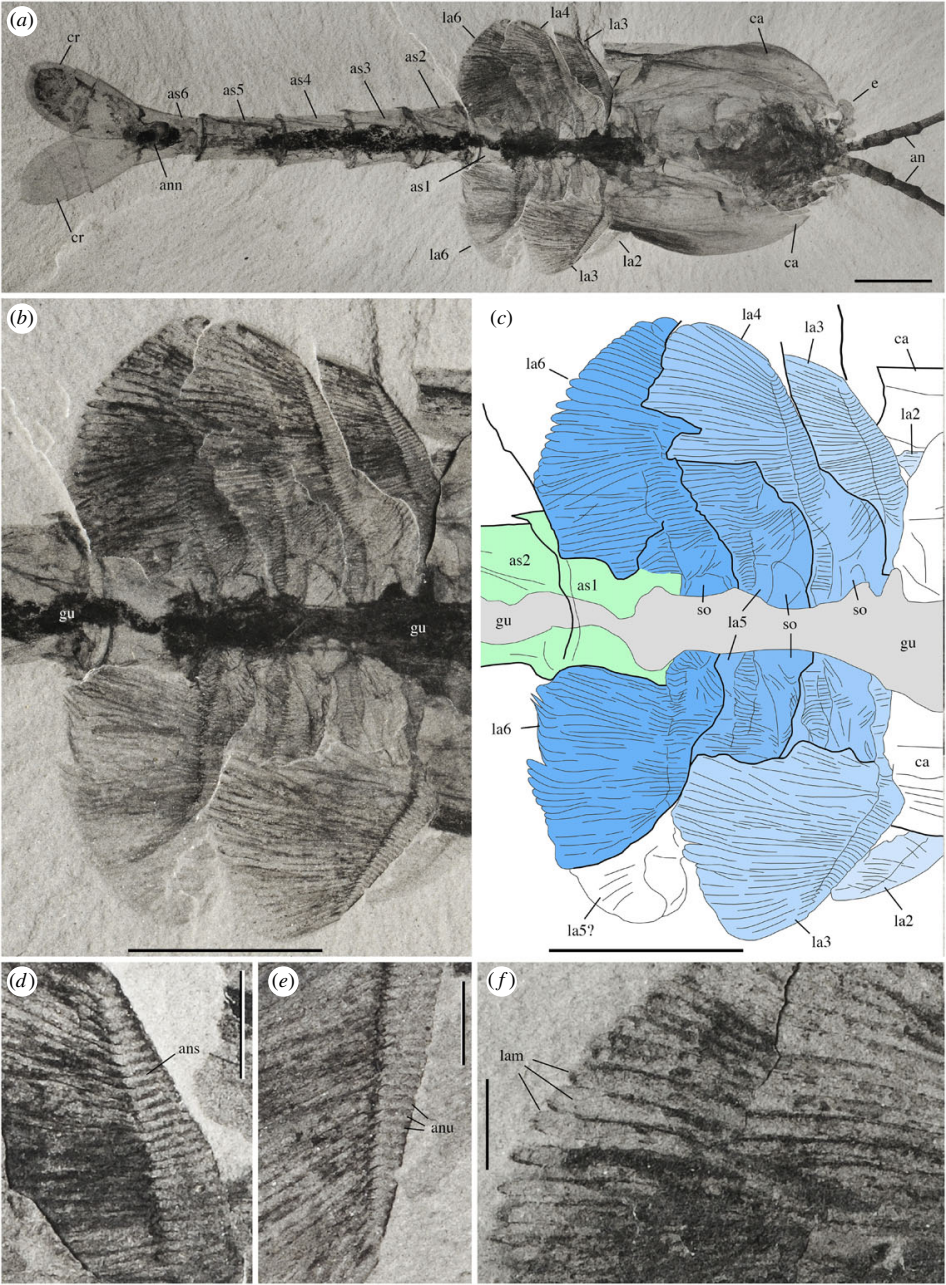
*Waptia fieldensis* Walcott, 1912 [10] from the middle Cambrian (Series 3, Stage 5) Burgess Shale, British Columbia, Canada; lamellate post-cephalothoracic appendages. (*a–f*) USNM 275504. (*a*) General ventral view. (*b,c*) Details of appendages. (*d–f*) Details of annulated stem and lamellae. All images are photographs taken under cross-polarized light. Lamellate appendages and corresponding post-cephalothoracic segments in light blue and abdominal segments in light green. Abbreviations are as follows: an, antennule; ann, anal notch; anu, annulation; as1–6, 1st to 6th abdominal segment; ans, annulated stem; ca, carapace; cr, caudal ramus; e, eye; gu, gut; la1–6, 1st to 6th lamellate post-cephalothoracic appendages; lam: lamella; so, socket. Scale bars: 5 mm in *a–c*; 1 mm in *d–f*.

**Figure 17.**
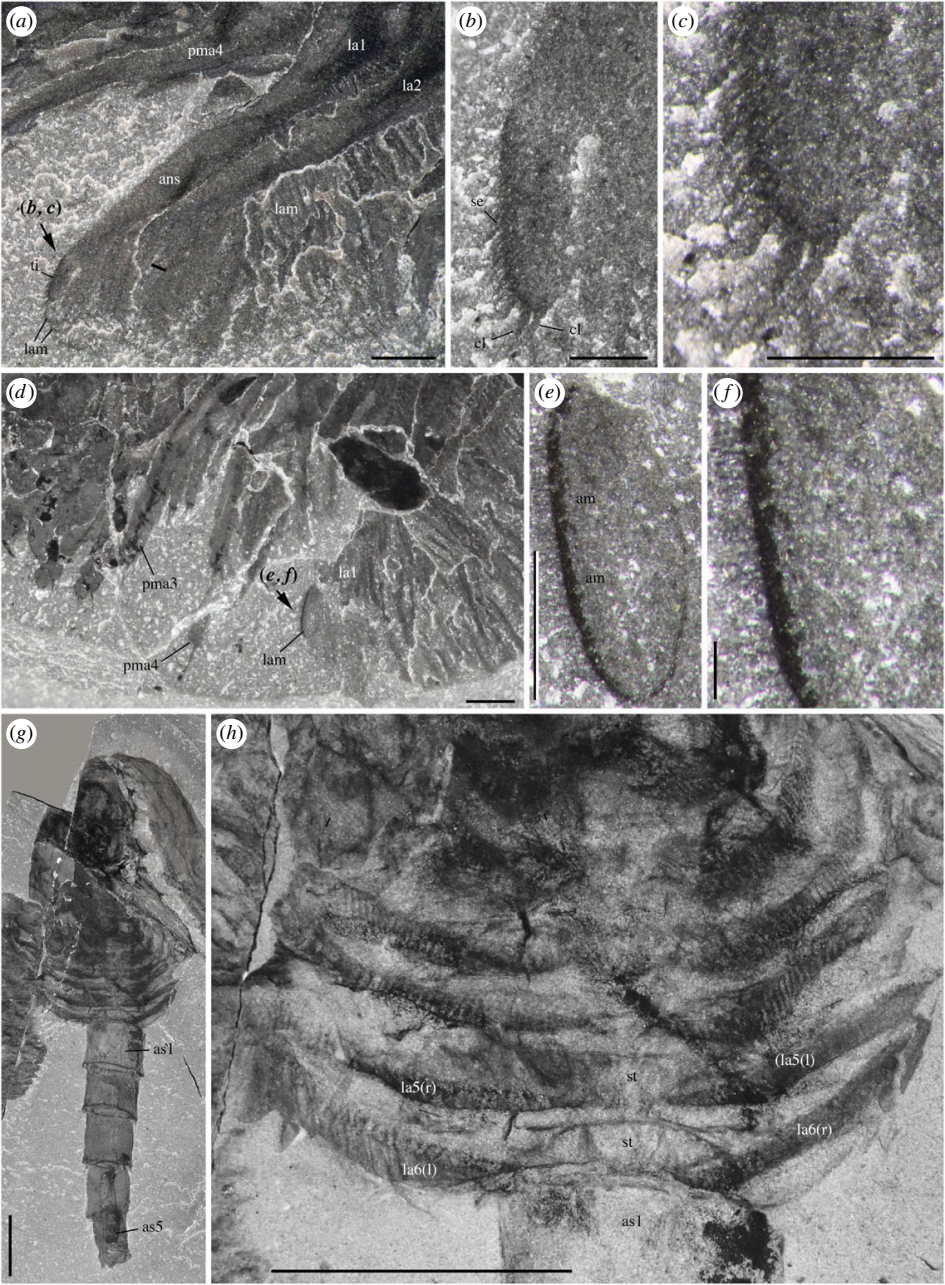
*Waptia fieldensis* Walcott, 1912 [10] from the middle Cambrian (Series 3, Stage 5) Burgess Shale, British Columbia, Canada; lamellate post-cephalothoracic appendages. (*a–c*) ROMIP 64579; general view of the 1st and 2nd lamellate post-cephalothoracic appendages, details of the appendage tip showing terminal claws and marginal setae (location in *a*). (*d–f*) ROMIP 64288; general view and details of the lamella fringed with short setae (location in *d*). (*g,h*) GSC 81173, ventral view showing attachment of lamellate appendages. All images are photographs taken under cross-polarized light. Abbreviations are as follows: arm, arthrodial membrane; ans, annulated stem, cl, claw; lam, lamella; la1–6, 1st to 6th lamellate post-cephalothoracic appendages; la(l), left lamellate post-cephalothoracic appendage; la(r), right lamellate post-cephalothoracic appendage; pma3 and pma4, post-maxillular cephalothoracic appendages; se, setae; st, sternite; ti, tip of lamellate appendage. Scale bars: 5 mm in *g*,*h*; 1 mm in *a,d*; 500 µm in *e*; 250 µm in *b*,*c*; 100 µm in *f*.

*Description.* The middle tagma (post-cephalothorax, akin to a malacostracan ‘pleon’) of *W. fieldensis* is composed of six somites, each bearing a pair of large uniramous appendages fringed on their margins with numerous elongate lamellae (figures 14–16; electronic supplementary material, S14). It consists of five segments (posterior cephalothoracic segments; pcs1–5). pcs1–4 are relatively short, of equal length (in lateral view), and capped by an inverted U-shaped sclerite. pcs5 is approximately twice as wide as pcs1–4 (e.g. figure 14*a*,*b*,*f*,*g*) and results from the fusion of the fifth and sixth somite. Each appendage consists of a slender, tapering, annulated shaft with notable thickening in its proximal third (figures 14 and 16*b*). This basal enlargement seems to be caused by the presence of a sclerotic socket with a narrow anterior extension covering the proximal third of the appendage (figure 14*b*,*c*). This socket appears as a key element in the articulation of the appendage along the ventral area of the corresponding pcs (figure 17*g*,*h*), paraxially to small trapezoidal sternites (figure 17*h*). Annuli are evenly spaced along the shaft (around 10 per mm), each of them giving rise to an elongated lamella. Although their exact number could not be counted accurately due to overlaps of structures, between 40 and 50 lamellae seem to be attached to the fourth appendage of USNM 275404 (figure 16*b*,*c*; electronic supplementary material, S14), thus covering a large surface area of approximately 15 mm^2^. The lamellae seem to be inserted obliquely along the shaft and slightly overlap each other, recalling the slats of a blind (figure 14*d*,*f*). They run almost perpendicular to the shaft axis in its middle part where they reach their maximum length, and more obliquely towards its tip. However, their original orientation cannot be determined accurately due to flattening. The distal end of each lamella is lobate, giving it a lanceolate aspect, and its margins are fringed with closely packed setae (length < 100 µm; figure 17*d*–*f*). Whether setation occurs around the entire margins of the lamella or is limited to its distal lobe is uncertain. Each shaft terminates as a short, elongate, lobate structure devoid of annuli and lamellae and bearing two tiny claws and numerous setae along its outer margin (figure 17*a*–*c*). This terminal structure can rarely be observed being frequently overlapped by the most distal lamellae.

*Discussion.* The structural identity of these post-cephalothoracic appendages is problematic because post-antennal endopods are not known either to be annulate or to bear lamellae in mandibulates [96,97]. Based on their annulate and lamellate morphology, they best compare to crustaceomorph exopods, as seen among Orsten larvae (e.g. [98]), and especially in *Marrella splendens* [42,99]. However, such morphology remains uncommon among the adults of extinct and extant euarthropod taxa. The main issue of this interpretation arises when considering the fourth post-maxillular appendage (pma4), which displays a stenopodous distal end, presumably corresponding to the endopod, but also a fully annulated basis. Because of the intermediary aspect of this appendage, it is difficult to consider the posterior appendages as uniramous exopods, because their morphology is expressed on the ramus that bears the endopod, on the somite just anterior to them. The only plausible solution to this dilemma for us is to consider that the lamellate appendages are instead differentiated basipods, which would be, as far as we know, a unique euarthropod feature. Although intriguing, the existence of such a trait may be better understood in the context of the diversification of limb bases that would characterize hymenocarines and prefigure the development of coxal adaptations [9].

Strausfeld [15] interpreted the post-cephalothoracic lamellate appendages of *W. fieldensis* as being biramous with a long segmented lamellate endopod and a supposed very short exopod stemming out from the basis and fringed with comb-like lamellae, both borne by an alleged ‘protopodite’. The lamellate appendages of *W. fieldensis* clearly have a single annulated (not segmented *sensu* Strausfeld) shaft and a single series of overlapping long lamellae (e.g. figure 16*d*,*f*). Their thick basal part is similarly annulate (figure 16*b*,*c*) and does not give rise to an additional branch.

The lamellate appendages of *W. fieldensis* could be compared with the phyllobranchiate gills of extant crustaceans such as brachyuran crabs and caridean shrimps [88], which similarly bear closely spaced series of leaf-like lamellae attached to a central axis (electronic supplementary material, S15). The crustacean gill lamellae are by definition flat haemocoelic spaces lined with an epithelium and a thin cuticle. They are an integral part of the vascular system which sustains haemolymph circulation through gills via distinct afferent and efferent microchannels and branchial vessels (e.g. [100]). The high number of lamellae in *W. fieldensis*, which offer a large, double-sided exchange surface with the surrounding medium, suggests a role in gaseous (e.g. oxygen uptake) and ionic exchange. The cuticle of the lamellae seems to have been thin enough (e.g. figure 16*f*) to allow gaseous diffusion and perfusion with haemolymph. The size, position and orientation of lamellae also suggest a swimming function (figure 14*a*–*f*). The large surface area provided by multiple overlapping lamellae has an optimal design for pushing water back via rhythmic or more occasional power strokes. In summary, the lamellate appendages of *W. fieldensis* are likely to have performed several vital functions such as gaseous and ionic exchanges, drag-powered swimming and self-ventilation over gill-like lamellae via the water currents generated by locomotion.

The lamellate appendages of *W. fieldensis* also recall those of artiopodans, including trilobites, and ‘megacheiran’ arthropods, including trilobites, which have exopods fringed with lamellae (e.g. *Leanchoilia*, *Naraoia, Olenoides, Sidneyia*). In spite of their similarity with lamellate structures in chelicerates and mandibulates, the respiratory role of these relatively smaller lamellae was questioned by Suzuki and Bergström [101], who argued that the expansion of lamellar surface with growth in these taxa—extrapolated from extant models (e.g. *Limulus* and decapods)—was too small to meet the animal's respiratory requirements and that oxygen uptake occurred via other respiratory surfaces. In *W. fieldensis* the reduced individual surface area of the lamellae seems to have been compensated for by their very large number along seven pairs of appendages (including the lamellate margin pma4), thus representing a large exchange surface with the surrounding water. If not sufficient to ensure oxygenation alone, the spacious inner surface of its carapace may have functioned as a complementary site for oxygen diffusion, as is the case in numerous extant crustaceans (e.g. *Nebalia*, myodocope ostracods [102,103]).

#### 5.2.7. Posterior tagma (abdomen)

**Figure 18.**
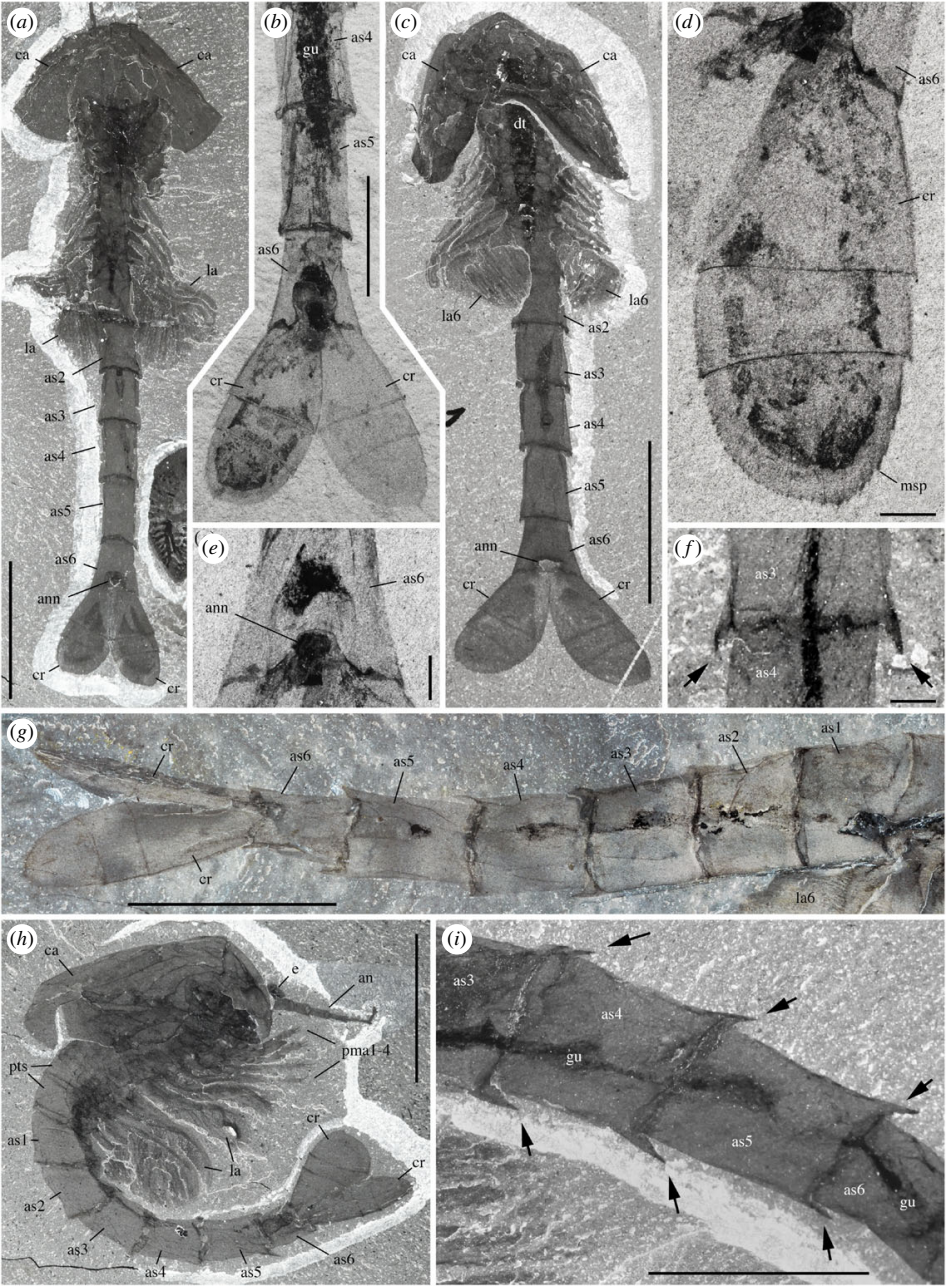
*Waptia fieldensis* Walcott, 1912 [10] from the middle Cambrian (Series 3, Stage 5) Burgess Shale, British Columbia, Canada; posterior part. (*a*) ROMIP 64295, ventral view. (*b*) USNM 275504, ventral view, details of posterior part (overall view, figure 15*a*). (*c*) ROMIP 64296, general ventral view. (*d,e*) USNM 138231 (counterpart of USNM 275504), details of caudal ramus and anal region. (*f*) USNM 83948 k, details of posterior segment boundaries. (*g*) USNM 57682, details of posterior segments in sublateral view. (*h*) ROMIP 56947, specimen with strongly curved posterior region. (*i*) ROMIP 64281, details of posterior segment margins. All images are photographs taken under cross-polarized light. Black arrows indicate marginal spines on posterior segments. Abbreviations are as follows: an, antennule; ann, anal notch; as1–6: 1st to 6th abdominal segments; ca, carapace; cr, caudal ramus; e, eye; gu, gut; la1–6, 1st to 6th lamellate post-cephalothoracic appendages; msp, marginal spines of caudal ramus; pts, post-cephalothoracic segment. Scale bars: 1 cm in *a,c,g,h*; 5 mm in *b,i*; 1 mm in *d–f*.

**Figure 19.**
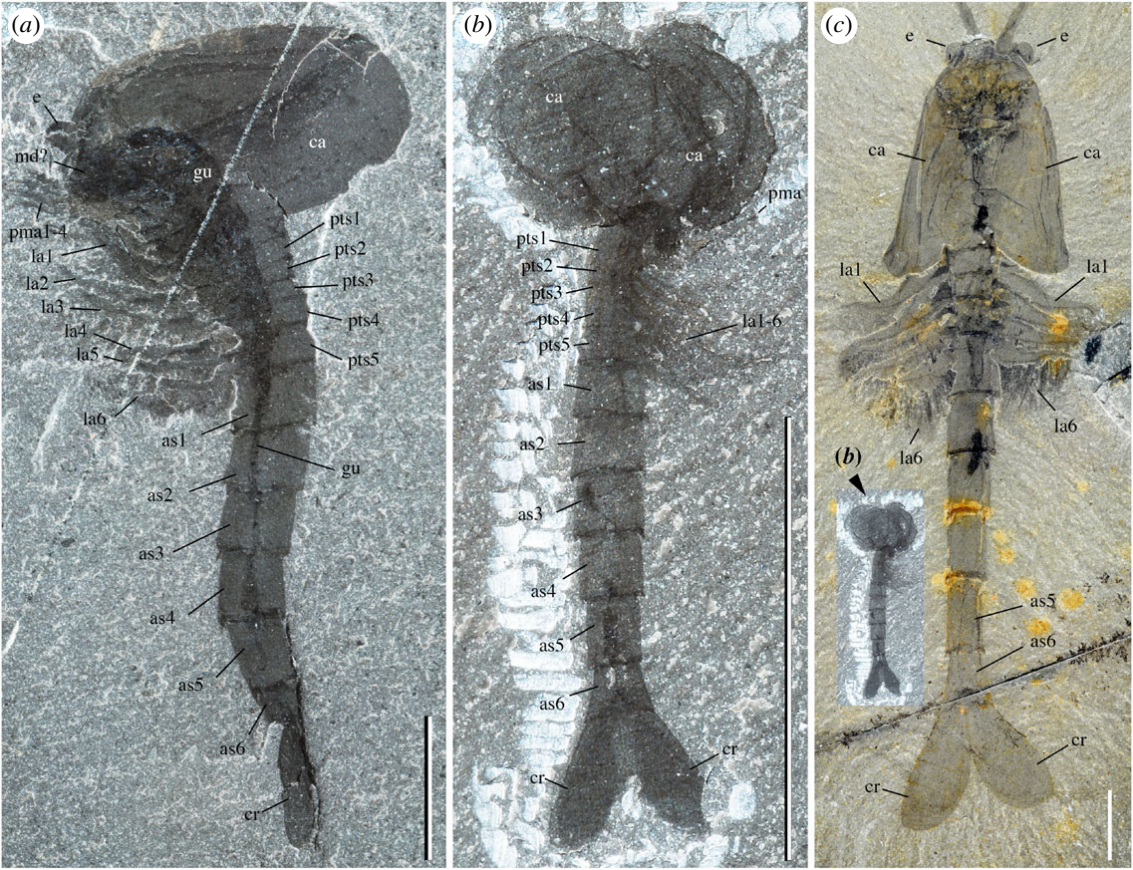
*Waptia fieldensis* Walcott, 1912 [10] from the middle Cambrian (Series 3, Stage 5) Burgess Shale, British Columbia, Canada; juvenile stages. (*a*) USNM 268338, lateral view. (*b*) USNM 165229, dorsal view. (*c*) USNM 57681, ventral view (smallest juvenile at the same scale; see *b*). All images are photographs taken under cross-polarized light. Abbreviations are as follows: as1–6, 1st to 6th abdominal segments; ca, carapace; cr, caudal ramus; e, eye; gu, gut; la1–6, 1st to 6th lamellate post-cephalothoracic appendages; md, mandible; pma1–6, 1st to 6th post-maxillular cephalothoracic appendages; pts1–5, 1st to 5th post-cephalothoracic segments. Scale bars: 5 mm.

*Description.* The posterior tagma of *W. fieldensis* is elongate (figure 18), limbless and consists of six segments and a pair of caudal rami which together represent approximately 60% of the total body length. The outline of segments is subquadrangular in lateral, dorsal and ventral views (figure 18*a*,*c*,*g*). They articulate via well-developed arthrodial membranes which ensure a high degree of flexibility of the posterior region of the animal, as shown by a wide range of curved and sigmoidal postures (figures 1 and 16*h*). The length to width ratios (L/W) of posterior segments 1 to 5, measured in 12 specimens preserved laterally, are in the ranges of 0.83–0.97, 0.91–1.23, 1.04–1.32, 1.16–1.59 and 1.54–2 (electronic supplementary material, S16), respectively, indicating that segments become increasingly elongate and smaller posteriorly. Specimens preserved dorsally and ventrally have a slightly higher L/W mean value (electronic supplementary material, S16), suggesting that the posterior segments are not perfectly cylindrical but rather slightly compressed laterally. Segments in juveniles (figure 19) are less elongated than those of adults and subadults and have a comparatively lower L/W ratio (electronic supplementary material, S16). The posterior margin of each abdominal segment is armed with two pairs of strong, slightly oblique, posteriorly directed spines, one ventral and one dorsal (figure 18*i*). The last posterior segment appears as a stout truncated cone (figure 18*b*,*e*) around half the length of the previous segment but significantly wider posteriorly than all the other segments. It has a central, rounded notch along its ventral margin, accommodating the opening of the anus (figure 18*e*). Two sub-elliptical, caudal rami are inserted dorsally into the last abdominal segment (figure 18*a*,*c*). Although flat, they seem to have contained internal tissues as suggested by dark areas in several specimens (e.g. figure 18*d*). Their longitudinal axes form an approximately 20–45° angle with the sagittal plane of the animal. Their length is approximately equal to that of the last two abdominal segments combined. The rami seem to have been able to slightly rotate horizontally (e.g. figure 18*a*,*c*). Each ramus is divided into three pseudo-segments by two lines running perpendicular to the long axis of the ramus (figure 18*d*). These flat plate-like pseudo-segments do not overlap and show no articulated features comparable with those of the appendages. The rounded margin of the distalmost pseudo-segment bears regularly spaced tiny spines and the distal corners of the remaining pseudo-segments are pointed (figure 17*d*). Tripartite caudal rami are unknown in modern crustaceans.

*Discussion.* None of the best specimens of *W. fieldensis* that we have observed shows additional setae in the inner part of the caudal rami as they appear in Strausfeld ([15]; figs 5e, 6b). The spines which occur along the lobate margin of rami are extremely short and do not resemble setae. They seem to be tiny cuticular marginal outgrowths and can hardly be compared with mechanoreceptors involved in the control of abdominal motion (see [15, p. 10] and modern analogues [104]).

#### 5.2.8. External morphology: reconstructions

**Figure 20.**
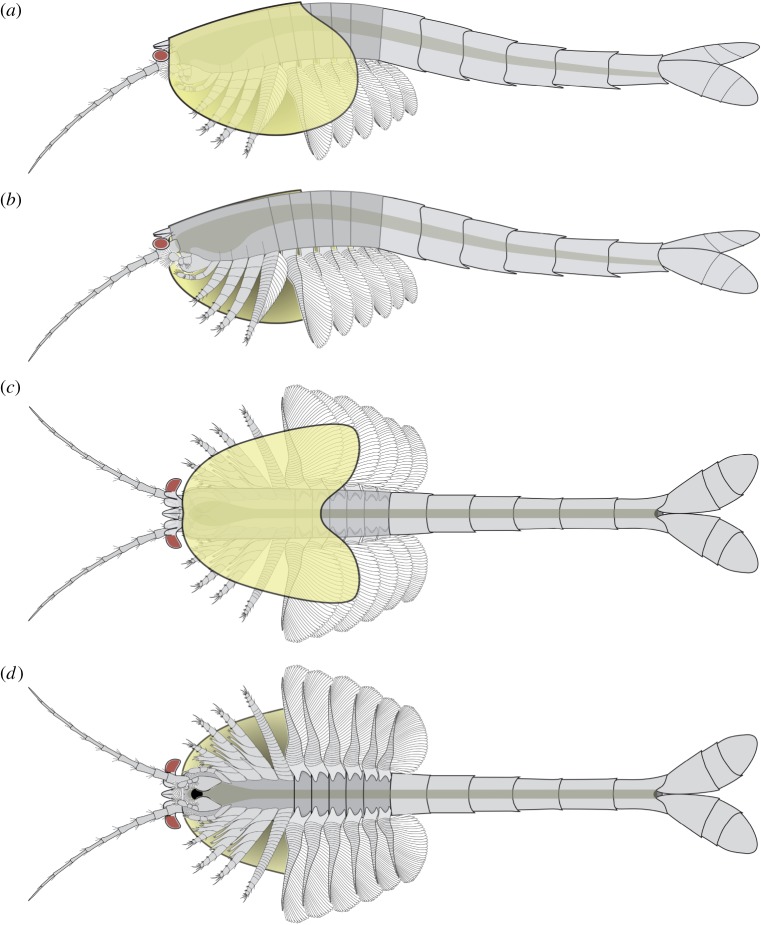
*Waptia fieldensis* Walcott, 1912 [10] from the middle Cambrian (Series 3, Stage 5) Burgess Shale, British Columbia, Canada; reconstruction. (*a*) Left lateral view; (*b*) left lateral view with the left valve removed to show cephalothoracic appendages; (*c*) dorsal view; (*d*) ventral view with the lamellate appendages spread apart. Assumed translucent carapace in light yellow. Digestive tract in dark green (frontal part and mouth orientation hypothetical). See also videos in electronic supplementary material, S23 and S24.

**Figure 21.**
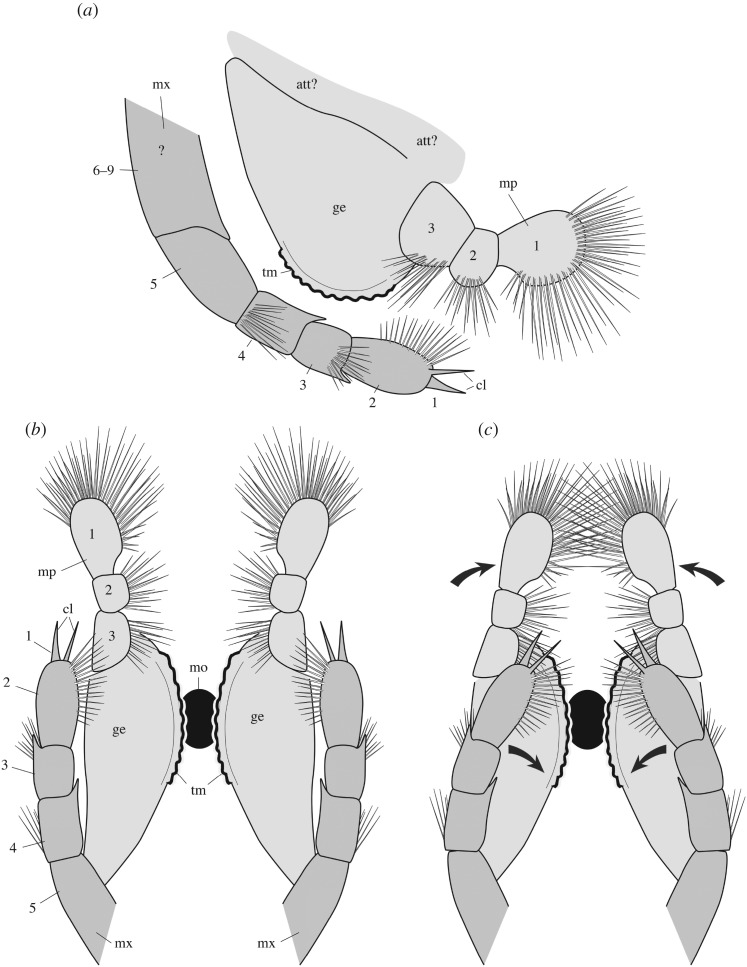
*Waptia fieldensis* Walcott, 1912 [10] from the middle Cambrian (Series 3, Stage 5) Burgess Shale, British Columbia, Canada; reconstruction of mandibles and maxillules. (*a*) Right lateral view. (*b,c*) Ventral views showing possible rotation (black arrows) of the mandibular palps and the maxillule towards the mouth region. The detailed morphology of the most proximal part (e.g. attachment to body) of the mandibles and maxillules is uncertain. Abbreviations are as follows: att?, attachment of mandible to body; cl, claw; ge, gnathal element of mandible; md, mandible; mo, mouth; mp, mandibular palp; mx, maxillule; tm, toothed margin; 1–3, 1st to 3rd podomeres of mandibular palp; 1–9, 1st to 9th podomeres of maxillule.

**Figure 22.**
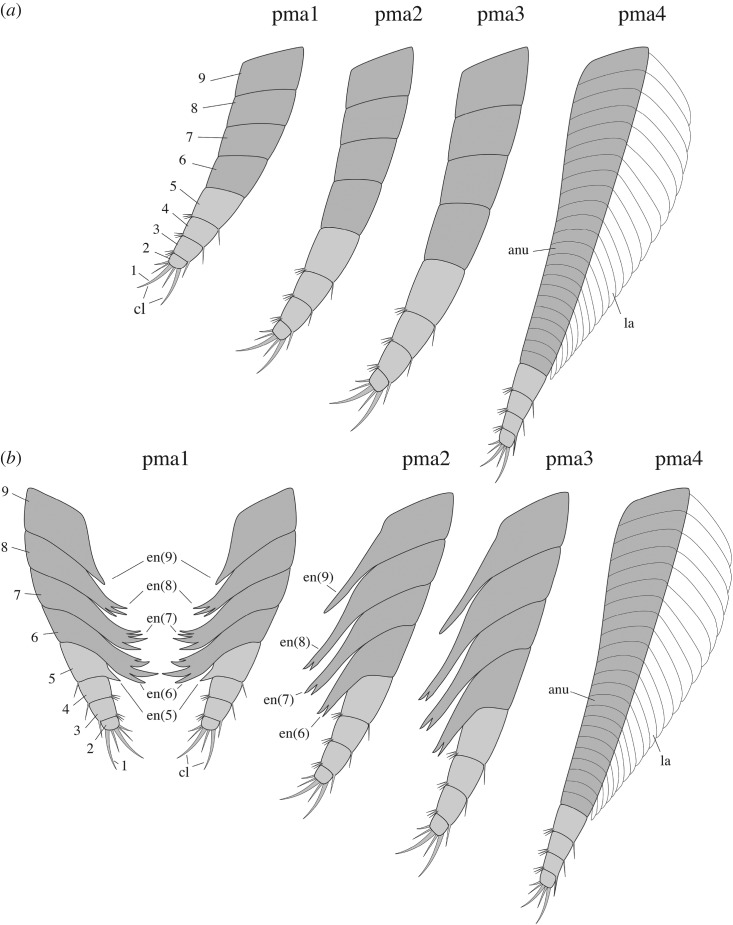
*Waptia fieldensis* Walcott, 1912 [10] from the middle Cambrian (Series 3, Stage 5) Burgess Shale, British Columbia, Canada; reconstruction of the post-maxillular cephalothoracic appendages. (*a*) Lateral views. (*b*) Mesial views showing endites along the inner margin of the 1st to 3rd appendages; first pair of appendages showing both limbs with converging endites. Podomeres 1–5 and 6–9 represent the endopod and the basipod, respectively. See also videos in electronic supplementary material, S23 and S24. All drawings at the same scale. Abbreviations are as follows; anu, annulus; cl, claw; en(6)-en(9), endite of the 6th to 9th appendage podomeres; lam, lamella; pma1–4, 1st to 4th post-maxillular cephalothoracic appendages; 1–9, 1st to 9th podomere of appendages.

**Figure 23.**
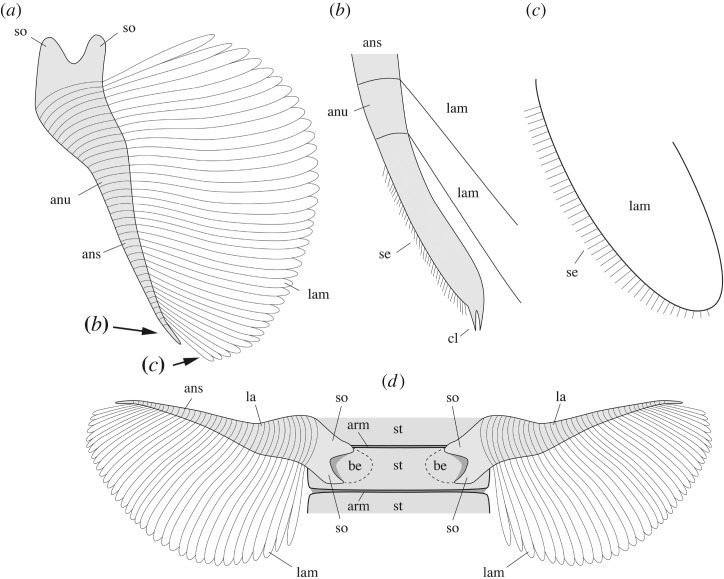
*Waptia fieldensis* Walcott, 1912 [10] from the middle Cambrian (Series 3, Stage 5) Burgess Shale, British Columbia, Canada; reconstruction of the lamellate post-cephalothoracic appendages. (*a*) Lateral view of left appendage (annulated stem and lamellae in grey and white, respectively). (*b*) Details of the distalmost part of the appendage (see location in *a*). (*c*) Setae along the margin of the lamella (see location in *a*). (*d*) Ventral view showing attachment of appendages; detailed morphology of attachment to the body uncertain (basalmost element represented in dotted lines). See also videos in electronic supplementary material, S23 and S24. Abbreviations are as follows: anu, annulus; ans, annulated stem; arm, arthrodial membrane; be, basalmost element of appendage; cl, claw; la, lamellate post-cephalothoracic appendage; lam, lamella; se, setae; st, sternite.

The general external morphology of *W. fieldensis* is reconstructed in figure 20. Morphological details of its mandibles and maxillules, anterior and posterior cephalothoracic appendages are presented in figures 21–23, respectively.

#### 5.2.9. Internal anatomy

##### 5.2.9.1. Nervous system

**Figure 24.**
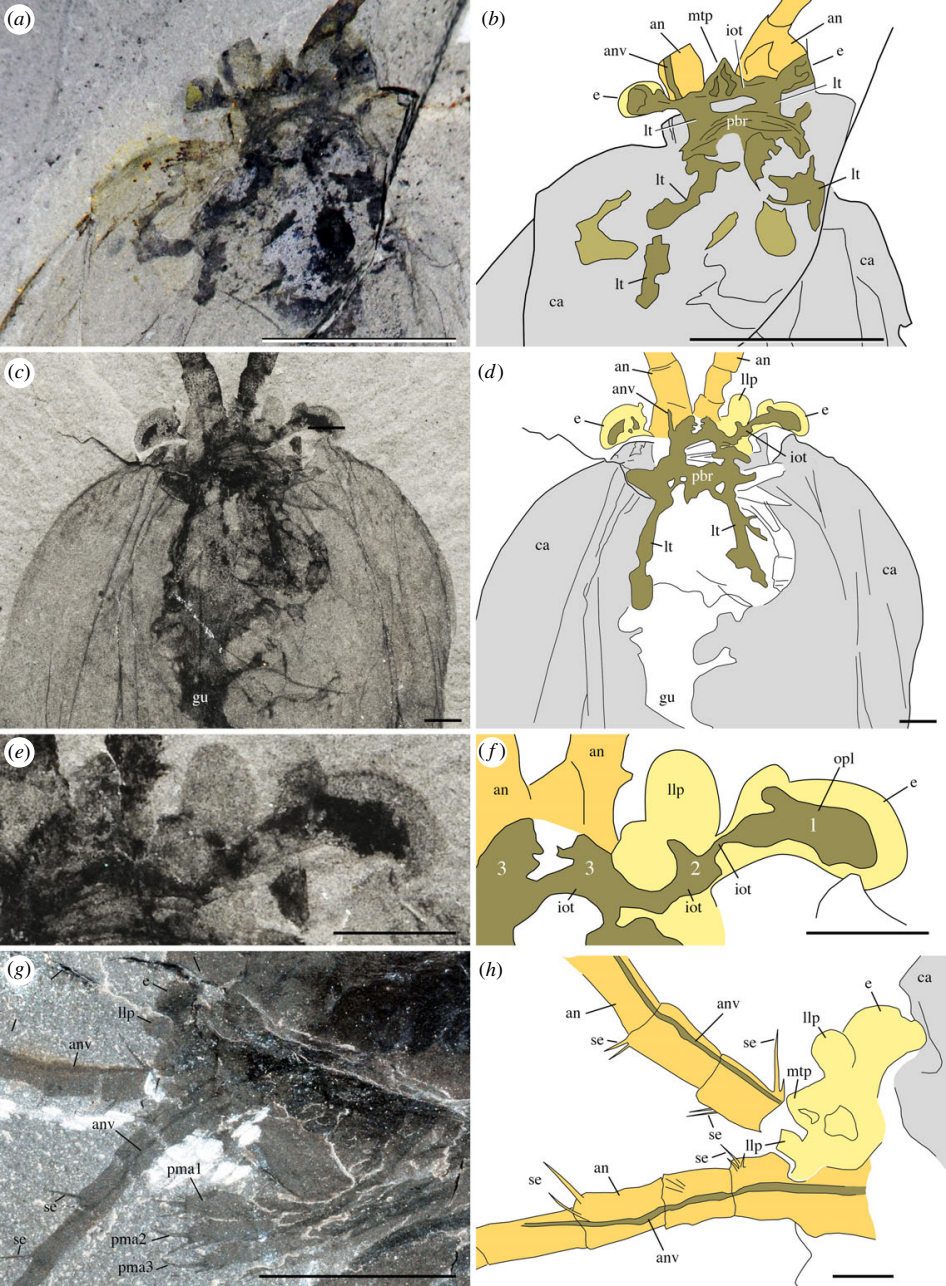
*Waptia fieldensis* Walcott, 1912 [10] from the middle Cambrian (Series 3, Stage 5) Burgess Shale, British Columbia, Canada; neural tissues. (*a,b*) ROMIP 64293, anterior part in dorsal view. (*c–f*) USNM 138231, anterior part; general view and details of eye region; 1–3 are small lobate features interpreted as three possible protocerebral elements: the optic lobes, the lateral protocerebrum and the median protocerebrum, respectively. (*g,h*) USNM 268270, details of antennules. *a,c,e* are photographs taken under cross-polarized light. Frontal part of body in yellow, antennules in light orange and neural tissues in dark green. Abbreviations are as follows: an, antennule; anv, antennular nerve; ca, carapace; e, eye; gu, gut; iot, interoptic tract; lt, longitudinal tract; mtp, median triangular projection; opl, optic lobe; pbr, posterior bridge; pl, peduncular lobe; pma1–3, 1st to 3rd post-maxillular cephalothoracic appendages; se, setae; 1–3, protocerebral elements. Scale bars: 5 mm in *a,b,e*; 1 mm in *c,d,f*.

*Description.* Two specimens (USNM 138231, ROMIP 64293; figure 24) in which the post-antennular appendages are not preserved reveal a symmetrical network of dark tracts running through the frontal part of the cephalothoracic region. This network consists of an anterior, transversal, interoptic tract (IOT) which links the two eye lobes through the median triangular projection and the eye stalks (figure 24*a*–*d*). It is connected anteriorly to a pair of much narrower branches extending into the proximal part of antennules (figure 24*f*), and posteriorly to two longitudinal, slightly diverging tracts. An additional bridge parallel to the IOT connects the longitudinal tracts, thus creating a quadrangular ‘window’ posterior to the median triangular projection.

This network clearly runs through visual (stalked eyes) and assumed sensory (antennules) organs and cannot be confused with digestive features (e.g. bilaterally symmetrical ramified glands attached to a central gut; e.g. [105]). The most plausible interpretation is that it represents the fossilized remains of neural tissues comparable with those found in other Cambrian arthropods [85,106,107]. The three-dimensional architecture of the nervous system of *W. fieldensis* cannot be reconstructed with the same accuracy as that of modern arthropods (e.g. [108,109]) because of the stacking of fossilized neural and other tissues due to compression. However, two of the three neuropil centres of the arthropod brain can be identified in *W. fieldensis*: the protocerebrum with paired optic lobes and a medial protrusion, followed by a deutocerebrum probably innervating the antennules and delimiting the anterior part of the stomodeum.

**Figure 25.**
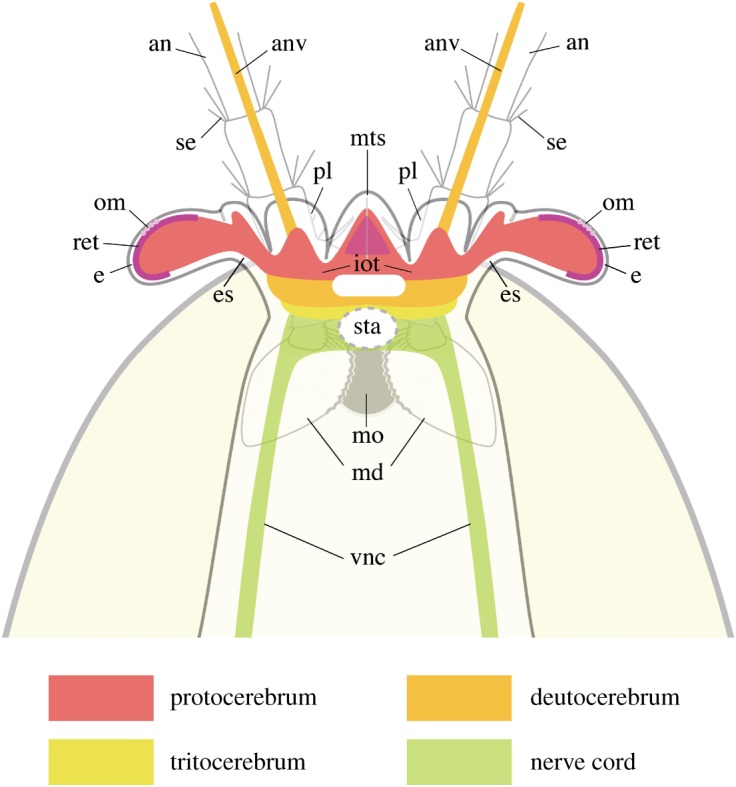
*Waptia fieldensis* Walcott, 1912 [10] from the middle Cambrian (Series 3, Stage 5) Burgess Shale, British Columbia, Canada; idealized reconstruction of the central nervous system showing the protocerebrum, deutocerebrum and possible tritocerebrum. Abbreviations are as follows: an: antennule; anv: antennular nerve; e, eye; es, eye stalk; iot, interoptic tract; llp, lobe-like projection; md, mandible; mo, mouth; mts, median triangular sclerite; om, ommatidia; ret, retina (receptor cells); se, setae; sta, stomodeal aperture; vnc, ventral nerve cord.

*Discussion.* It appears that the stomodeal area could lie posterior to the IOT and the following nerve mass, thus creating a second window-like medial interruption of the central nervous system (figure 25). A comparable configuration is found in fuxianhuiids ([106] fig. 2d), but in this case the post-protocerebral opening is much larger and clearly followed by a thick nervous concentration, and we think therefore that this opening is most probably the stomodeal aperture itself. Ma *et al*. ([110], figs. 1–4) decided to reject their original interpretation of a large post-protocerebral aperture despite clear and redundant fossil evidence, but we think that their first reconstruction was correct, as is also supported by the evidence provided by *Waptia*. Both USNM 138231 and ROMIP 64293 show a rather small post-protocerebral window closed posteriorly by a relatively thin bridge, in a location which seems too anterior for the expected insertion of the mandibles. It is likely however that, in both cases, the physical location of the mouth does not directly align dorsoventrally with the stomodeal aperture because of the oesophageal ‘loop’ and its anteriad direction distally. If indeed an extra ‘post-protocerebral’ aperture existed in *Waptia*, it would be a unique feature among euarthropods that could challenge hypotheses of conservativeness of brain morphology in fossil and extant taxa [110,111].

Further supporting relationships between fuxianhuiids and hymenocarines, the peduncular lobes described in *Fuxianhuia* may be homologous to the same lobes in *Waptia*. Comparisons with the neuroanatomy of pancrustaceans [106] are also further justified by our phylogenetic resolution of hymenocarines (see below). In decapods, for example, the protocerebrum consists of three compartments: (i) the optic lobe (lamina, medulla and lobula) which processes visual signals, (ii) the lateral protocerebrum with the medulla terminalis and, anteriorly, the hemiellipsoid body, which participates in the olfactory pathway, and (iii) the median protocerebrum with, posteriorly, two bilaterally paired, largely fused, neuropils [85]. By their antero-median topology, the peduncular lobes of fuxianhuiids and hymenocarines seem to correspond best to the hemi-ellipsoid bodies, implying an olfactory function.

Strausfeld ([13], fig. 6; [14], fig. 12.10; [15], fig. 6c,d) made a tentative reconstruction of the brain of *W. fieldensis* based on the assumed traces of neural tissues observed in USNM 83948j. The re-examination of the part and counterpart of this specimen (electronic supplementary material, S5) under polarized light failed to reveal any well-defined dark areas that could be convincingly interpreted as a possible brain and associated neural tissues, except isolated patches of carbonaceous films within the eye lobes and the median triangular projection (compare electronic supplementary material, S8c with [15], fig. 6c,d). The only sound evidence for a symmetrical network of neural tissues within the head of *W. fieldensis* comes from two other specimens (USNM 138231, ROMIP 64293) described in the present paper (figure 24). Our reconstruction of the nervous system of *W. fieldensis* (figure 25) agrees on certain points with that of Strausfeld [13–15], such as the presence of a protocerebrum with a possible mesial organ and a deutocerebrum innervating antennules.

##### 5.2.9.2. Digestive system

**Figure 26.**
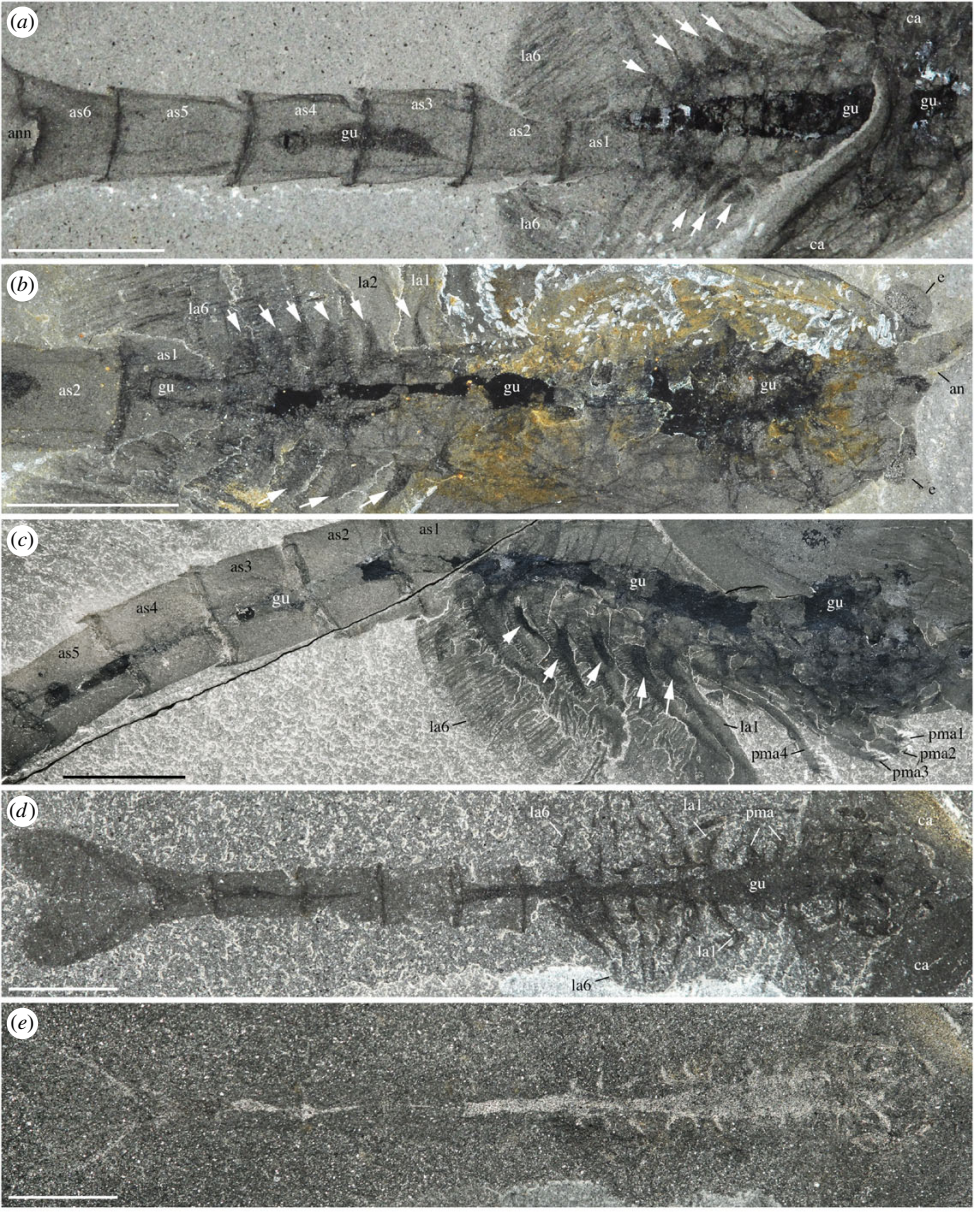
*Waptia fieldensis* Walcott, 1912 [10] from the middle Cambrian (Series 3, Stage 5) Burgess Shale, British Columbia, Canada; digestive system. (*a*) ROMIP 64296, ventral view showing the anterior part of the gut (see also figure 17*c*). (*b*) USNM 57681a (counterpart), ventral view. (*c*) ROMIP 64579, lateral view showing the gut from the fifth abdominal segment to the cephalothoracic region. (*d,e*) ROMIP 64282, partly decayed specimen. Small white arrows indicate internal dark elongated features within the proximal part of lamellate post-cephalothoracic appendages (see also figure 14). Abbreviations are as follows: an, antennule; ann, anal notch; as1–6, 1st to 6th abdominal segment; ca, carapace; e, eye; gu, gut; la1–6, 1st to 6th lamellate post-cephalothoracic appendages; pma1–4, 1st to 4th post-maxillular cephalothoracic appendages. Scale bars: 5 mm.

*Description.* The digestive system of *W. fieldensis* is represented by a long cylindrical axial tract running from the mouth area through to the anus which opens along the ventral margin of the last abdominal segment. The gut appears as a relatively narrow tube in the posterior part of the body (diameter about 0.65 mm in figure 26*c*), which expands gradually from the post-cephalothoracic region into a larger anterior tubular pouch (figure 26*a*,*b*,*d*,*e*). Its overall shape is revealed by continuous or patchy carbonaceous films (figure 26*a*–*c*) and by a strong enrichment in C (elemental maps; figure 2*d*). Local alternating swellings and constrictions (electronic supplementary material, S17 and S18) suggest that the gut was sufficiently flexible to accommodate various quantities of food or undigested wastes and that the movement of ingesta may have been regulated by peristaltic contractions of the gut as is the case in modern crustaceans (e.g. [112,113]). The ‘ganglionic’ aspect of posterior intestinal swellings, as well as occasional ramifying filaments possibly caused by partial decay (electronic supplementary material, S18) illustrates the sometimes confounding taphonomy of internal features for Burgess Shale-type fossils, and the importance of using caution and redundant evidence when interpreting, for instance, neural or vascular tissues.

*Discussion.* Neither food elements such as undigested sclerites, setae, chaetae nor ingested sediment could be recognized within the gut of *W. fieldensis,* thus contrasting with other Burgess Shale animals such as *Ottoia* [6] and *Sidneyia* [105]. The anterior part of the gut tract gut of *W. fieldensis* is clearly enriched in phosphorus and calcium (figure 2*g*,*h*), indicating possible phosphatic mineralization in apatite, as is common of gut preservation in the Burgess Shale. Comparable mineralization occurs in various parts of the digestive system of Cambrian arthropods, such as paired anterior glands [105,114–117] and the abdominal pocket of *Sidneyia* [105], and is responsible for the extremely fine three-dimensional preservation of the former digestive tissues. It has been interpreted [105,116] as possibly resulting from the recrystallization of original calcium phosphate mineral concretions (spherites) secreted by the gut epithelium and released into the gut tract after death. This interpretation is supported by the presence of such spherites within the gut of extant horseshoe crabs ([105], fig. 12G), which represent an important source of P and Ca for vital processes such as moulting. We see no clear evidence of digestive glands along the gut of *W. fieldensis*. Paired subtriangular projections also represented by a denser carbonaceous imprint (figure 2*c*,*d*; see also §5.2.9.3) do occur in the post-cephalothoracic region. They reach the periphery of the gut but do not connect with it (figure 15*a*,*b*), ruling out the possibility of being digestive diverticles or glands (see §5.2.9.3). The anterior end of the gut seems to bend downwards (electronic supplementary material, S17f) to form the oral opening, but the exact morphology and orientation of the mouth opening remains unclear partly due to the concentration and overlap of various tissues (e.g. neural, digestive) and sclerotized structures (anterior appendages) in this particular area.

##### 5.2.9.3. Other internal features

Numerous specimens show dark subtriangular traces running longitudinally through the proximal portion of their appendages. They are particularly well developed in the six post-cephalothoracic lamellate appendages (figures 15 and 26*c*) and seem to be present also in the post-maxillular cephalothoracic appendages. Their strong enrichment in C (figure 2*c*,*d*) suggests that they might represent internal tissues within the appendage stems. These features have already been mentioned in other Burgess Shale arthropods (e.g. [37,69,118]), and have been discussed as being either internal cavities (such as haemolymph channels [69]) or, less convincingly, adjunctions of the gut tract. Comparable features have also been described in Cambrian lobopodians from China (e.g. *Paucipodia* [119]) and interpreted as the extensions of a central coelomic or haemocoelic cavity into the legs. Similarly, we assume that the triangular ‘tonguelets’ of *W. fieldensis* are serial projections of a peri-intestinal coelomic cavity, aiding in the lateral distribution of haemolymph.

## 6. Phylogenetic affinities

### 6.1. Previous views

*Waptia fieldensis* is one of the Cambrian arthropods that most evidently recalls present-day crustaceans. Overall comparisons with extant taxa have led authors to place *Waptia* within or close to Branchiopoda (e.g. [10]) within Malacostraca (possibly Leptostraca [120–122]), Maxillopoda [24], Crustacea [22] or Mandibulata (possibly Pancrustacea [15]). The cladistic analyses of Briggs & Fortey [123] and Wills *et al*. [23] resolved it as a nested member of Crustacea. However, most of these tentative placements were undermined by the lack of a comprehensive morphological and anatomical revision, especially with respect to the type and arrangement of appendages. For the same reason, *Waptia* has been absent from more recent phylogenetic treatments of Euarthropoda [9,123].

### 6.2. Cladistic results

**Figure 27.**
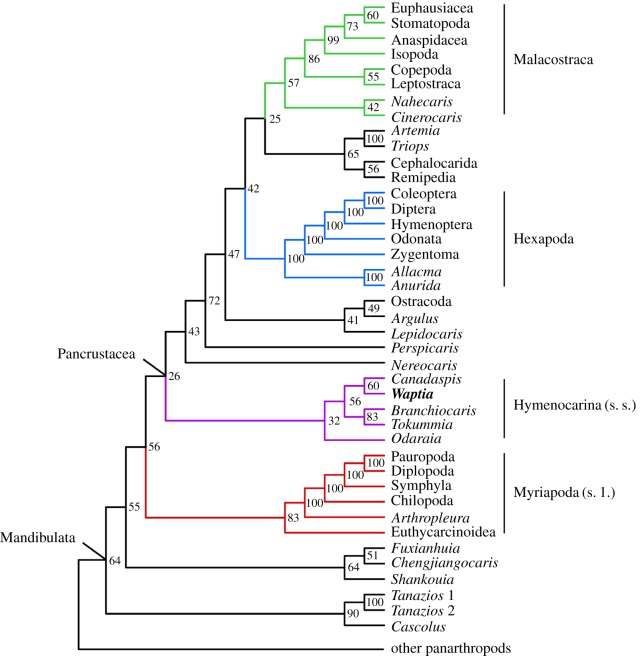
Mandibulate clade extracted from a maximum clade credibility tree of a time-calibrated Bayesian analysis of adult panarthropod relationships (Mkv + Γ model, 85 taxa, 219 characters; see electronic supplementary material, S19–S21). Numbers on the right of the nodes are posterior probabilities.

Compared to the previous cladogram of arthropod relationships upon which the present analysis is based [9], the most important difference relevant to *Waptia* in our main topology (figure 27; electronic supplementary material, S19–21) is the retrieval of hymenocarines as stem pancrustaceans, instead of sister taxa to all other mandibulates. *Waptia* is thus not only retrieved with other hymenocarines, but results in the placement of this group of bivalved taxa closer to the paraphyletic crustaceans. This grouping is notably influenced by our interpretation of a five-segmented endopod in the cephalic limbs of *Waptia*.

As other potentially important characters defining Pancrustacea (e.g. epipods, see below) are still unclearly optimized on the tree, this coding probably plays an important role into moving hymenocarines from a basal mandibulate position [9] to Pancrustacea *sensu lato*.

The presence of a palp on the mandibles of protocaridids and other hymenocarines was coded as uncertain, and therefore these characters probably contributed little to this change of configuration, optimized as probably plesiomorphic for Mandibulata. Likewise, *Nereocaris* was added with several uncertainties regarding the anatomy of its head or the morphology of its limbs and was not determinant in this topological change.

The origin of Mandibulata *per se* is here embodied by fuxianhuiids, as found previously [9], but also by the Herefordshire taxa *Cascolus* [124] and *Tanazios* [125], newly added to this analysis. We discuss the possible implications of this result for the origin of the mandibulate body plan below.

The broad phylogenomic analysis of Regier *et al*. [32] supported the monophyly of Pancrustacea, composed of the following distinct lineages: Oligostraca (ostracods, mystacocarids, branchiurans and pentastomids), Vericrustacea (malacostracans, thecostracans, copepods and branchiopods), Xenocarida (cephalocarids and remipeds) and Hexapoda. A more recent molecular study focusing on the crustacean–hexapod relationships [33] favours an alternative topology in which branchiopods are grouped with hexapods and a polyphyletic Xenocarida into a clade coined Allotriocarida. Our own analysis yields a third configuration in which Xenocarida and Branchiopoda form a sister clade to Malacostraca, further illustrating the problematic resolution of branchiopods and xenopods, as well as the difficulty of elucidating hexapod origins based on morphology alone. However, the well-supported placement of oligostracans as basalmost pancrustaceans in both of these molecular studies and other works provides a robust benchmark to assess the role of fossils in the origin of Pancrustacea.

In this context, it is worth noting that some hymenocarine forms (*viz*. *Perspicaris* [126] and *Nereocaris* [53]) are placed as a grade to extant Pancrustacea. This could offer an insight into the significance of abundant Palaeozoic ‘phyllocarids’ (archaeostracans; e.g. [127]). Indeed, these taxa, rather than being immediately derived and confined as relatives of leptostracans, could have represented the plesiomorphic pancrustacean body plan, out of which secondarily derived lineages would have emerged (oligostracans, branchiopods and xenocarids). The question, therefore, is whether *Nahecaris* [128] and *Cinerocaris* [129]—two Palaeozoic phyllocarids with known soft parts retrieved here as basal malacostracans—are especially derived among archaeostracans, or if they generally represent the anatomy of these taxa, leaving hymenocarines as the sole representatives of an ancestral ‘phyllocarid-like’ body plan.

We provide in electronic supplementary material, S22, an alternative topology resulting from the inclusion of selected larval taxa, as was done in a previous iteration of this dataset [9]. The implementation of larvae is of interest given the additional data it provides and the opportunity to investigate the possible placement of these forms on the tree—leading to an analysis of evolutionary–developmental trends, such as heterochrony. However, any interpretation of such analysis must be taken with great caution, precisely because of evo-devo and the fact that comparing character states between different ontogenetic stages may not make any evolutionary sense [130]. In particular, ‘recapitulating’ larvae (i.e. larvae displaying traits similar to the ancestral conditions of their adult forms) may resolve much more basally than their corresponding adults.

The larva-inclusive topology differs in some fundamental aspects from the adult-only one. Outside of Mandibulata, megacheirans form a sister group to Arachnomorpha, instead of being, surprisingly, retrieved as closer to mandibulates. In both cases, this configuration has very low posterior probability support, which highlights the ambiguous placement of megacheirans—and, as a corollary, the still poorly understood earliest radiation of euarthropods.

Importantly, hymenocarines do not resolve as stem pancrustaceans in this context, but instead are grouped with a large ‘panmyriapod’ clade, also inclusive of euthycarcinoids and fuxianhuiids. As such, they also represent earliest mandibulates, whereas the origin of pancrustaceans now falls onto *Cascolus* and *Tanazios*, as well as the ‘Orsten’ crustaceomorphs—as traditionally viewed by Waloszek & Müller (e.g. [97]). This is not surprising insofar as these taxa illustrate ‘ideal’ transitional states, both morphologically and anatomically, before the later specializations in pancrustaceans, especially of the mandibles and maxillules. However, this is different from the previous analysis of this dataset [9], in which these taxa were found to be derived crustaceans. This suggests that this scenario was also facilitated by the revision of *Waptia* and the addition of Herefordshire forms.

This topology would imply that the well-defined mandibles of hymenocarines—lacking exopods and bearing highly modified endopods—are traits independently characterizing the origin of the myriapod lineage, rather than being very early derived states in the evolution of crustaceans. This is found here to be the most likely solution (in a Bayesian sense) for hymenocarines to coexist with the traditional view of crustacean evolution triggered by the discovery and description of the Orsten material.

However, as mentioned above, it is difficult to justify the need for such a solution as long as the adult forms of the Orsten larvae are not known. They may be hymenocarines themselves, or, as our topology could suggest, taxa with affinities to *Cascolus* and *Tanazios*. In both cases, the topology would be optimized as presented in figure 27. This hypothesis is also weakened by our observations of the many similarities between *Waptia* and pancrustaceans, especially in comparison to the much more ambiguous identity of morphological features in a taxon like *Cascolus*.

### 6.3. Discussion and evolutionary implications

#### 6.3.1. *Waptia* is a mandibulate

One of the most significant new observations emerging from the present revision of *W. fieldensis* is the presence of a pair of well-developed mandibles. These mandibles are inserted posterior to the antennules and are the second pair of anterior appendages. Their enlarged gnathal segment and three-segmented palp are remarkably similar to those of mandibles in modern crustaceans and suggest a masticatory and sensory (palp) function in relation to feeding. The presence of mandibles is by definition a diagnostic feature of Mandibulata and one of the key synapomorphies of the group [9,131,132]. Mandibulates generally have at least two differentiated post-mandibular appendages, the maxillule and the maxilla (or first and second maxillae), which delimit the head tagma posteriorly and whose exopods and endopods are highly reduced in terrestrial taxa. In Cephalocarida [133] as well as in certain adult [126] and larval (e.g. [82,98,134]) Palaeozoic crustaceomorph taxa, the appendage equivalent to the maxilla retains a morphology similar to that of posterior limbs, while the maxillule shows degrees of differentiation.

We might have a similar configuration in *Waptia* in which the first pair of post-maxillular appendages (pma1) has particularly strong endites and a shorter size, marking a difference with pma2–4. Considering pma1 as a maxilla in the mandibulate sense would thus place the posterior boundary of the head tagma just behind pma1. In this case, *Waptia* would match more closely the mandibulate groundplan of a five-segmented head displayed in extant representatives of the group. However, pma1–4 also form a cohesive functional unit with very similar morphologies and the presence of long basipod endites, and the separation of pma1 as a maxilla is ambiguous from that perspective.

#### 6.3.2. Possible implications for the origin of the mandibulate body plan

With our updated dataset, the very base of Mandibulata is now represented by *Tanazios* [125] and the somewhat less ambiguous and controversial *Cascolus* [124], both from the Herefordshire Lagerstätte. *Cascolus* may offer an interesting perspective on the origin of Mandibulata if the position of hymenocarines as pancrustaceans *sensu lato* is to be confirmed. Although the taxon is Silurian and probably has a number of autapomorphic character states, the very elusive origin of mandibulates invites one to test this hypothesis.

In this taxon, the head tagma bears a shield and the anteriormost appendages are tripartite antennules not dissimilar to the morphology known in *Oelandocaris* [135] or (other) leanchoiliid larvae [134]. Owing to its minute size, the *Cascolus* morphotype could also be immature, and thereby would reinforce the idea that early mandibulate features arose through the heterochronic selection of ontogenetic adaptations in more basal taxa [9].

When compared with protocaridids and *Waptia* in which mandibles are already arguably derived—i.e. with large and differentiated gnathal edges and reduction of the endopods into palps (when present)—*Cascolus* bears mandibular appendages that are still biramous, even if endopods and exopods are already modified and probably have reduced numbers of podomeres. It does not seem possible to determine whether the gnathal element of the mandible belongs to the basipod or a prebasal coxal podomere, as the latter case would be expected for a true mandible (e.g. [92,136]). In other words, it is still unclear whether *Cascolus* (like *Tanazios*) is a crown-group mandibulate, or if it sports an intermediary form of appendage homologous to the mandibles but still based on basipod mastication—as in arachnomorphs [35,137].

In *Cascolus*, appendages that would be equivalent to maxillule and maxilla appear little if at all different from trunk appendages. This supports a transition from a megacheiran type of body plan into a mandibulate one, in which a fifth segment would be incorporated in the head tagma.

Interestingly, *Cascolus* also has a post-antennular pair of appendages, modified into an apparatus both curving around the base of the antennules and hanging over the mouth. Such appendage resembles the post-antennular appendage of fuxianhuiids [70,138]. In the phylogenetic context considered here (figure 27), the post-antennular appendage (i.e. the antenna) therefore would have been lost three times during the evolution of mandibulates: in the myriapod crown, in hymenocarines (at least in part) and in hexapods. Given the putatively strong influence of terrestrialization on the loss of this appendage in myriapods and hexapods (associated with the closure of the head capsule), its reduction in the marine hymenocarines is intriguing. However, ‘odaraiids’ (*Odaraia*, *Nereocaris* [54,139]), which do not seem to express either antennules or antennae, testify to the unusual variability of hymenocarine anterior somites.

Another crustaceomorph fossil from the Herefordshire biota, *Aquilonifer* [140], which was originally interpreted as a very early mandibulate, is here retrieved with *Marrella* and pycnogonids as a sister group to Arachnomorpha (electronic supplementary material, S21). Although the grouping of pycnogonids with any of these taxa is highly unexpected, the presence of seemingly large chelate appendages at the front of *Aquilonifer* triggers provocative questions regarding the identity of the chelifores and the alignment of the arachnomorph head. Those are, however, outside the scope of this study.

#### 6.3.3. Is *Waptia* a pancrustacean?

As already noted by Strausfeld [15], the body plan of *W. fieldensis* differs from that of extant crustaceans in lacking a second pair of antennae (A2) which normally arises between the antennules (A1) and the mandibles. The absence of A2 is a characteristic feature shared by hexapods and myriapods which, in place of the antennal segment of crustaceans, have a so-called intercalary segment expressed during early developmental stages [141–143]. This condition also characterizes protocaridids, and possibly other hymenocarines [9].

*Waptia* and similar hymenocarines are therefore not crown crustaceans. However, with crustaceans being commonly retrieved as a grade leading to hexapods (e.g. [32,33]), the relevant question is whether the presence of antennae necessarily defines the ancestral condition of Pancrustacea. It may seem a rather semantic issue, given that the most speciose pancrustacean lineage—Hexapoda—has lost the antenna, which gives little absolute weight to this character in the characterization of the group. Nonetheless, hexapods being terrestrial (adults, at least) and extremely derived, they are of little help in deciding the acquisition of synapomorphies among marine fossil species at the origin of the group. As we will see, the definition of Pancrustacea is very difficult in the broader context of Mandibulata when marine stem groups are taken into account. In an attempt to understand the position and significance of *Waptia* and hymenocarines relative to Pancrustacea, a number of other characters need to be highlighted:

*Number of endopod podomeres.* Despite the lack of antennae, *Waptia* displays cephalic appendages that seem to illustrate the reduction of cephalic endopods to a five-segmented condition, a diagnostic feature of the pancrustacean ground pattern [95]. In protocaridids, post-cephalic endopods are made of seven podomeres, but the morphology of post-mandibular cephalic appendages is not well known. This opens the possibility that the five-segmented cephalic endopods of *Waptia* are common among hymenocarines—which would be consistent with our tree topology (figure 27). The morphology of the cephalic endopods could therefore directly support the inclusion of hymenocarines within Pancrustacea, should this character be considered a crown-group synapomorphy. Unfortunately, and similar to the antennae, because of the highly derived condition of cephalic endopods in myriapods, it is not possible to polarize the five-segmented state as either diagnostic of Pancrustacea or instead of Mandibulata. This can only be solved by determining the ancestral myriapod condition, potentially by further investigations of euthycarcinoids and fuxianhuiids.

*Structure of the eyes.* Pancrustacea is otherwise synonymous with Tetraconata, a term based on the diagnostic presence of a tetrapartite crystalline cone forming the ommatidia in these taxa. However, this would be a hard character to document in fossils, but the preservation of ommatidia in *Waptia* opens the possibility of investigating crystalline microstructures and determining the state of this character in hymenocarines in the near future.

*Antennal coxa.* The other character optimized as supporting the monophyly of crustaceans and hexapods is the presence of a developed ‘coxa on the post-antennular appendage’ (char. 99). A coxa in the mandibulate sense is an additional podomere proximal to the basipod (i.e. ‘pre-basal’). A prebasal podomere generally characterizes crustaceans [142] and can also be found in a crustaceomorph larva such as *Rehbachiella* [82]. Evidently, this does not apply to hexapods or known hymenocarines either because they lack appendages on this somite.

*Subdivided, enditic basipods.* There are also new characters arising from the description of fossils which could provide another definition of Pancrustacea. One is the presence of subdivided, enditic basipods. This trait was recently argued to be a possible plesiomorphic condition of Mandibulata that would have allowed for the differentiation of coxal elements [9], following a previous evolutionary hypothesis [82,143]. A better constraint on this character at the base of Mandibulata is needed, either through fossils or by investigating whether myriapod pleurites derive from coxal and sub-coxal elements [144], possibly identifiable with multipartite basipods.

*Labrum.* Another character would be the labrum and related sclerotic elements. As discussed above, it is proposed, in the absence of other structures covering the mouth, that the ocular sclerite and associated soft structures in *Canadaspis* [50] protocaridids [9] and *Waptia* may be related to the developmental labrum of crustaceans. This feature (anterior sclerite and underlying tissues), however, is documented as being much more ancestral [58,145], and would plesiomorphically also be present in myriapods.

#### 6.3.4. The hymenocarine head problem

As mentioned in §6.3.1, a fundamental problem pertaining to the correct placement of hymenocarines among Mandibulata is the correct characterization of the head tagma. The mandibulate head is considered to be universally five-segmented [95,131,142]. Unless they have a unique autapomorphic condition, if hymenocarines are considered sister taxa to pancrustaceans, they are thus also mandibulates, and must have five-segmented heads. Bound by the insertion of the maxillipeds, this has been interpreted as such following the description of *Tokummia* and the revision of *Branchiocaris* [9], but post-mandibular appendages were otherwise poorly preserved in these species, and head anatomy has remained enigmatic for other hymenocarines.

Three main interpretations of the cephalon of *Waptia* are possible:

1. The ‘head’ could comprise all anterior appendages up to pm4, and thus include as many as eight segments (including the intercalary segment). This configuration would fall outside the mandibulate definition—and any known definition of head tagmata in euarthropods. We do not see any strong argument to support this interpretation, especially considering comparable models of tagmatization in malacostracans.
2. Only the head section up to the maxillules could be considered as the head tagma, this four-segmented configuration providing *Waptia* with a typical ground-pattern cephalon, as in megacheirans (e.g. [146]) and most artiopods [37,95,137,145,146]. This would favour a more basal position of *Waptia* with respect to the entire Mandibulata.
3. The developmental equivalent of the maxilla is pma1, implying a smooth morphological transition from cephalic to post-cephalic appendages, that is, a relatively little-developed differentiation of the maxilla. This hypothesis works well in conjunction with evidence from other fossils and extant taxa that the plesiomorphic maxilla was—as expected—undifferentiated from more posterior appendages. Although the polarization of potentially pancrustacean synapomorphies is made difficult by the lack of knowledge on the ancestral myriapod body plan (see §6.3.3), our current phylogenetic hypothesis suggests that euthycarcinoids and fuxianhuiids may provide information on the myriapod common ancestor. Relative to these taxa, characters such as body tagmatization, mandibular palps and five-segmented cephalic endopods do suggest a closer affinity to pancrustaceans, and therefore it seems more parsimonious at this time to consider *Waptia* as a pancrustacean with a maxilla undifferentiated from more posterior appendages.

The phylogenetic inclusion of *Waptia* therefore probably leads to an expansion of the definition of Pancrustacea/Tetraconata, but renders the definition of the crustaceans + Hexapoda clade difficult. With a posterior probability of 0.26, this arrangement should be considered tentative. However, given that the reappraisal of hymenocarine taxa is a very recent endeavour, we may expect to refine the diagnostic features of these fossil groups as we gain insight into their anatomy and morphology.

## 7. Lifestyles

### 7.1. Locomotion

**Figure 28.**
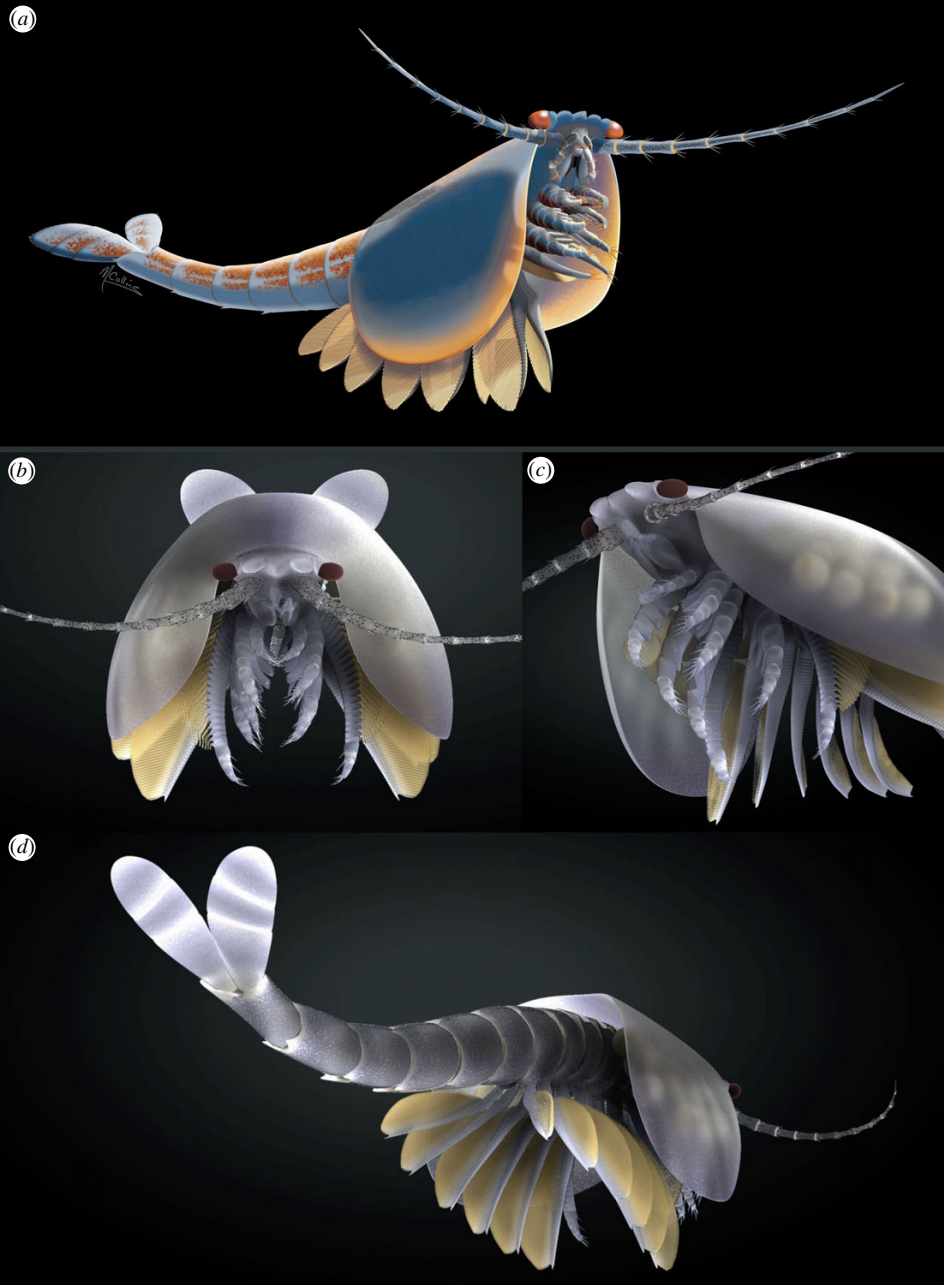
*Waptia fieldensis* Walcott, 1912 [10] from the middle Cambrian (Series 3, Stage 5) Burgess Shale, British Columbia, Canada. (*a*) Artistic reconstruction (drawing by Marianne Collins). (*b–d*) Images from videos (movie by Lars Fields; see electronic supplementary material, S23, S24); frontal and intermediate ventral views showing anterior appendages and intermediate posterior view showing lamellate appendages.

The uniramous post-mandibular appendages of *W. fieldensis* have recently been interpreted as walking legs on the basis of their robust morphology [15]. However, we think that they lack the characteristics of walking appendages: (i) they are thinner and shorter than those of typical epibenthic arthropods such as artiopodans (e.g. *Sidneyia inexpectans*; see [105,147]), or even nektobenthic taxa such as *Yawunik* (Cheiromorpha: Leanchoiliidae [37]) or *Tokummia* (Mandibulata: Protocarididae [9]); (ii) they are inserted along the anterior fourth of the animal's body, in an off-centre position that rules out a possible function as crawling limbs (figures 20 and 28). In fact, all evidence indicates that the first to third (possibly fourth) pairs of post-maxillular appendages were primarily involved in predation and handling of food (figure 28). This is based on their commonly preserved life position, showing them pointing forwards and towards the mouth region, their very elongate pair of distal claws; their exceptionally well-developed endites forming a tight masticatory apparatus only when the limbs are projecting forward in the mandibular area. By contrast, the lamellate post-cephalothoracic appendages with a large area of contact with water were probably dedicated to swimming.

As a result, we think that *W. fieldensis* was neither an epibenthic crawler nor an arthropod with burrowing habits as suggested by Strausfeld [15]. It has been suggested [148] that *W. fieldensis* was a swimming nektobenthic arthropod occasionally clinging to substrates. Indeed, in spite of a primary raptorial/masticatory function, the long claws which terminate the four post-maxillular appendages could have been well adapted for clinging to submarine reliefs and/or sessile and erected organisms such as sponges which were abundant in the Burgess Shale biota [149,150]. They may have offered the animal a firm off-centred grip counterbalanced by the long posterior part of the body, a configuration reminiscent of modern dragonflies.

Swimming most probably resulted from the rowing synchronized movement of the lamellate appendages (figures 20 and 28; videos in electronic supplementary material, S23 and S24) as described in numerous extant crustaceans (e.g. *Gnathophausia* [151]). At the beginning of the power stroke, the annulated shaft rotated forward to form a right angle with the sagittal plane, thus deploying a large lamellate surface against water. Then, appendages flexed back to their initial position during recovery. In contrast with a solid paddle, the lamellate structure, by letting water flow through the lamellar interspace, is likely to have reduced the drag effect on the return stroke, thereby increasing swimming efficiency. The smooth valves of the carapace probably minimized water friction.

Although the post-cephalothoracic appendages of *W. fieldensis* have no exact equivalent among pancrustaceans, this swimming mode is broadly analogous to most extant nektobenthic to pelagic taxa, which use all or some of their post-cephalothoracic appendages for propulsion. *Nebalia*, for instance, has a thin and flexible streamlined carapace comparable with that of *W. fieldensis* and swims using the beating of setose pleopods attached to its abdominal segments [103]. Swimming in *Gnathophausia ingens* is achieved by the oar-like movement of eight post-cephalothoracic exopods and five pleopods [151]. *Waptia fieldensis* probably used its long and flexible abdomen ending with a caudal fan for upward and downward movement within the water column and also to maintain its directional stability. The caudal rami formed two solid paddles lying almost perpendicular to the sagittal plane (figures 20 and 28). Caudal strokes were then able to generate high resistance against water and locomotory power. The rami were articulated with the last abdominal segment and could spread out or fold back by slightly overlapping each other and thus could reduce resistance to forward movement and control thrust.

In summary, *W. fieldensis* was an arthropod with a flexible body, probably a relatively fast swimmer with the capacity to perform and control a wide range of movements within the water column (electronic supplementary material, S23, 24). It may have alternated swimming with resting phases on possibly mineral or biological submarine reliefs. The detailed topography of the seafloor where the Burgess Shale animals lived remains unknown, except for the fact that the nearby Cathedral Escarpment [152] rose above seemingly uniform soft bottoms. Strong interactions with the substrate such as crawling or burrowing are thereby excluded.

### 7.2. Feeding

*Waptia fieldensis* had a pair of mandibles with an enlarged gnathal part and a three-segmented palp bearing numerous setae. Their morphology and arrangement (strongly sclerotized margins converging towards the mouth) suggest that they had the same function as those of many extant pancrustaceans—i.e. that of seizing, cutting, tearing and macerating food before ingestion through the mouth. The lack of sharp marginal teeth and the presence, instead, of molar nodes would indicate a mode of function close to that of branchiopod mandibles. The specialization of the maxillule relative to more posterior limbs, with a distal ornamentation recalling the very setose margin of the mandibular palps and the long claws of post-maxillular appendages, suggests that it was mainly used for sensing and manipulating food—a likely complement to the short mandibular palps (figure 21). As previously mentioned, the following three (possibly four) pairs of post-maxillular appendages would have been well equipped to grab and hold prey items as well as to masticate food with their strong endites (figure 28; videos in electronic supplementary material, S23, 24), The fact that they were not projecting beyond the front of the animal implies that *W. fieldensis* had to catch its prey by wrapping it under its head. Alternatively, it could have used its long endites to either grate the surface of sponges or carcasses or perhaps to dig out prey items buried in soft mud. The exact diet of *W. fieldensis* remains unknown because of the lack of direct information from gut contents (e.g. [105] for *Sidneyia* from the Burgess Shale). However, because of the lack of undigested hard elements in its gut, *W. fieldensis* may have been feeding on soft prey or soft tissues from carcasses.

### 7.3. Sensing

Strausfeld [15] ascribed to *W. fieldensis* a wide range of sensory features encompassing visual organs (stalked eyes, median eye comparable with ocelli) and mechano- and chemoreceptors (e.g. aesthetacts) distributed on various parts of the animal (antennules, anterior appendages, posterior segments, caudal rami). We have pointed out above the limits of these interpretations, and will only discuss here features that are strongly supported by fossil evidence.

*Waptia fieldensis* had stalked eyes with probably several hundreds of ommatidia distributed over a hemi-elliptical visual surface (figure 28; videos in electronic supplementary material, S23 and S24). It had a frontal and lateral field of vision. The assumed deutocerebrum of *W. fieldensis* (figure 25) received input from antennules via sensory antennal nerves connected to numerous bunches of setae. The exact function of these sensory setae is however uncertain. In extant crustaceans, the deutocerebrum has an important olfactory function and receives signals from the innervating aesthetascs of antennules. The presence of true aesthetascs with an assumed olfactory function cannot be confirmed in *Waptia*. On the other hand, the paired inter-ocular lobes are good candidates for the location of hemi-ellipsoid bodies, which are olfactory neuropils.

## 8. Conclusion

The detailed study of *W. fieldensis* presented here leads to the most complete reconstruction of this iconic Burgess Shale animal discovered by Charles Walcott in 1909. This account of *Waptia* was, in part, made possible by the remarkable new material collected by the Royal Ontario Museum. The unprecedented fine characterization of key anatomical features, and in particular of mandibles and other features with mandibulate affinities, has allowed for an in-depth reevaluation of the significance of *Waptia* for the early evolution of euarthropods. Our interpretation of the anterior limb morphology contributes to place *Waptia* and its hymenocarine relatives within a more broadly defined Pancrustacea (figure 27). Nevertheless, *Waptia* also illustrates the unusual characteristics of the hymenocarine body plan, supporting the reduction of the post-antennular appendages (as an intercalary segment) in certain forms, and presenting posterior limbs with a very atypical shape. *Waptia* also exemplifies the challenge of aligning the hymenocarine head tagma with other euarthropods based on external morphology alone. The investigation of other related taxa will be essential to the understanding of the pivotal position of Hymenocarina in the early radiation of mandibulates [9].

SCFs have become an important source of information on the early evolution of ecdysozoans and other animal groups, complementary to that provided by Burgess Shale-type and Orsten-type fossils. These flattened carbonaceous remains extracted from rocks reveal extremely fine morphological details of appendages such as filtering apparatuses and mandibles often similar to those of modern crustaceans such as ostracods, copepods and branchiopods (e.g. middle and late Cambrian SCF assemblages from Canada [94,153]). Although it remains difficult to ascertain whether these isolated elements truly belong to derived crown pancrustaceans, the evidence presented here partly sheds light on the significance of crustaceomorph SCFs by showing with body macrofossils that pancrustaceans were possibly already well diversified by the Cambrian Series 2. *Waptia fieldensis* provides complementary evidence that some middle Cambrian shrimp-like basal euarthropods had a full array of complex anatomical features related to food detection, handling and processing as exemplified by palp-bearing mandibles. Together, these results highlight that adaptations to complex food handling appeared relatively early in the evolution of mandibulates and played an important role in the ecological diversification of the group.

## Supplementary Material

ESM S1-S20,ESM 22

## Supplementary Material

ESM S21

## Supplementary Material

ESM S23

## Supplementary Material

ESM S24

## Data accessibility

The datasets supporting this article have been uploaded as part of the electronic supplementary material.
