# Supplementary material for: Waptia fieldensis Walcott, a mandibulate arthropod from the middle Cambrian Burgess Shale
Source: R Soc Open Sci. 2018 Jun 20;5(6):172206. doi: 10.1098/rsos.172206 (PMC6030330; doi:10.1098/rsos.172206)
Supplement: ESM S1-S20,ESM 22 [file rsos172206supp1.pdf]

***Waptia fieldensis* Walcott, a mandibulate arthropod from the middle Cambrian Burgess Shale**

Jean Vannier, Cédric Aria, Rod S. Taylor and Jean-Bernard Caron

Electronic supplementary material S1-S20, 22

S1- *W. fieldensis*, geographic and stratigraphic distribution.
S2- *Nebalia bipes* (Phyllocarida), decay experiments.
S3- *W. fieldensis*, fossil assemblage.
S4- *W. fieldensis*, synonymy list.
S5- *W. fieldensis*, type specimens.
S6- *W. fieldensis*, measurements (1).
S7- *Nebalia bipes* (Phyllocarida), morphology.
S8- *W. fieldensis*, frontal part.
S9- *Vargula hilgendorfii* (Ostracoda), labrum.
S10- *Cirolana harfordi* (Isopoda), mandibles.
S11- *W. fieldensis*, mandibles and maxillules.
S12- *W. fieldensis*, post-maxillular cephalothoracic appendages.
S13- *W. fieldensis*, post-maxillular cephalothoracic appendages (frontal view).
S14- *W. fieldensis*, lamellate appendages.
S15- *Crangon crangon* (Decapoda), gills.
S16- *W. fieldensis*, measurements (2).
S17- *W. fieldensis*, digestive system (1).
S18- *W. fieldensis*, digestive system (2).
S19- Cladogram (adult taxa).
S20- List of characters.
S22- Cladogram (larval and adult taxa).

S21- Matrix of characters used in the present cladistic analysis (IN SEPARATE FILE)

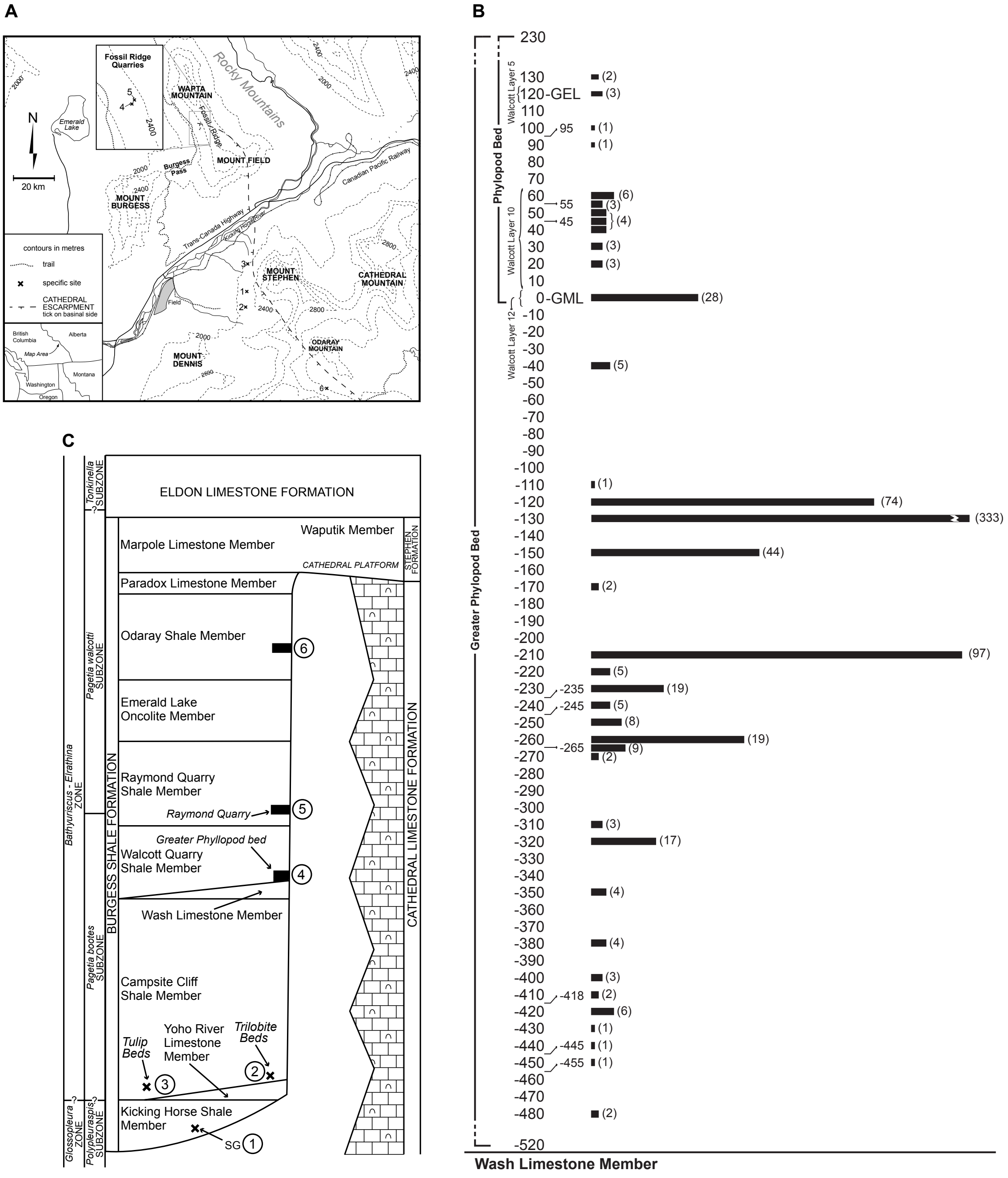

Electronic supplementary material S1. *Waptia fieldensis* Walcott, 1912 from the middle Cambrian (Series 3, Stage 5) Burgess Shale, British Columbia, Canada; geographic and stratigraphic distribution. (*a*) Locality map; 1, SG locality, Mount Stephen; 2, ST locality, Trilobite Beds; 3, Tulip Beds (S7) locality; 4, Walcott Quarry (Greater Phyllopod Bed); 5, Raymond Quarry. (*b*) Detailed vertical distribution within the Greater Phyllopod Bed; levels in cm above and below the base of the Walcott Quarry; GEL, Great *Eldonia* Layer; GML; Great *Marrella* Layer. (*c*) Stratigraphic occurrences of *Waptia fieldensis* within the Burgess Shale ("Thick" Stephen) Formation.

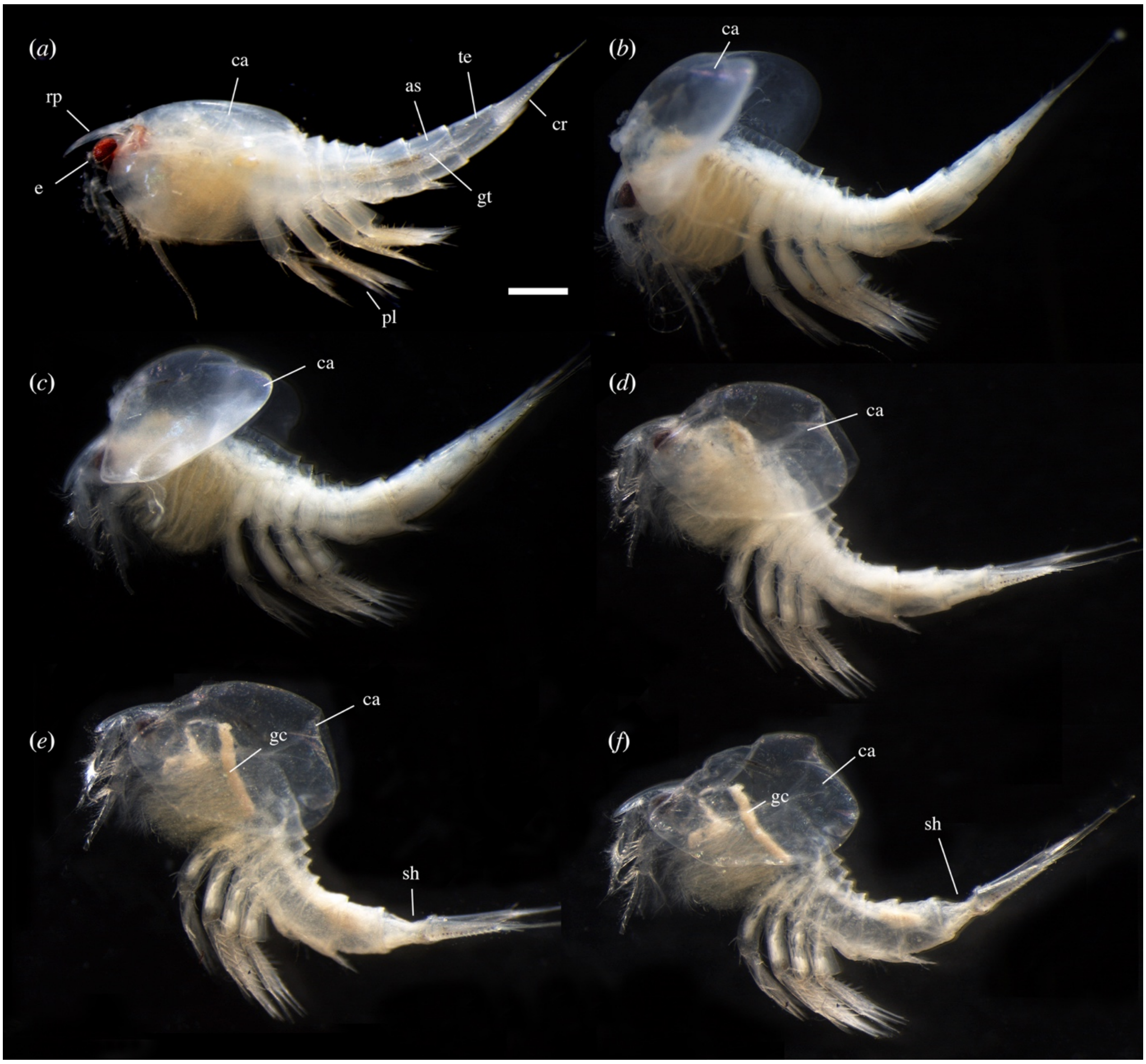

Electronic supplementary material S2. *Nebalia bipes* (Crustacea, Malacostraca, Leptostraca); decay experiments. (*a-f*) Live specimen in lateral view and 2, 4, 8, 14 and 21 days after death (kept in a petri dish filled with sea water, no sediment, temperature 20°C). Abbreviations are as follows: as, abdominal segment; ca, carapace; cr, caudal ramus; e, eye; gc, gut content; gu, gut; pl, pleopod; rp, rostral plate; sh, shrinkage; te, telson. Scale bar: 1 mm.

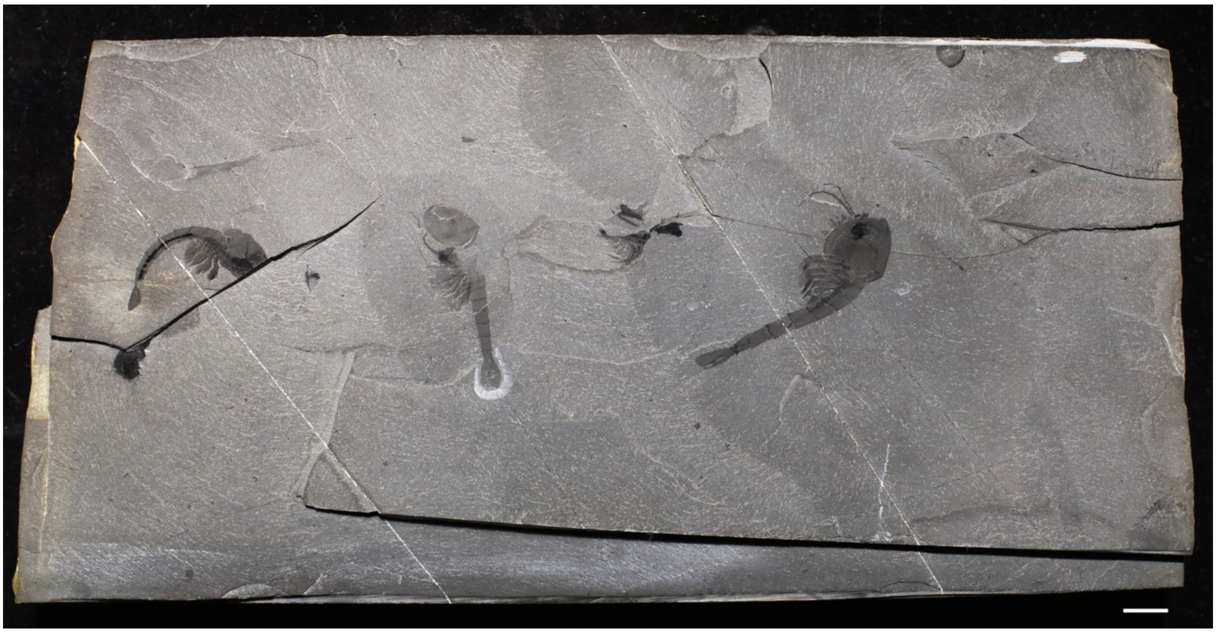

Electronic supplementary material S3. *Waptia fieldensis* Walcott, 1912 from the middle Cambrian (Series 3, Stage 5) Burgess Shale, British Columbia, Canada; rock slab (ROMIP 56421) from level -120 cm (Greater Phyllopod Bed) showing three complete specimens on the same bedding plane. Note the specimen in the middle shows clear sign of disarticulation of the carapace. Photograph taken under cross-polarized light. Scale bar: 1 cm**.**

Electronic supplementary material S4. *Waptia fieldensis* Walcott, 1912 from the middle Cambrian (Series 3, Stage 5) Burgess Shale, British Columbia, Canada; Synonymy list.

Waptia fieldensis Walcott, 1912

1912 *Waptia fieldensis*; Walcott [1], p. 152-4, 156-9, 161, 163, 181-182, 214-215, 220-221; Pl. 27, figs 4, 5.
1912 *Burgessia bella*; Walcott [1]; p. 177, 180; Pl. 30, fig. 4 (USNM 57679, erroneously recorded as USNM 57680).
1919 *Waptia*; Crampton [2], p. 155-156.
1920 *Waptia fieldensis*; Raymond [3], p. 108-109.
1925 *Waptia fieldensis*; Fedotov [4], p. 386, 389.
1928 *Waptia fieldensis*; Henriksen [5], p. 2, 10, 13-14.
1930 *Waptia*; Beurlen [6], p. 501.
1931 *Waptia fieldensis*; Walcott ([7], posthumous), p. 20-24, 43-44; figs 6-7. (reconstructions); Pl. 18, figs 2-5, Pl. 19, figs 1-4; Pl. 20, figs 1-3; Pl. 21, fig. 2.
1931 *Waptia circularis*; Walcott ([7], posthumous), p. 24, 45; Pl. 21, fig.3.
1933 *Waptia*; Størmer [8], p. 154, 156; fig. 2c.
1935 *Waptia*; Raymond [9], p. 214-217, 227.
1939 *Waptia fieldensis*; Størmer [10], p. 230-232, 233, 237, 267-268; figs 29g, 30d.
1944 *Waptia fieldensis*; Størmer [11], p. 87, 94, 95, 97, 99-101, 106, 107, 109, 113, 123, 124, 130; fig 19, 7-8.
1946 *Waptia*; Linder [12], p. 20-21.
1949 *Waptia*; Vandel [13], p. 88, 140.
1953 *Waptia*; Dechaseaux [14], p. 37-39, 43; fig. 11.
1953 *Waptia fieldensis*; Heldt [15], p. 177-180; Pl. 21, figs 2-4.
1954 *Waptia*; Burton [16], p. 108, 125, 126; fig. 45.
1956 *Waptia*; Caster and Brooks [17], p. 180.
1958 *Waptia fieldensis*; Tiegs and Manton [18], p. 291-293, 313-314, 316; fig. 6c.
1959 *Waptia*; Størmer [19], p. O23-28, O32, O33 (Treatise on Invertebrate Paleontology); fig. 21, 6-8.
1960 *Waptia fieldensis*; Novozhilov [20], p. 198, 199, 207; fig. 441.
1962 *Waptia fieldensis*; Rolfe [21], p. 5.
1970 *Burgessia bella*; Simonetta [22], Pl. 28, fig. 4.
1974 *Waptia fieldensis*; Whittington [23], p. 15, 19-20; Pl. 27, figs 3, 4.
1975 *Waptia*; Hessler and Newman [24], p. 448-449; fig. 10B.
1975 *Waptia fieldensis*; Hughes [25], p. 415, 418, 419.
1975 *Waptia fieldensis*, Simonetta and Delle Cave [26], p. 10, 34; Pl; 5, figs 5a, b (reconstructions); Pl. 41, figs 1-11; Pl. 42, figs 1-15; Pl. 43, figs 1-7.
1977 *Waptia fieldensis*; Hughes [27], p. 15.
1979 *Waptia*; Conway Morris and Whittington [28], p. 111.
1979 *Waptia fieldensis*; Doveton [29], p. 215-226; Pl. 1, figs 2, 4-5.
1980 *Waptia fieldensis*; Tasch [30], p. 482-485, 491; fig. 10.4F.
1983 *Waptia*; McKenzie [31], p. 36 ; table 1.
1980 *Waptia fieldensis*; Bergström [32], p. 10.
1982 *Waptia fieldensis*; Hughes *in* Conway Morris *et al.* [33], p. 18; fig. K (lectotype, left specimen).
1982 *Waptia*; Whittington [34], p. 19; figs 2-10.
1983 *Waptia fieldensis*; Briggs [35], p. 5-6, 12, 15-18; figs 2A, 3, 4; table 2.
1984 *Waptia*; Dahl [36], p. 68, 74.
1985 *Waptia fieldensis*; Briggs and Whittington [37], p. 150, 152, 155-156, 158; fig. 2i; table 1.
1985 *Waptia fieldensis*; Whittington [38], p. 45, 65-66, 68, 104, 123, 138; figs 4.51, 5.1.
1986 *Waptia*; Conway Morris [39], p. 439; fig. 7c.
1986 ?*Waptia*; Schram [40], p. 33; table 2-2.
1989 *Waptia*; Briggs and Fortey [41], p. 242; fig. 1.
1989 *Waptia*; Gould [42], p. 25, 72-73, 121, 138, 219, 221; figs 2.6, 3.7.
1990 *Waptia*; Briggs [43], p. 25, 33, 34; fig. 3.
1991 *Waptia*; Delle Cave and Simonetta [44], p. 190, 228, 229; fig. 25D1-2.
1992 *Waptia*; Bergström [45], p. 288, 289, 290; figs 2, 3.
1992 *Waptia*; Briggs *et al.* [46], p. 1671, 1672; figs 1-3.
1993 *Waptia*; Briggs *et al.* [47], p. 39-42; figs 1,2; tables 1, 2.

1994 *Waptia fieldensis*; Briggs *et al*. [48], p. 157, 158, figs 110-112 (reconstruction).
1994 *Waptia*; Wills *et al*. [49], p. 106, 109, 111, 115; figs 4, 7, 8, 11.
1995 *Waptia*; Bousfield [50], p. 24.
1997 *Waptia fieldensis*; Chen and Zhou [51], p. 67-69.
1997 *Waptia*; Erwin *et al*. [52], p. 128-129; fig. 2.
1997 *Waptia fieldensis*; Hou and Bergström [53], p. 41-42, 105, 111.
1997 *Waptia*; Wills [54], p. 197, 198, 201; fig. 15.4.
1997 *Waptia*; Wills *et al*. [55], p. 59, 60, 63; figs 6.1, 6.2, 6.5.
1998 *Waptia*; Conway Morris [56], p. 173, 174, 195, 288-291; figs 6.8-6.11.
1998 *Waptia*; Schram and Hof [57], p. 238-240, 265, 283, 284; fig. 6.3; table 6.3 (p. 283, cladistic analysis).
1998 *Waptia*; Fletcher and Collins [58], p. 428.
1998 *Waptia*; Wills *et al*. [59], p. 46, 71-74, 80, 83, 84, 86, 87; figs 2.1(d), 2.2, 2.3, 2.5, 2.6, 2.7; table 2.1 (cladistic analysis).
1999 *Waptia fieldensis*; Waloszek [60], p. 21.
1999 *Waptia fieldensis*; Hou [61], p. 111, 114.
2000 *Waptia fieldensis*; Garcia-Bellido [62], p. 145; fig. 5.
2000 *Waptia*; Bengtson [63], p. 5; fig. 2.
2001 *Waptia*; Budd [64], p. 410; fig. 18.1.
2001 *Waptia fieldensis*; Donovan and Lewis [65], p. 232; fig. 1a.
2002 *Waptia fieldensis*; Taylor [66], p. 113, 117, 120; fig. 11A.
2002 *Waptia fieldensis;* Taylor and Collins [67], abstract.
2004 *Waptia fieldensis;* Hou *et al*. [68], p. 136.
2004 *Waptia*; Schwab [69], p. 164.
2005 *Waptia fieldensis*; Parker [70], p. 2; fig. 1.
2005 *Waptia fieldensis*; Davidson and Erwin [71], p. 797; fig. 1b.
2006 *Waptia fieldensis*; Caron and Jackson [72], p. 456, 458; fig 5; Supplementary Data 5F.
2007 *Waptia fieldensis*; Zhang and Shu [73], p. 1414; fig. 4.6.
2008 *Waptia fieldensis*; Caron and Jackson [74]; figs 11, 12; table 1; Appendix B, C, D, F.
2008 *Waptia fieldensis*; Liu and Shu [75], p. 353, 360.
2008 *Waptia* cf. *fieldensis*; Briggs *et al*. [76]; p. 250, 251; fig. 12.
2009 *Waptia*; Collins [77]; p. 16, 25.
2009 *Waptia*; Hou *et al*. [78]; p. 957, 958.
2009 *Waptia fieldensis*; Caron [79], p. 74; fig. 18; table 1.
2009 *Waptia fieldensis*; Strausfeld [80] (abstract).
2011 *Waptia fieldensis*; Strausfeld [81]; p. 157-167; figs 1-7 (USNM 83948j, erroneously recorded as USNM 57682 in fig. 4; reconstructions in figs 1, 4), table 1.
2012 *Waptia fieldensis*; Strausfeld [82]; p. 588-592, fig. 12.10 (reconstruction).
2012 *Waptia fieldensis*; Vannier *et al*. [83], p. 92 (abstract).
2016 *Waptia fieldensis*; Strausfeld [84]; p. 173-184; figs 1-6 (reconstructions in figs 1, 2, 6); suppl. fig. 1.
2016 *Waptia fieldensis*; Caron and Vannier [85], p. 1-6; figs 1, 2, 3A; suppl. figs 1-3.

References

1. Walcott CD. 1912 Middle Cambrian Branchiopoda, Malacostraca, Trilobita and Merostomata. Cambrian Geology and Paleontology II. *Smithsonian Miscellaneous Collections* **57**, 145-228.
2. Crampton GC. 1919 The evolution of arthropods and their relatives with special reference to insects. A*merican Naturalist* **53**, 143-179.
3. Raymond PE. 1920 The appendages, anatomy and relationships of trilobites. *Memoirs of the Connecticut Academy of Arts and Sciences* **7**, 1-169.
4. Fedotov, D. 1925 On the relations between the Crustacea, Trilobita, Merostomata and Arachnida. *Bulletin de l'Académie des Sciences de Russie* **18**, 383-408.
5. Henriksen KL. 1928 Critical notes upon some Cambrian arthropods described by Charles D. Walcott. *Videnskabelige Meddelelser fra Dansk naturhistorisk Forening i Kjøbenhavn* **86**, 1-20.
6. Beurlen K. 1930 Vergleichende Stammesgeschichte, Grundlagen, Methoden und Probleme unter besonderer Berücksichtigung der hoheren Krebsen. *Fortschritte der Geologie und Paläontologie* **8**, 314-586.
7. Walcott CD. 1931 Addenda to description of Burgess Shale fossils [with explanatory notes by Charles E. Resser]. Smithsonian Miscellaneous Collections **85**, 1-46.
8. Størmer L. 1933 Are the trilobites related to the arachnids? *American Journal of Science* **26**, 147-157.
9. Raymond PE. 1935 *Leanchoilia* and other Mid-Cambrian Arthropoda. *Bulletin of the Museum of Comparative Zoology at Harvard College* **76**, 205-230.

10. Størmer L. 1939 Studies on trilobite morphology, Part 1, the thoracic appendages and their phylogenetic significance. *Norsk geologisk tidsskrift* **19**, 143-273.
11. Størmer L. 1944 On the relationships and phylogeny of fossil and recent Arachnomorpha. *Skrifter utgitt av Det Norske Videnskaps-Akademni Oslo, I Matematisk-naturvidenskapelig klasse* **5**, 1-158.
12. Linder F. 1946 Affinities within the Branchiopoda, with notes on some dubious fossils. *Arkiv för zoology* **37**, 1-28.
13. Vandel A. Généralités et composition de l'embranchement. In *Traité de Zoologie 6 (Onychophores-Tardigrades-Arthropodes-Trilobitomorphes-Chélicérates)* (ed PP Grassé), pp. 79-158. Masson, Paris.
14. Dechaseaux, C. 1953 Classes des mérostomides, des pseudocrustacés et des marrellomorphes. In Traité de Paléontologie, Tome III (ed J Piveteau), pp. 347-406. Masson, Paris.
15. Heldt JH. 1953 *Waptia fieldensis* Walcott et les stades larvaires des Pénéides. *Bulletin de la Société des Sciences Naturelles de Tunisie* **6**, 177-180.
16. Burton J. 1954 Living Fossils. Thames and Hudson, London, 282 p.
17. Caster KE, Brooks HK. 1956 New fossils from the Canadian-Chazyan (Ordovician) hiatus in Tennessee. *Bulletins of American Paleontology* **36**, 157-199.
18. Tiegs OW, Manton SM. 1958 The evolution of the Arthropoda. *Biological Reviews* **33**, 255-337.
19. Størmer L. 1959 Trilobitomorpha. In *Treatise on Invertebrate Paleontology Part O, Arthropoda 1* (ed RC Moore), pp. O22-O37. The University of Kansas Press and the Geological Society of America.
20. Novozhilov NI. 1960 Merostomoidea, Hemicrustacea. In *Fundamentals of Palaeontology - Arthropods, Trilobites and Crustaceans* (ed T Chernysheva), pp. 195-200. State Technical Science Publishing House for Literature on the Geology and Conservation of Mineral Resources, Moscow.
21. Rolfe WDI. 1962 Two new arthropod carapaces from the Burgess Shale (middle Cambrian) of Canada. *Brevoria* **160**, 1-9.
22. Simonetta A. 1970 Studies on non-trilobite arthropods from the Burgess Shale (middle Cambrian). *Palaeontographia Italica* **66** (N.S. 36), 35-45.
23. Whittington HB. 1974 *Yohoia* Walcott and *Plenocaris* n. gen., arthropods from the Burgess Shale, middle Cambrian, British Columbia. *Geological Survey of Canada Bulletin* **231**, 1-27.
24. Hessler RR, Newman WA. 1975 A trilobitomorph origin for the Crustacea. *Fossils and Strata* **4**, 437-459.
25. Hughes CP. 1975 Redescription of *Burgessia bella* from the middle Cambrian Burgess Shale, British Columbia. *Fossils and Strata* **4**, 415-435.
26. Simonetta A., Delle Cave L. 1975 The Cambrian non-trilobite arthropods from the Burgess Shale of British Columbia. A study of their comparative morphology, taxinomy and evolutionary significance. *Palaeontographia Italica* **69**, 1-37.
27. Hughes CP. 1977 The early arthropod *Waptia fieldensis*. *Journal of Paleontology* **51** (supplement to 2), 15.
28. Conway Morris S, Whittington HB. 1979 The animals of the Burgess Shale. *Scientific American* **241**, 110-120.
29. Doveton JH. 1979 Numerical methods for the reconstruction of fossil material in three dimensions. Ge*ological Magazine* **116**, 215-226.
30. Tasch P. 1980 Paleobiology of the Invertebrates. John Wiley and Sons, New York, 654 pp.
31. McKenzie KG. 1983 On the origin of Crustacea. In Papers from the Conference on the Biology and Evolution of Crustacea (ed JK Lowry), Australian Museum Memoir 18, pp. 21-43. Trustees of the Australian Museum, Sidney.
32. Bergström J. 1980 Morphology and systematics of early arthropods. *Abhandlungen des Naturwissenschaftlichen Vereins in Hamburg Series* **23**, 7-42.
33. Conway Morris S, Whittington HB, Briggs DEG, Hughes CP, Bruton DL. 1982 Atlas of the Burgess Shale. Palaeontological Association, London.
34. Whittington HB. 1982 The Burgess Shale fauna and the early evolution of metazoan animals. In *Palaeontology, Essential of Historical Geology* (ed EM Gallitelli), pp. 11-24. S.T.E.M, Mucchi, Italy.
35. Briggs DEG. 1983 Affinities and early evolution of the Crustacea, the evidence of the Cambrian fossils. In *Crustacean Issues 1, Crustacean Phylogeny* (ed FR Schram), pp. 1-22. Balkema, Rotterdam.
36. Conway Morris, S. 1998 The crucible of creation, the Burgess Shale and the rise of animals. Oxford University Press, 242 pp.
37. Dahl E. 1984 The subclass Phyllocarida (Crustacea) and the status of some early fossils, a neontologist's view. *Videnskabelige Meddelelser fra Dansk naturhistorisk Forening i Kjøbenhavn* **145**, 61-76.
38. Briggs DEG, Whittington H. 1985 Modes of life of arthropods from the Burgess Shale, British Columbia *Earth and Environmental Science Transactions of the Royal Society of Edinburgh* **76**, 149-160.
39. Whittington HB. 1985 The Burgess Shale. Yale University Press, New Haven, 387 pp.
40. Conway Morris S. 1986 The community structure of the middle Cambrian phyllopod bed (Burgess Shale). *Palaeontolog*y **29**, 423-467.
41. Schram FR. 1986 Crustacea. Oxford University Press, 606 pp**.**
42. Briggs DEG, Fortey RA. 1989 The early radiation and relationships of the major arthropod groups. *Science* **246**, 241-243.
43. Gould SJ. 1989 Wonderful life, the Burgess Shale and the nature of history. Norton, New York, 347 pp**.**
44. Briggs DEG. 1990 Early arthropods, dampening the Cambrian explosion. In *Arthropod paleobiology* (ed M Mikulic), pp. 24-43. Short Courses in Paleontology 3, Paleontological Society, Kansas.
45. Delle Cave L., Simonetta AM. 1991 Early Palaeozoic arthropods and problems of animal phylogeny; with some notes on taxa of doubtful affinities. In *The early evolution of Metazoa and the significance of problematic taxa* (eds AM Simonetta, S Conway Morris), pp. 189-244. Cambridge University Press, Cambridge, UK.
46. Bergström J. 1992 The oldest arthropods and the origin of the Crustacea. *Acta Zoologica* **73**, 287-291.

47. Briggs DEG, Fortey RA, Wills MA. 1992 Morphological disparity in the Cambrian. *Science* **256,** 1670-1673.
48. Briggs DEG, Fortey RA, Wills MA. 1993 How big was the Cambrian explosion? A taxonomic and morphological comparison of Cambrian and Recent arthropods. In *Evolutionary patterns and processes* (eds DR Lees, D Edwards), pp. 33-44. Linnean Society Symposium Series 14.
49. Briggs DEG, Erwin DH, Collier FJ. 1994 The Fossils of the Burgess Shale. Washington D.C. Smithsonian Institution Press, 238 p.
50. Wills MA, Briggs DEG, Fortey RA. 1994 Disparity as an evolutionary index, a comparison of Cambrian and Recent arthropods. *Paleobiology* **20**, 93-130.
51. Bousfield EL. 1995 A contribution to the natural classification of lower and middle Cambrian arthropods, food-gathering and feeding mechanisms. *Amphipacifica* **2**, 3-34.
52. Chen JY, Zhou GQ. 1997 Biology of the Chengjiang Fauna. In *The Cambrian Explosion and the Fossil Record* (eds JY Chen, HV Iten), pp. 11-105. Bulletin of the National Museum of Natural Science 10, National Museum of Natural Science, Taichung, Taiwan.
53. Erwin D, Valentine J, Jablonski D. 1997 The origin of animal body plans. *American Scientist* **85**, 126-137.
54. Hou XG, Bergström J. 1997 Arthropods of the Lower Cambrian Chengjiang fauna, southwest China. *Fossils and Strata* **45**, 1-116.
55. Wills MA. 1997 A phylogeny of recent and fossil Crustacea derived from morphological characters. In *Arthropod Relationships, Systematics Association Special Volume Series 55* (eds RA Fortey, RH Thomas), pp. 189-209. Chapman and Hall, London.
56. Wills MA, Briggs DEG, Fortey RA. 1997 Evolutionary correlates of arthropod tagmosis, scrambled legs. In *Arthropod Relationships, Systematics Association Special Volume Series 55* (eds RA Fortey, RH Thomas), pp. 57-65. Chapman and Hall, London.
57. Schram FR, Hof CHJ. 1998 Fossils and the interrelationships of major crustacean groups. In Arthropod Fossils and Phylogeny (ed GD Edgecombe), pp. 233-302. Columbia University Press, New York.
58. Fletcher TP, Collins DH. 1998 The middle Cambrian Burgess Shale and its relationship to the Stephen Formation in the southern Canadian Rocky Mountains. *Canadian Journal of Earth Sciences* **35**, 413-436.
59. Wills MA, Briggs DEG, Fortey RA, Wilkinson M, Sneath PHA. 1998. An arthropod phylogeny based on fossil and recent taxa. In Arthropod Fossils and Phylogeny (ed GD Edgecombe), pp. 33-105. Columbia University Press, New York.
60. Waloszek D. 1999 On the Cambrian diversity of Crustacea. In Proceedings of the Fourth International Crustacean Congress, Amsterdam, the Netherlands, 20-24 July 1998. (eds FR Schram, JC von Vaupel Klein), pp. 3-27. Brill, Leiden.
61. Hou XG. 1999 New rare bivalved arthropods from the Lower Cambrian Chengjiang Fauna, Yunnan, China. *Journal of Paleontology* **73**, 102-116.
62. Garcia-Bellido Capdevila D. 2000 The Burgess Shale Fossils at the Natural History Museum, London. *The Geological Curator* **7**, 141-148.
63. Bengtson S. 2000 Teasing fossils out of shales with cameras and computers. *Palaeontographica Electronica* **2000,** 3.
64. Budd GE 2001 Ecology of nontrilobite arthropods and lobopods in the Cambrian. In *The Ecology of the Cambrian Radiation* (eds AY Zhuravlev, R Riding), pp. 404-427. Columbia University Press, New York.
65. Donovan SK, Lewis DN. 2001 Fossils explained, the Burgess Shale biota. *Geology Today* **17**, 231-235.
66. Taylor RS. 2002 A new bivalved arthropod from the Early Cambrian Sirius Passet fauna, North Greenland. *Palaeontology* **45**, 97-123.
67. Taylor RS, Collins DH. 2002 W*aptia fieldensis*, a possible crustacean from the middle Cambrian Burgess Shale of British Columbia, Canada. Palaeontological Association 46th Annual Meeting, Cambridge, 15-18 December, 2002, Abstract Volume.
68. Hou XG, Aldridge RJ, Bergström J, Siveter David J, Siveter Derek J, Feng XH. 2004 The Cambrian fossils of Chengjiang, China, the flowering of early animal life. Blackwell, 233 pp.
69. Schwab IR. 2004 To see what you eat. *British Journal of Opthamology* **88**, 164.
70. Parker AR. 2005 A geological history of reflecting optics. *Journal of the Royal Society Interface* **2**, 1-17.
71. Davidson EH, Erwin DH. 2006 Gene regulatory networks and the evolution of animal body plans. *Science* **311**, 796-800.
72. Caron JB, Jackson DA. 2006 Taphonomy of the Greater Phyllopod Bed Community, Burgess Shale. *Palaios* **21**, 451-465.
73. Zhang XG, Shu DG. 2007 Soft anatomy of sunellid arthropods from the Chengjiang Lagerstätte, lower Cambrian of southwest China. *Journal of Paleontology* **81**, 1412-1422.
74. Caron JB, Jackson DA. 2008. Paleoecology of the Greater Phyllopod Bed Community, Burgess Shale. *Palaeogeography, Palaeoclimatology, Palaeoecology* **258**, 222-256.
75. Liu HQ, Shu DG. 2008 *Chuandianella ovata* from Lower Cambrian Chengjiang biota. *Acta Palaeontologica Sinica* **47**, 352-361.
76. Briggs DEG, Lieberman BS, Hendricks JR, Halgedahl SL, Jarrard RD. 2008 Middle Cambrian arthropods from Utah. *Journal of Paleontology* **82**, 238-254.
77. Collins, D. 2009 A brief history of field research on the Burgess Shale. In *A Burgess Shale primer, history, geology and research highlights; field trip companion volume of the International Conference on the Cambrian Explosion, August 3-8, 2009* (eds JB Caron, D Rudkin), pp. 15-31. Banff, Alberta.
78. Hou XG, Siveter, Derek J, Aldridge RJ, Siveter David J. 2009 A new arthropod in chain-like associations from the Chengjiang Lagerstätte (Lower Cambrian), Yunnan, China. *Palaeontology* **52**, 951-961.

79. Caron JB. 2009 The Greater Phyllopod Bed community, historical variations and quantitative approaches. In *A Burgess Shale primer, history, geology and research highlights; field trip companion volume of the International Conference on the Cambrian Explosion, August 3-8, 2009* (eds JB Caron, D Rudkin), pp. 71-89. Banff, Alberta.
80. Strausfeld NJ. 2009 Fossil arthropods, early brains, inferring cerebral complexity from preserved sensilla. International Conference on the Cambrian Explosion, Banff, Alberta, 3-8 August, 2009, Abstract Volume.
81. Strausfeld NJ. 2011 Some observations on the sensory organization of the crustaceomorph *Waptia fieldensis* Walcott. *Palaeontographica Canadiana* **31**, 157-158.
82. Strausfeld NJ. 2012 Arthropod brains, evolution, functional elegance, and historical significance. Belknap, Harvard, 830 pp.
83. Vannier J, Yang XF, Lerosey-Aubril R, Legg D. 2012 *Waptia*, a forgotten Burgess Shale arthropod revisited. 56th Annual Meeting of the Palaeontological Association, Dublin, 16-18 December 2012, Abstract Volume.
84. Strausfeld NJ. 2016 *Waptia* revisited, intimations of behaviours. *Arthropod Structure and Development* **45**, 173-184.
85. Caron JB, Vannier J. 2016 *Waptia* and the diversification of brood care in early arthropods. *Current Biology* **26**, 1-6.

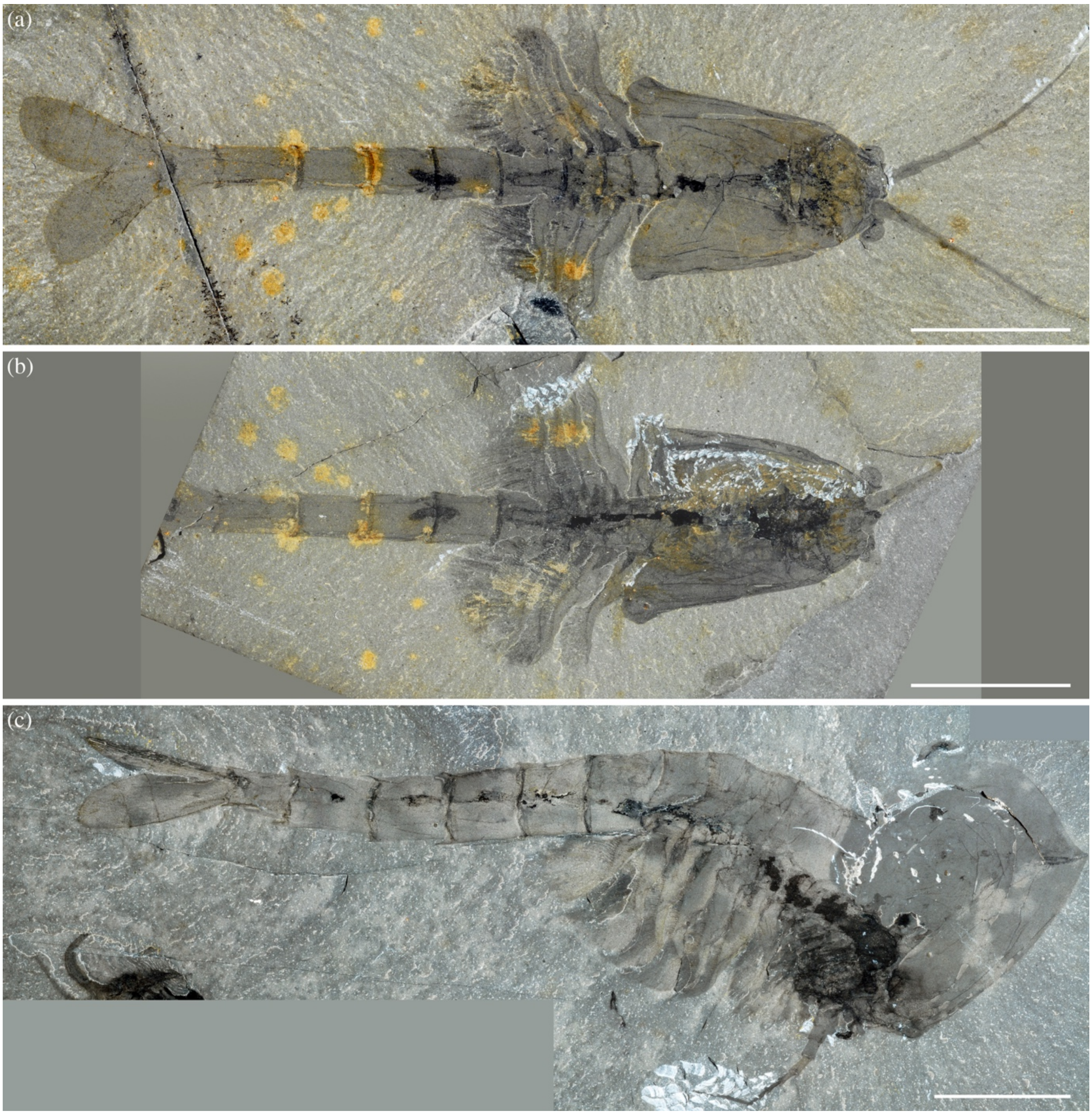

Electronic supplementary material S5. *Waptia fieldensis* Walcott, 1912 from the middle Cambrian (Series 3, Stage 5) Burgess Shale, British Columbia, Canada; type specimens. (*a, b*) USNM 57681, lectotype (designated by Hughes *in* Conway Morris (1982), part and counterpart, preserved in ventral view, post-antennular anterior appendages missing. (*c*) USNM 57682, preserved in lateral view, carapace displaced forwards. Scale bar: 1 cm.

Reference: Conway Morris S, Whittington HB, Briggs DEG, Hughes CP, Bruton DL. 1982 Atlas of the Burgess Shale. Palaeontological Association, London.

(*a*)

Coll. N°	field N°	origin	Fig.	Lb (mm)	Lc (mm)	Lc/Lb	stage	shape	view	remark
ROMIP 56947	931616	WQ (-130)	12e, 18h, S11	47.13	16.12	0.34	dim 1	curved	lateral	
ROMIP 63357		WQ (+45)	see ref (*), Fig. 2a, b	55.69	18.14	0.32	dim 1 (eggs)	curved	lateral	FEMeggs
ROMIP 64287	910635	RQ	4p	52.43	14.36	0.27	dim 2	curved	lateral	
ROMIP 64282	930646	WQ (Talus)	1g; 26d, e	43.43	undet.	undet.	juvenile	straight	ventral	
ROMIP 64293	940305	WQ(-130)	14e; 24a, b	48.56	16.56	0.34	dim 1	straight	ventral	
ROMIP 56421	940677	WQ(-130)	1c; 14c, d; S3	57.66	15.31	0.26	dim 2	curved	lateral	
ROMIP 64289	940874	WQ(-130)	6i	50.24	13.40	0.27	dim 2	curved	lateral	
ROMIP 64281	941470	WQ(-130)	1e, 6g, 18i; S12b, d, e; S17e	42.96	12.05	0.28	juvenile	curved	lateral	
ROMIP 64295	950242	WQ (Talus)	2; 15a, b; 18a	42.70	undet.	undet.	juvenile	straight	ventral	
ROMIP 64296	950542	WQ(-120)	26a	40.34	undet.	undet.	juvenile	straight	ventral	
**USNM 57681a**			**1a, 19c, 26b; S5a,b**	**55.26**	**15.65**	**0.28**	dim 2	**straight**	**ventral**	**LT**
USNM 57682			S5c, S17f	66.30	19.59	0.29	dim 2	curved	lateral	
USNM 83948a			unfigured	53.48	15.84	0.29	dim 2	curved	lateral	
USNM 83948b			unfigured	62.75	18.23	0.29	dim 2	curved	lateral	
USNM 83948f			unfigured	47.90	14.78	0.31	dim 1	curved	lateral	
USNM 83948k			18f	48.82	undet.	undet.	dim undet.	straight	ventral	
USNM 83949			unfigured	46.35	15.72	0.34	dim 1	curved	lateral	
USNM 114259			S17a-d	58.06	16.32	0.28	dim 2	curved	lateral	
USNM 139214			1b, 12h, 14h	52.91	17.85	0.34	dim 1	curved	lateral	
USNM 165229			19b	13.15	3.22	0.24	juvenile	straight	dorsal	
USNM 189277			unfigured	47.35	17.10	0.36	dim 1	curved	lateral	
USNM 267750			unfigured	50.31	17.56	0.35	dim 1	curved	lateral	
USNM 268057			unfigured	65.56	24.54	0.37	dim 1	curved	lateral	
USNM 268146			unfigured	53.20	18.00	0.34	dim 1	curved	lateral	
USNM 268270			1d, 14i; 24g, h	50.53	16.47	0.32	dim 1	curved	lateral	
USNM 268338			19a	28.25	10.99	0.39	juvenile	curved	lateral	
USNM 275504			16a-f; 18b; S14a, b	53.68	15.41	0.29	dim 2	straight	ventral	
USNM 529131			1f	40.62	12.80	0.31	juvenile	curved	lateral	
USNM 529138			unfigured	49.18	13.26	0.27	dim 2	curved	lateral	
USNM 529144			unfigured	45.51	15.54	0.34	dim 1	curved	lateral	
USNM 529189			6f	58.68	19.28	0.33	dim 1	curved	lateral	
USNM 940677			unfigured	53.68	15.28	0.28	dim 2	curved	lateral	

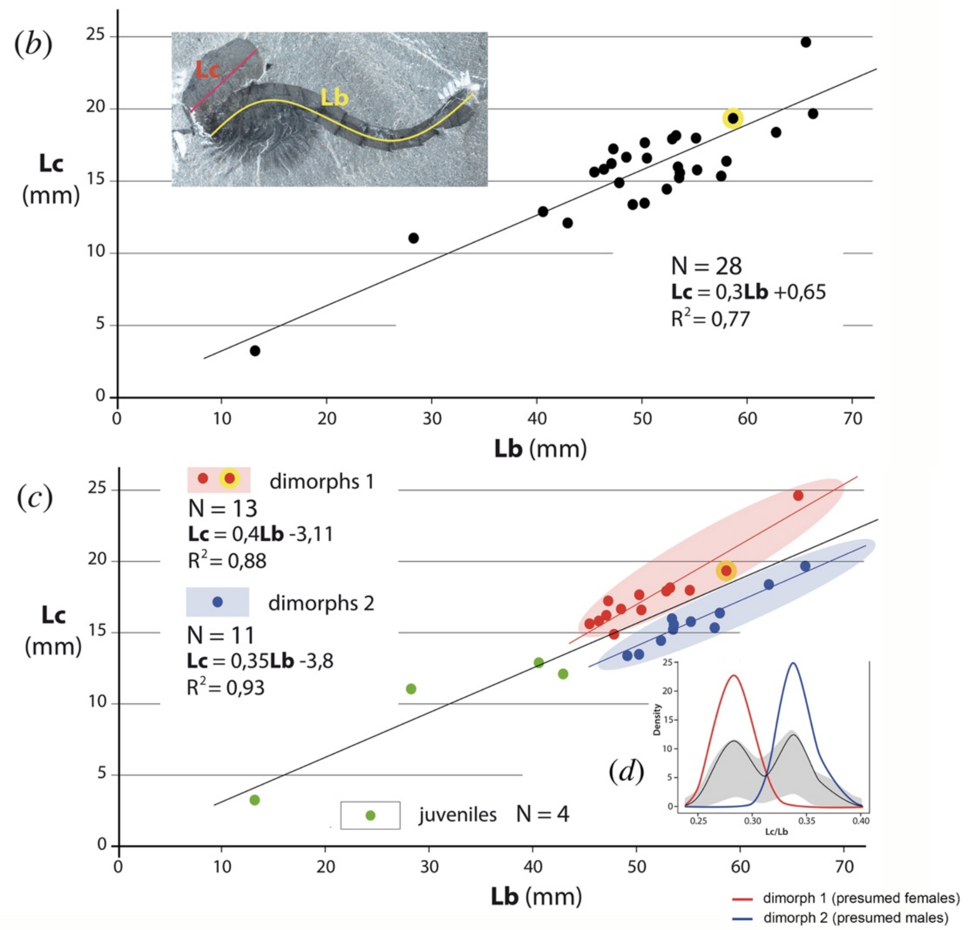

Electronic supplementary material S6. *Waptia fieldensis* Walcott, 1912 [10] from the middle Cambrian (Series 3, Stage 5) Burgess Shale, British Columbia, Canada; measurements of body and carapace length (Lb and Lc, respectively). (*a*) Data. (*b, c*) Lc to Lb ratio in 28 complete specimens. (*d*) Kernel density analysis of measured subadult and adult specimens of *Waptia fieldensis* Walcott, 1912. The black curve is the density of the whole data, showing clear bimodality, the red curve is the density of the extracted female data, and the blue curve is males. The grey area is the bootstrapped reference band calculated from female and male data, and indicates where a density estimate of the data would lie if it was normally distributed. Abbreviations are as follows: dim 1, assumed dimorphic specimens of type 1 (presumably females; red dots and line); dim 2, assumed dimorphic specimens of type 2 (presumably males; blue dots and line); FEMeggs, female carrying eggs (red dot highlighted in yellow; see Caron and Vannier (2016)); LT, lectotype; RQ, Raymond Quarry; S, Supplementary figure; WQ, Walcott Quarry.
(*) Reference: Caron JB, Vannier J. 2016 *Waptia* and the diversification of brood care in early arthropods. *Current Biology* 26, 1-6.

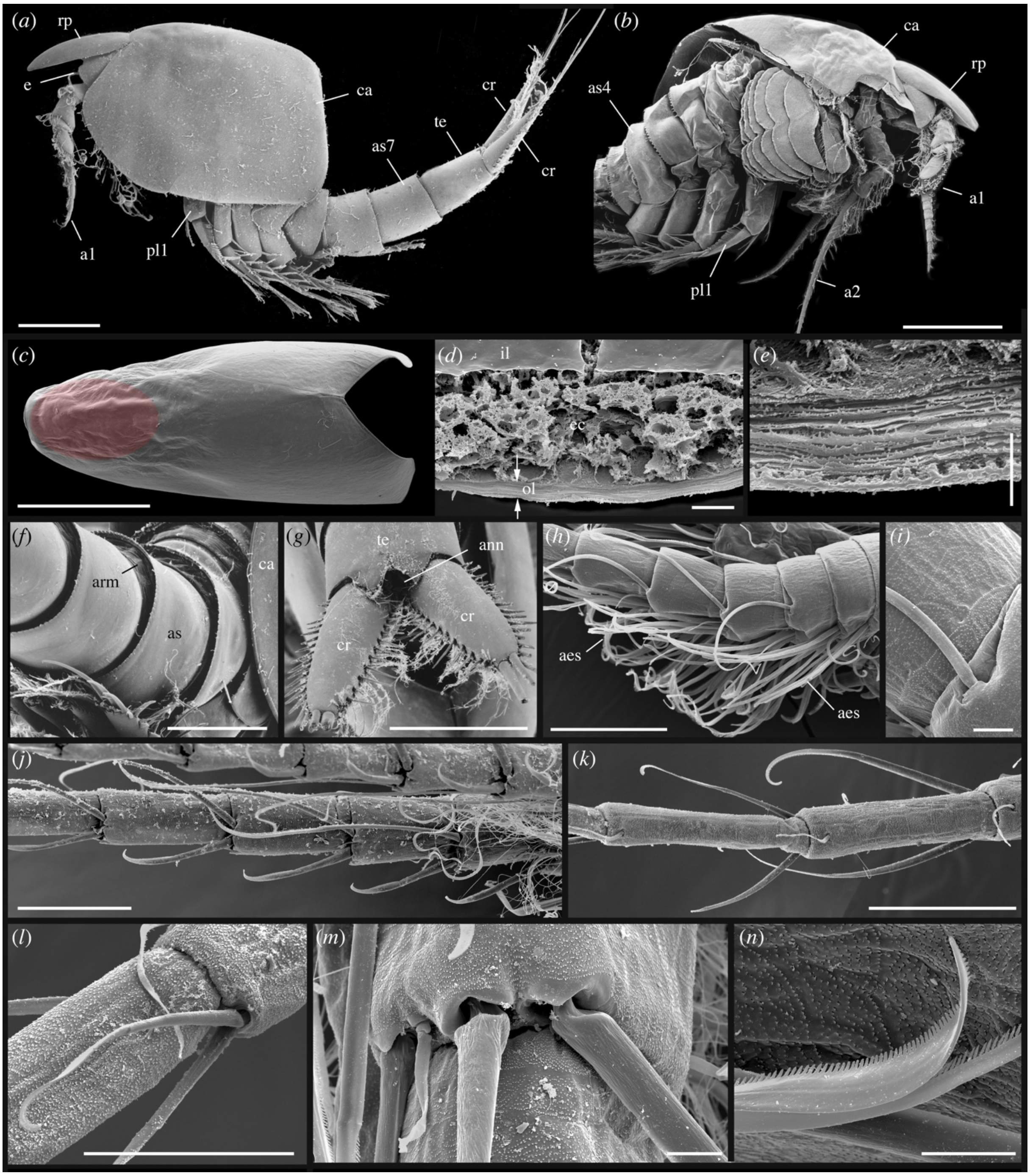

Electronic supplementary material S7. *Nebalia bipes* (Crustacea, Malacostraca, Leptostraca); general morphology. (*a*) Left lateral view. (*b*) Right lateral view, right lateral flap of carapace removed. (*c*) Dorsal view of carapace rostral plate removed. (*d*) Transverse section through carapace. (*e*) Transverse section through carapace outer lamella. (*f*) Telescopic abdominal segments in intermediate posterior view. (*g*) Ventral side of teson and caudal rami showing anus opening. (*h, i*) Antennule first antenna with bunches of aesthetascs, general view and detail. (*j, k*) Setation along second antenna. (*l, m*) Insertion of setae on second antenna. (*n*) Comb-like teeth near the tip of antennal setae. Approximate attachment area of carapace in red. Abbreviations are as follows: a1, first antenna, a2, second antenna; aes, aesthetasc; arm, arthrodial membrane; ann, anal notch; as, abdominal segment; ca, carapace; cr, caudal ramus; e, eye; ec, epidermal cells; il, carapace inner lamella; ol, carapace outer lamella; pl1, first pair of pleopod; rp, rostral plate; te, telson. Scale bars: 1 mm in *b*; 500 µm in *a, c, f, g*; 100 µm in *h, j, k*; 50 µm in *l*; 10 µm in *d, i, m, n*; 2 µm in *e*.

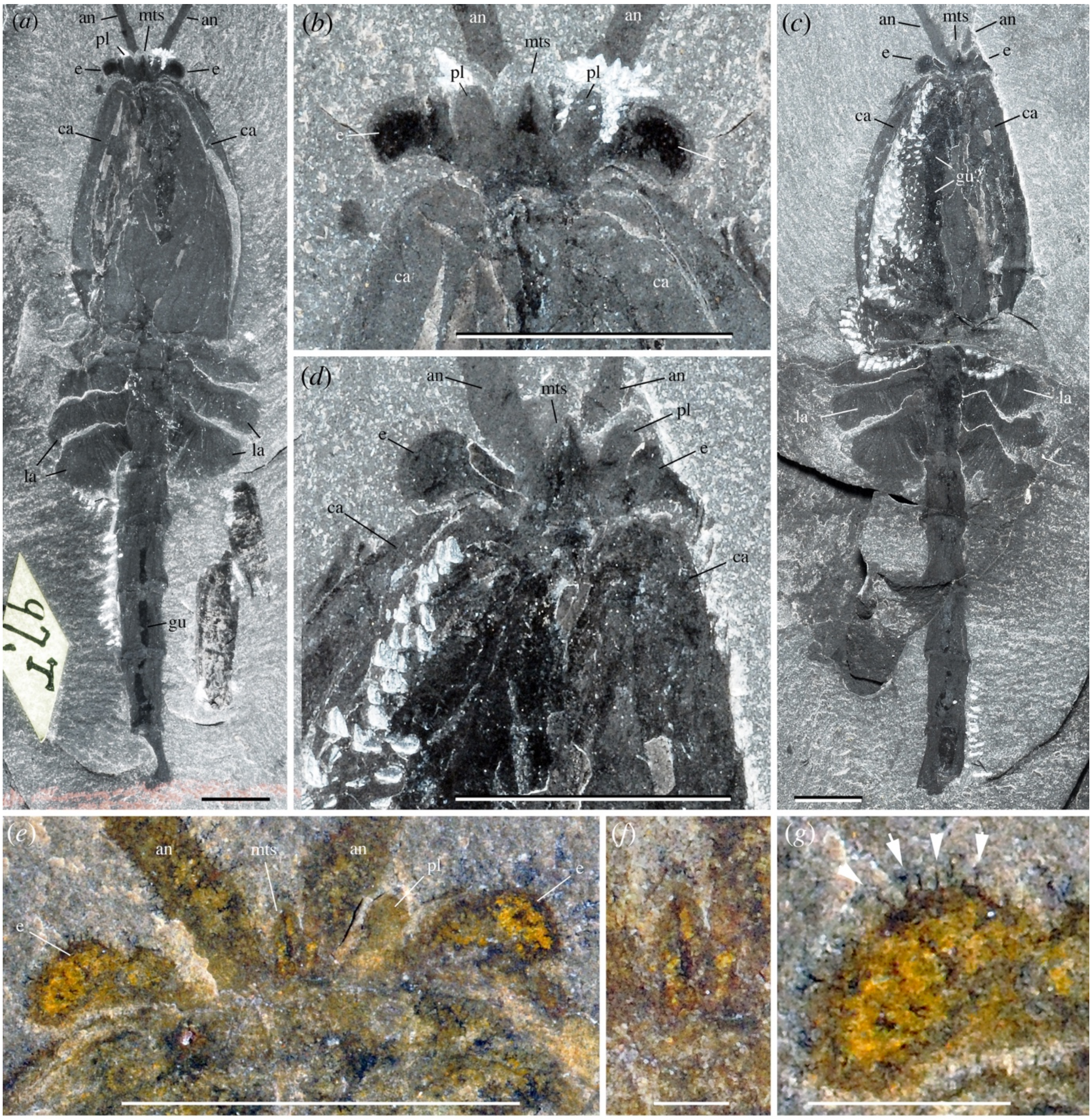

Electronic supplementary material S8. *Waptia fieldensis* Walcott, 1912 from the middle Cambrian (Series 3, Stage 5) Burgess Shale, British Columbia, Canada; ocular and inter-ocular regions. (*a, b*) USNM 83948j (part). (*c, d*) USNM 83948j (counterpart). (*e-g*) USNM 268199, general view and details of anterior median projection and eye. All images are photographs taken under cross-polarized light. Small white arrows indicate mineralized features interpreted as inter-ommatidial setae by Strausfeld (2016). Abbreviations are as follows: an, antennule; ca, carapace; e, eye; gu, gut; la, lamellate post-cephalothoracic appendage; mts, median triangular sclerite; pl, peduncular lobe. Scale bars: 5 mm in *a-e*; 1 mm in *g*; 500 µm in *f*.

Reference: Strausfeld NJ. 2016 *Waptia* revisited, intimations of behaviours. *Arthropod Structure and Development* **45**, 173-184.

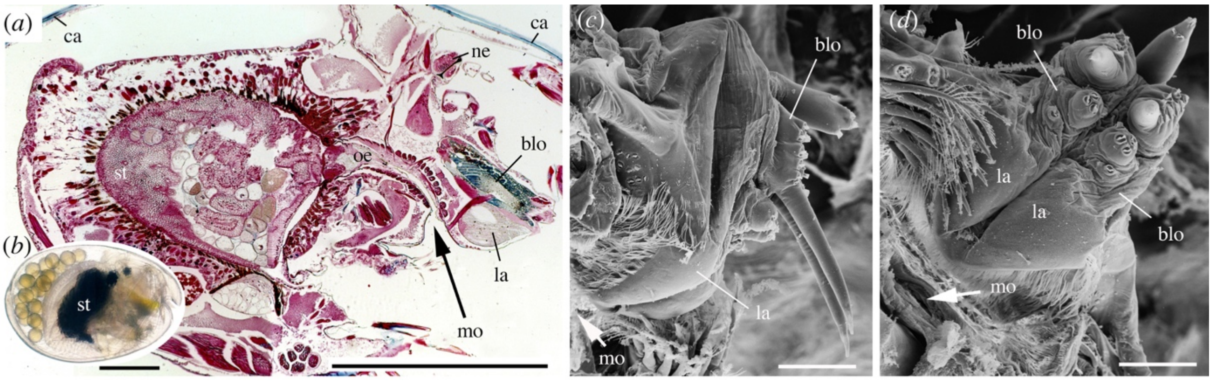

Electronic supplementary material S9. *Vargula hilgendorfii* (Crustacea, Ostracoda, Myodocopida) from Japan; details of labrum. (*a, b*) Stained sagittal paraffin section and lateral view of an adult female (translucent carapace). (*c, d*) Lateral and ventral view of the labrum (upper lip) showing its bilateral symmetry and paired structures; the labrum accommodates the bioluminescent organ. *c* and *d* are SEM images. Abbreviations are as follows: bio, bioluminescent organ; ca, carapace; la, labrum; mo, mouth; ne, nauplius eye; st, stomach. Scale bars: 1 mm in *a, b*; 100 µm in *c, d*.

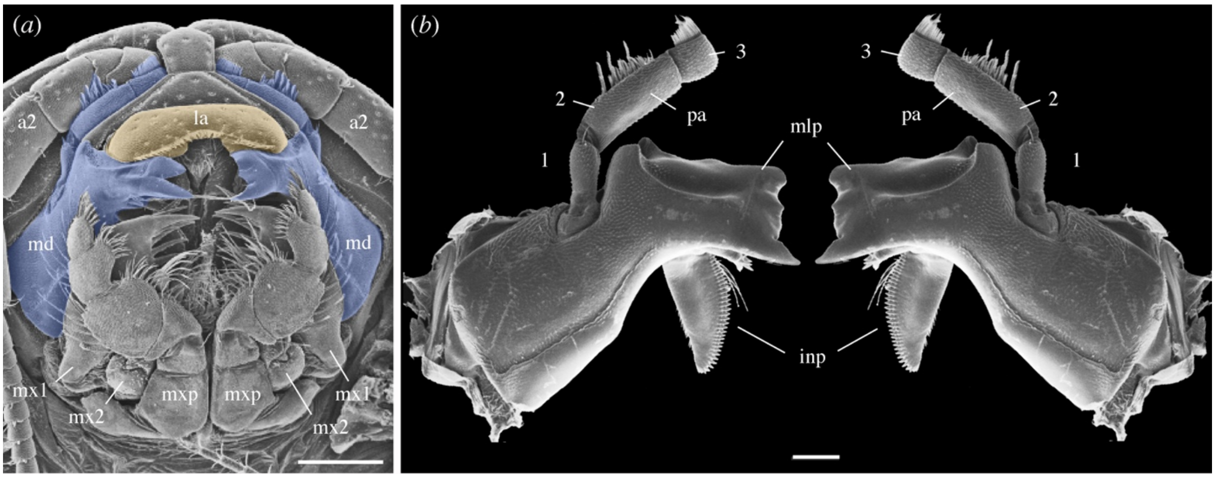

Electronic supplementary material S10. *Cirolana harfordi* (Crustacea, Isopoda), mouth parts. (*a*) General ventral view of cephalic region. (*b*) Isolated left mandible and its mirror image. All SEM images (reproduced and adapted from Thomson (2013; figs 4A and 10B) with permission to JV from Wiley Publishers; courtesy M. Thomson). Mandibles in blue, labrum in yellow. Abbreviations are as follows: a2, second antenna; inp, incisor process; la, labrum; md, mandible; mlp, molar process; mx, maxillule; mx2, maxilla; mxp, maxilliped; pa, palp; 1-3: 1st to 3rd podomere of palp. Scale bars: 300 µm in *a*; 200 µm in *b*.
Reference: Thomson M. 2013 Mouthparts and their setae of the intertidal isopod *Cirolana harfordi*. *Journal of Microscopy* **252**, 111-121.

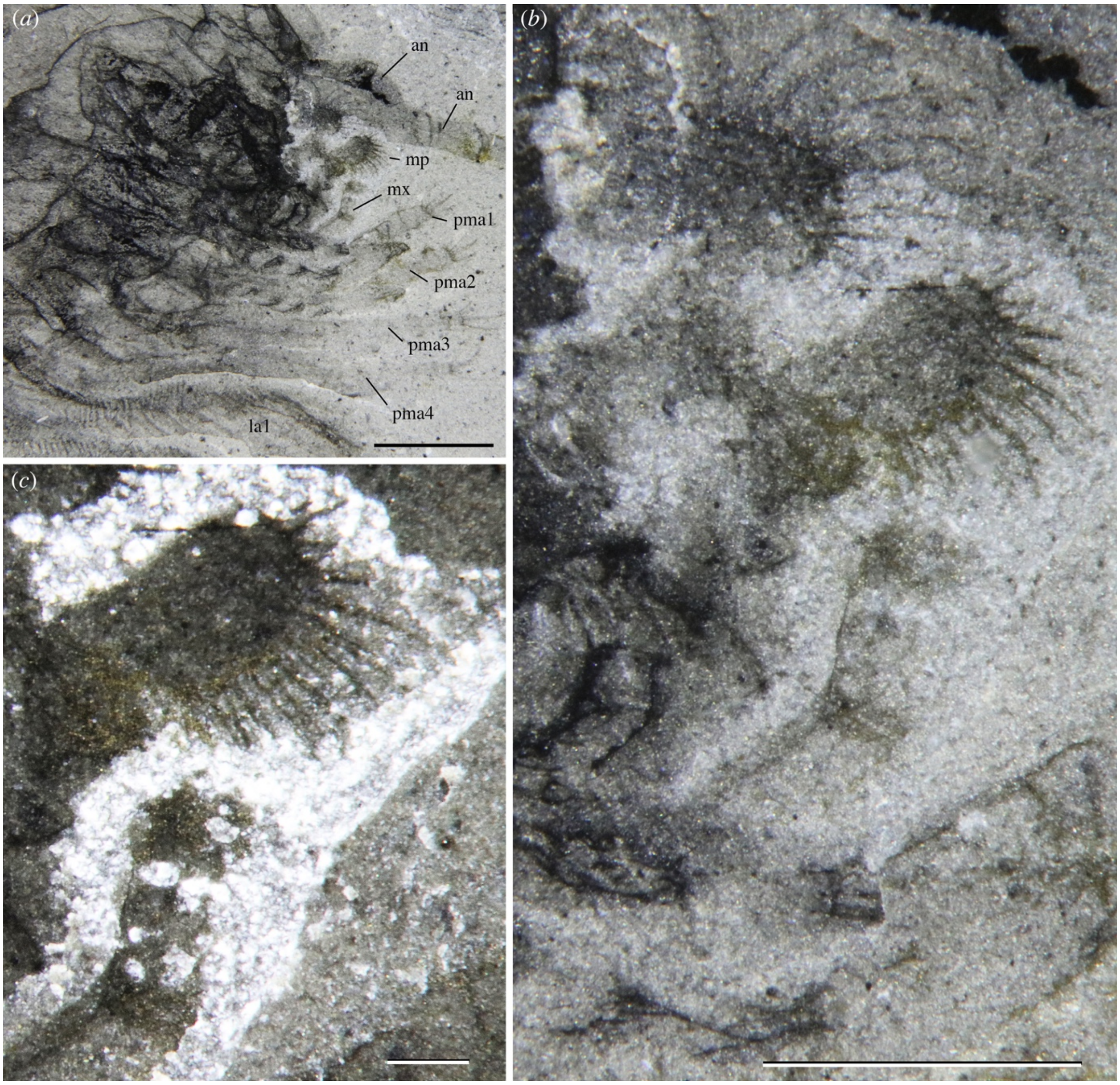

Electronic supplementary material S11. *Waptia fieldensis* Walcott, 1912 from the middle Cambrian (Series 3, Stage 5) Burgess Shale, British Columbia, Canada; ROMIP 56947, mandibular palp and maxillule. (*a*) General view. (*b, c*) Details showing right and left mandibular palps fringed with numerous setae and part of the maxillule. All images are photographs taken under cross-polarized light. Abbreviations are as follows: an, antenna; la1, 1st lamellate post-cephalothoracic appendage; mp, mandibular palp; mx, maxillule; pma1-4, 1st to 4th post-maxillular cephalothoracic appendages. Scale bars: 1 mm in *a*; 500 µm in *b*; 100 µm in *c*.

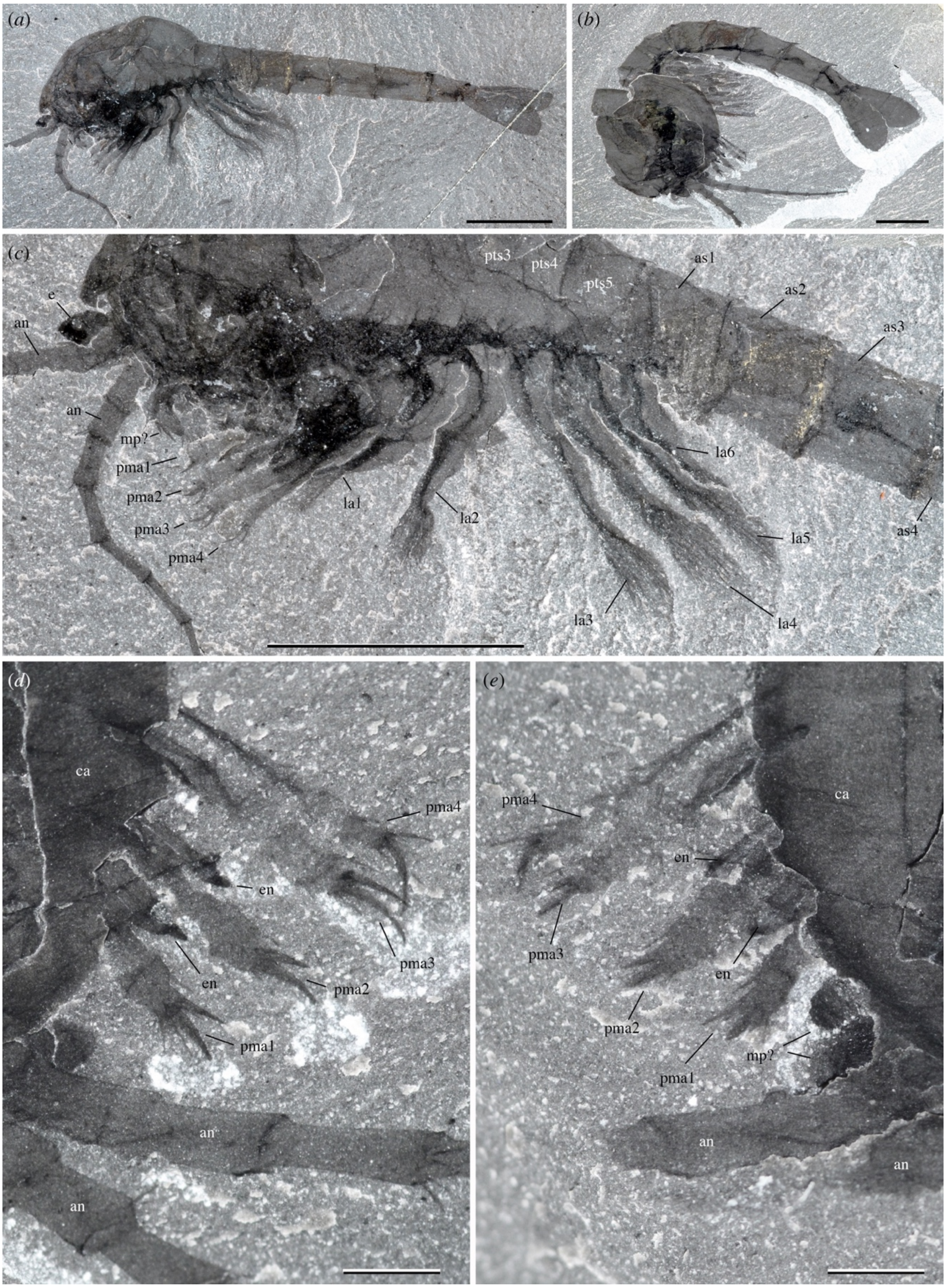

Electronic supplementary material S12. *Waptia fieldensis* Walcott, 1912 from the middle Cambrian (Series 3, Stage 5) Burgess Shale, British Columbia, Canada; post-maxillular cephalothoracic appendages. (*a, c*) USNM 139214, general view and details in lateral view. (*b, d, e*) ROMIP 64281, general view and details of appendages (part and counter part). All images are photographs taken under cross-polarized light. Abbreviations are as follows: an, antennule; as1-6, 1st to 6th abdominal segments; ca, carapace; e, eye; en, endite; la1-6, 1st to 6th lamellate post-cephalothoracic appendages; mp, mandibular palp; pma1-4, 1st to 4th post-maxillular cephalothoracic appendages; pts1-5, 1st to 5th thoracic segments. Scale bars: 1 cm in *a-c*; 5 mm in *b*; 1 mm in *d, e*.

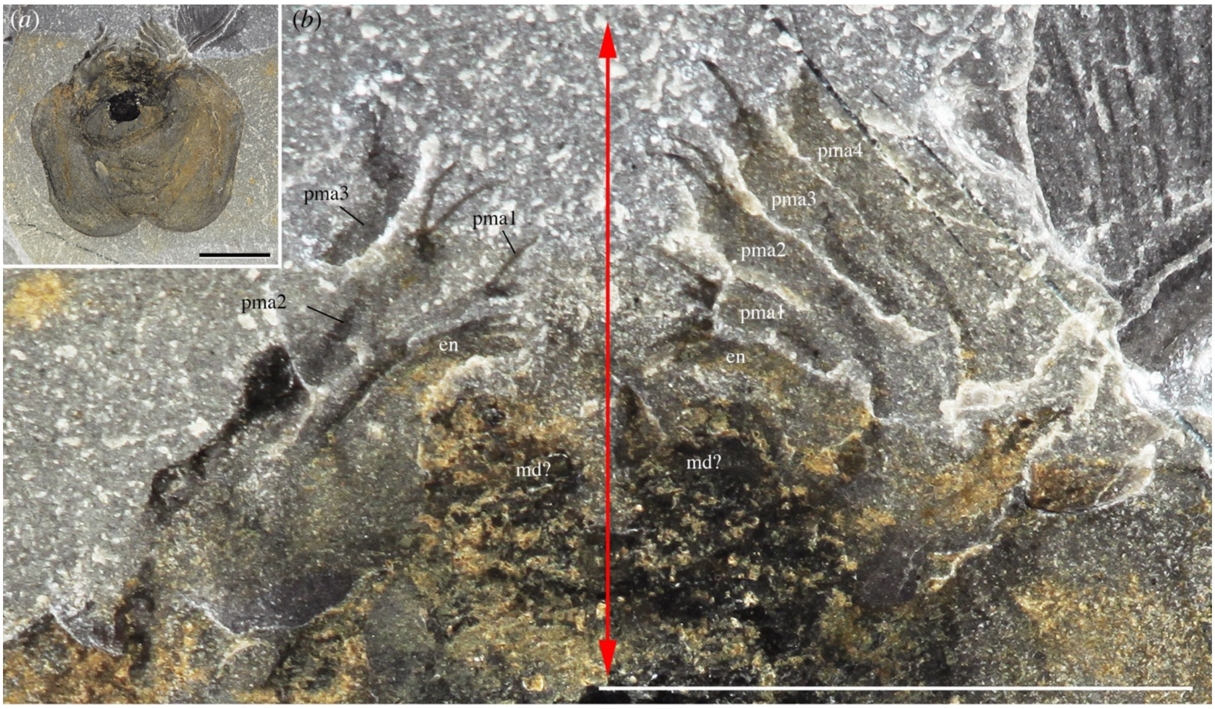

Electronic supplementary material S13. *Waptia fieldensis* Walcott, 1912 from the middle Cambrian (Series 3, Stage 5) Burgess Shale, British Columbia, Canada; ROMIP 64385, post-maxillular appendages in intermediate frontal view. (*a, b*) General view of specimen and details of appendages converging towards the sagittal plane (double red arrow). Both images are photographs taken under cross-polarized light. Abbreviations are as follows: en, endite; md, mandible; pma1-4, 1st to 4th post-maxillular cephalothoracic appendages. Scale bars: 5 mm

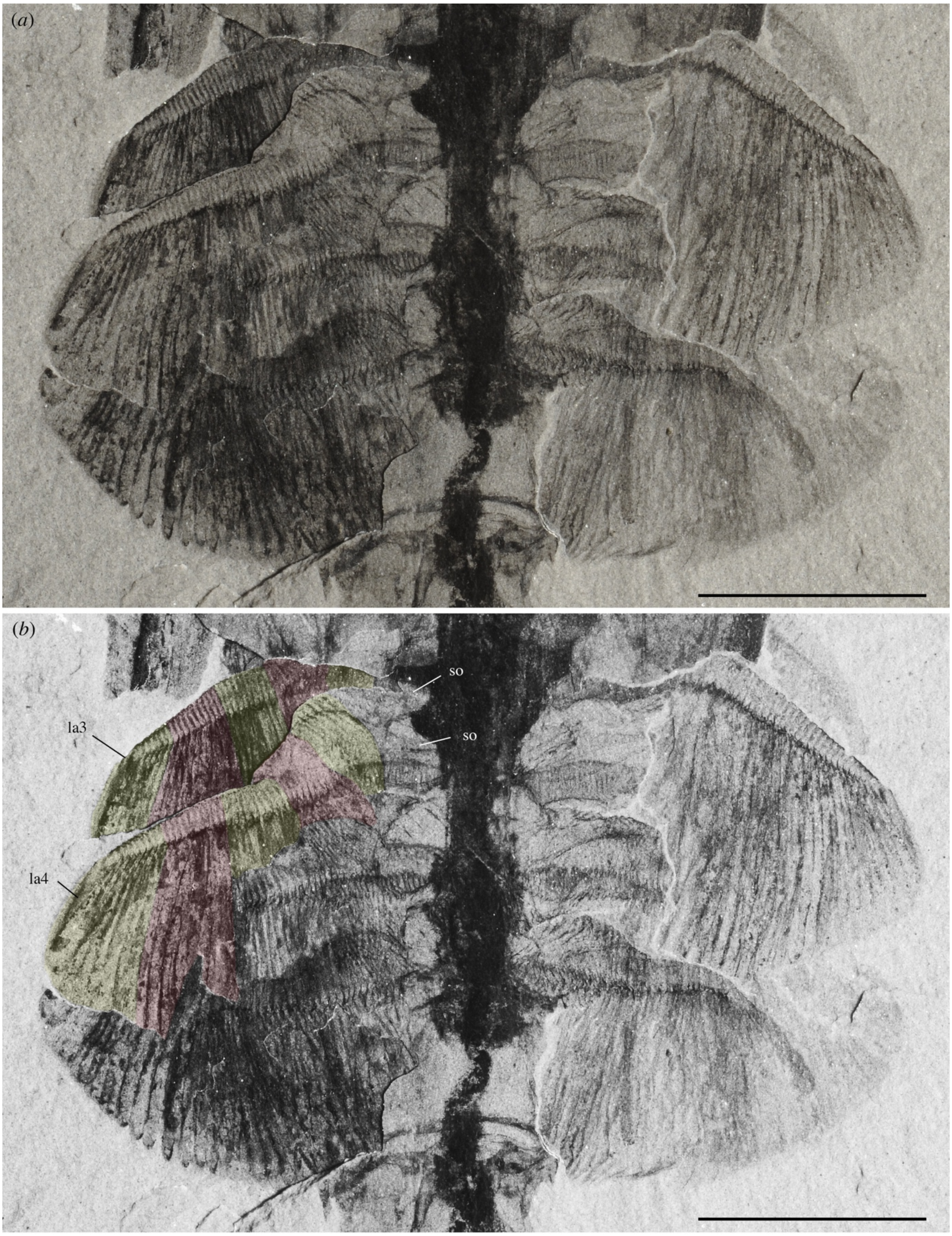

Electronic supplementary material S14. *Waptia fieldensis* Walcott, 1912 from the middle Cambrian (Series 3, Stage 5) Burgess Shale, British Columbia, Canada (*a, b*) USNM 275504, details of lamellate appendages; each coloured area corresponds to 10 annulations and lamellae. Both images are photographs taken under cross-polarized light. Abbreviations are as follows: la3-4, 3rd to 4th lamellate post-cephalothoracic appendage; so, socket. Scales bars: 5 mm.

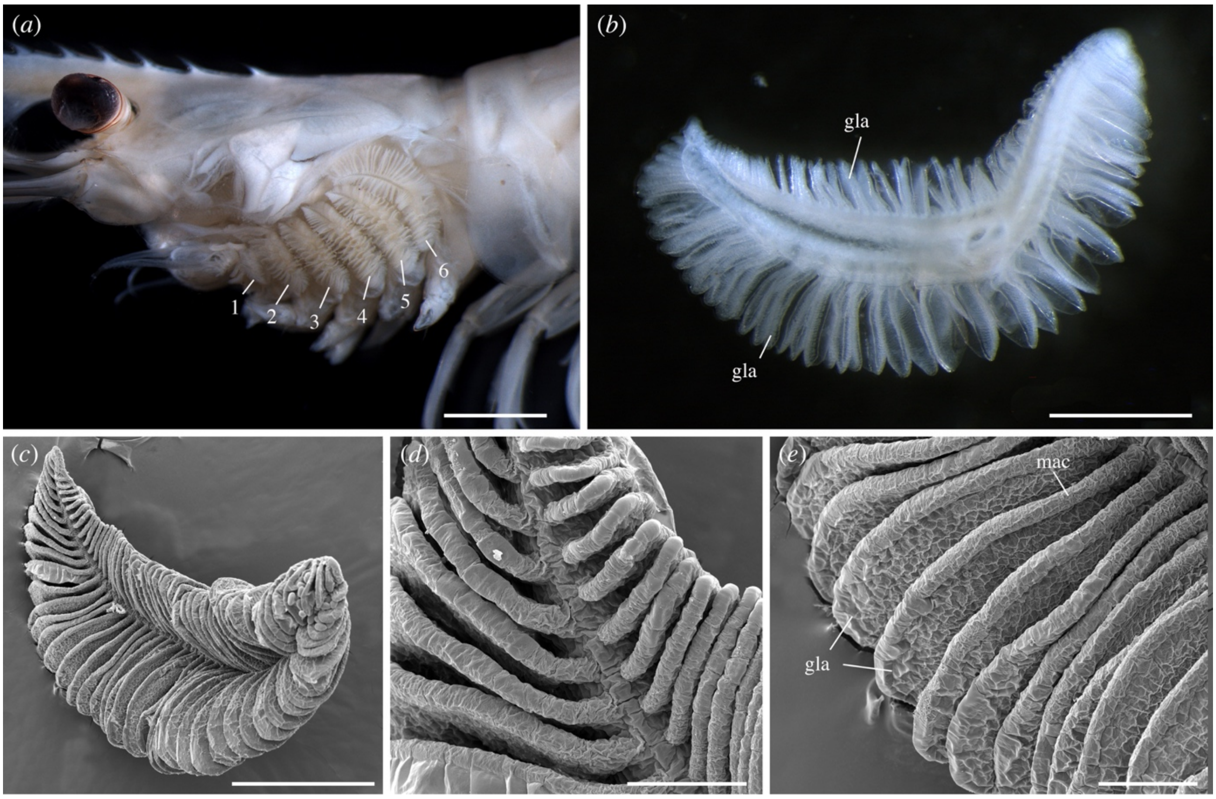

Electronic supplementary material S15. *Crangon crangon* (Crustacea, Decapoda, Caridea), phyllobranchiate gills. (*a*) Left side of carapace removed showing 6 gills attached to thoracic appendages. (*b*) Isolated gill. (*c-e*) General view of gill, details of axial structure and lamellae. Abbreviations are as follows: gla, gill lamella; mac, marginal canal. 1-6, 1st to 6th phyllobranchiate gills. *a, b* are light photographs; c-e are SEM images. Scale bars: 2 mm in *a*; 500 µm in *b, c*; 100 µm in *d, e*.

Coll. N°	Field N°	Fig.	as1	as2	as 3	as4	as5	preservation
ROMIP 56421	940677	1c; 14c, d; S3	0.83	1.00	1.16	1.56	1.86	LATERAL straight
ROMIP 56432	931318	8a-k, 9h, 11h, 12f; 14a,b	0.83	0.94	1.04	1.27	1.78	LATERAL slightly curved
ROMIP 64287	910635	4p, S18a-d	0.83	0.96	1.1	1.27	1.74	LATERAL straight
ROMIP 64289	940874	6i	0.93	1.23	1.32	1.59	1.87	LATERAL straight
ROMIP 64281	941470	1e, 6g, 18i; S12b,d,e, S17e	0.89	1.10	1.10	1.16	1.54	LATERAL slightly curved
USNM 57682		6h, 18g, S5c, S17f	0.89	0.91	1.12	1.28	1.65	LATERAL slightly curved
USNM 83948a		unfigured	0.92	1.13	1.31	1.57	1.88	LATERAL slightly curved
USNM 114259		S17a-d	0.97	1.11	1.23	1.60	1.84	LATERAL straight
USNM 139214		1b, 12h, 14h, 15d; S12a, c	0.94	1.03	1.14	1.35	1.83	LATERAL slightly curved
USNM 268146		unfigured	0.87	1.00	1.10	1.36	1.72	LATERAL straight
USNM 529131		1f	0.85	0.95	1.09	1.25	1.56	LATERAL slightly curved
USNM 529138		unfigured	0.89	1.12	1.25	1.43	2.00	LATERAL straight
			**0.88**	**1.04**	**1.16**	**1.39**	**1.77**	
ROMIP 64282	930646	1g; 26d, e	0.79	1.03	1.30	1.40	1.95	VENTRAL (?) straight
ROMIP 64285	941339	3e, 10a-d	0.78	0.97	1.00	1.22	1.65	VENTRAL straight
ROMIP 64293	940305	14e; 24a, b	1.05	1.15	1.14	1.42	1.74	DORSAL straight
ROMIP 64295	950242	2a-o; 15a, b; 18a	1.04	1.26	1.35	1.55	1.97	VENTRAL straight
**USNM 57681a (L)**		**19c, S5a,b**	**1.06**	**1.33**	**1.33**	**1.69**	**2.04**	**VENTRAL straight**
USNM 83948k		18f	1.00	1.17	1.11	1.35	1.77	VENTRAL straight
			**0.95**	**1.15**	**1.2**	**1.49**	**1.85**	
**JUVENILES**								
USNM 165229		19b	0.83	0.83	0.90	1.05	1.30	DORSAL straight
USNM 268338		19a	0.78	0.84	0.92	1.09	1.33	LATERAL slightly curved

maximum value
minimum value
mean value

L= Lectotype

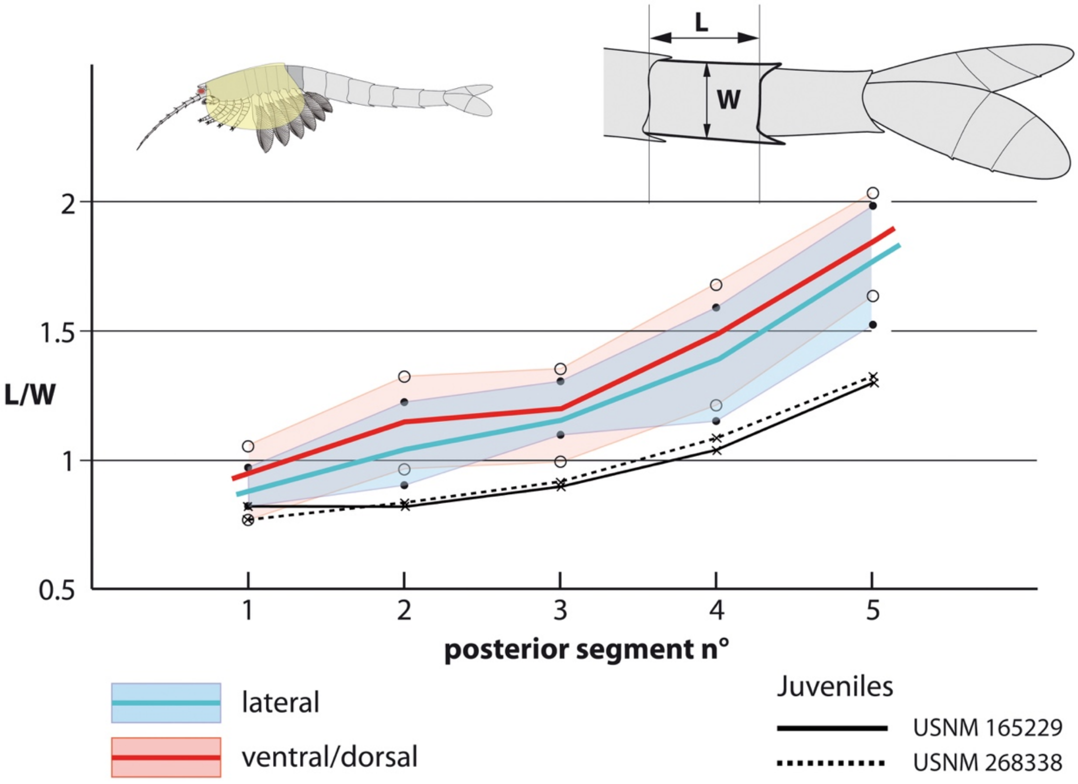

Electronic supplementary material S16. *Waptia fieldensis* Walcott, 1912 from the middle Cambrian (Series 3, Stage 5) Burgess Shale, British Columbia, Canada; measurements of the abdominal segments. Table and graph showing the variation of the length to width ratio (L/W) in the 1st to 5th abdominal segments. Abbreviations are as follows: as1-5, 1st to 5th abdominal segment; L, Length of abdominal segment; S, Supplementary figure; W, Width of abdominal segment.

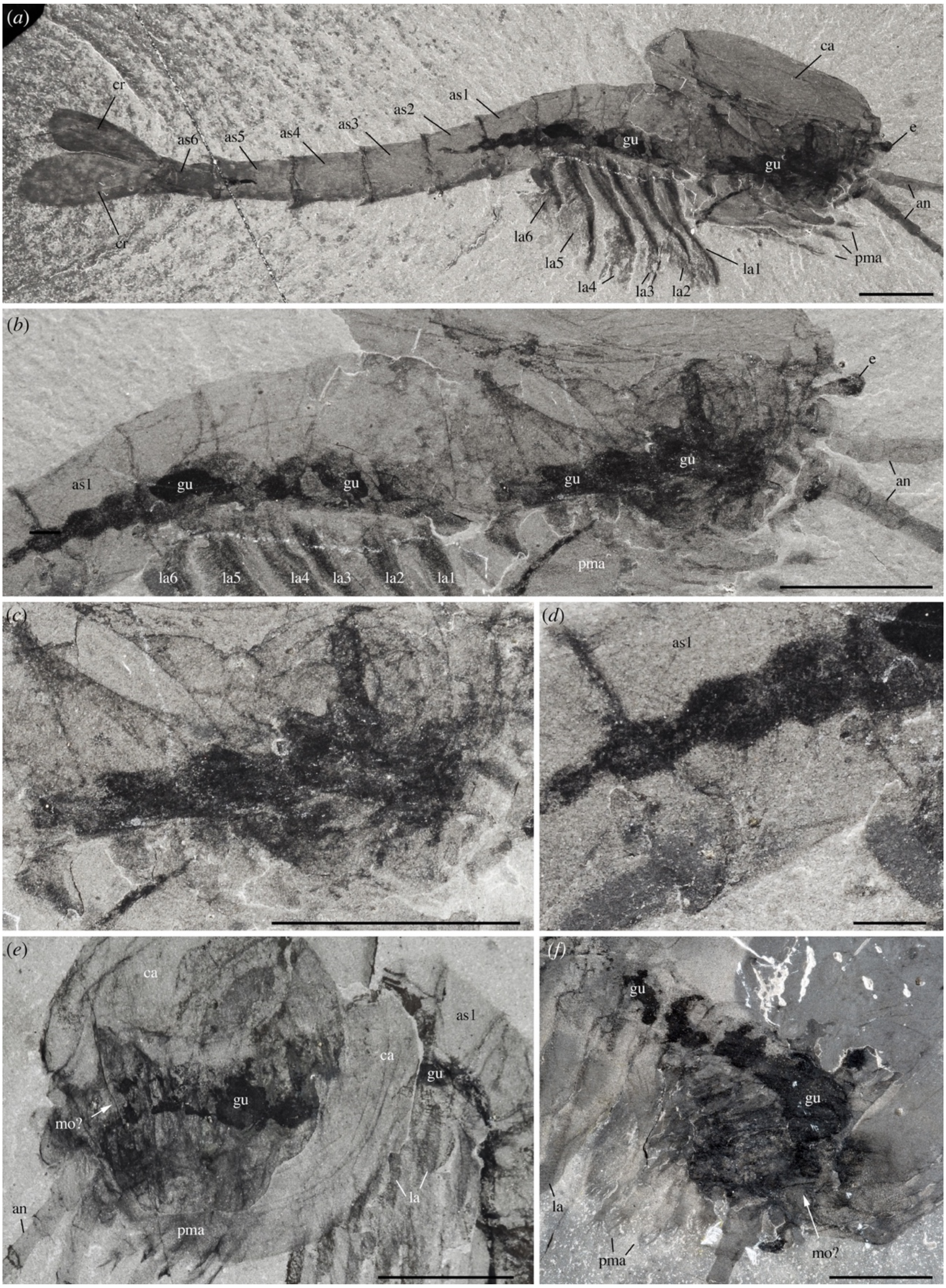

Electronic supplementary material S17. *Waptia fieldensis* Walcott, 1912 from the middle Cambrian (Series 3, Stage 5) Burgess Shale, British Columbia, Canada; digestive system. (*a-d*). USNM 114259, general view and details showing anterior and middle part of the gut. All images are light photographs taken under cross-polarized light. (*e*) ROMIP 64281, anteriormost part of the gut; (*f*) USNM 57682, anteriormost part of the gut (overall view see Electronic supplementary material S5c**)**. Abbreviations are as follows: an, antennule; as1-6, 1st to 6th abdominal segments; ca, carapace; cr, caudal ramus; e, eye; gu, gut; la1-6, 1st to 6th pair of lamellate post-cephalothoracic appendages; pma, post-maxillular cephalothoracic appendage. Scale bars: 5 mm in *a-c, e, f*; 1 mm in *d*.

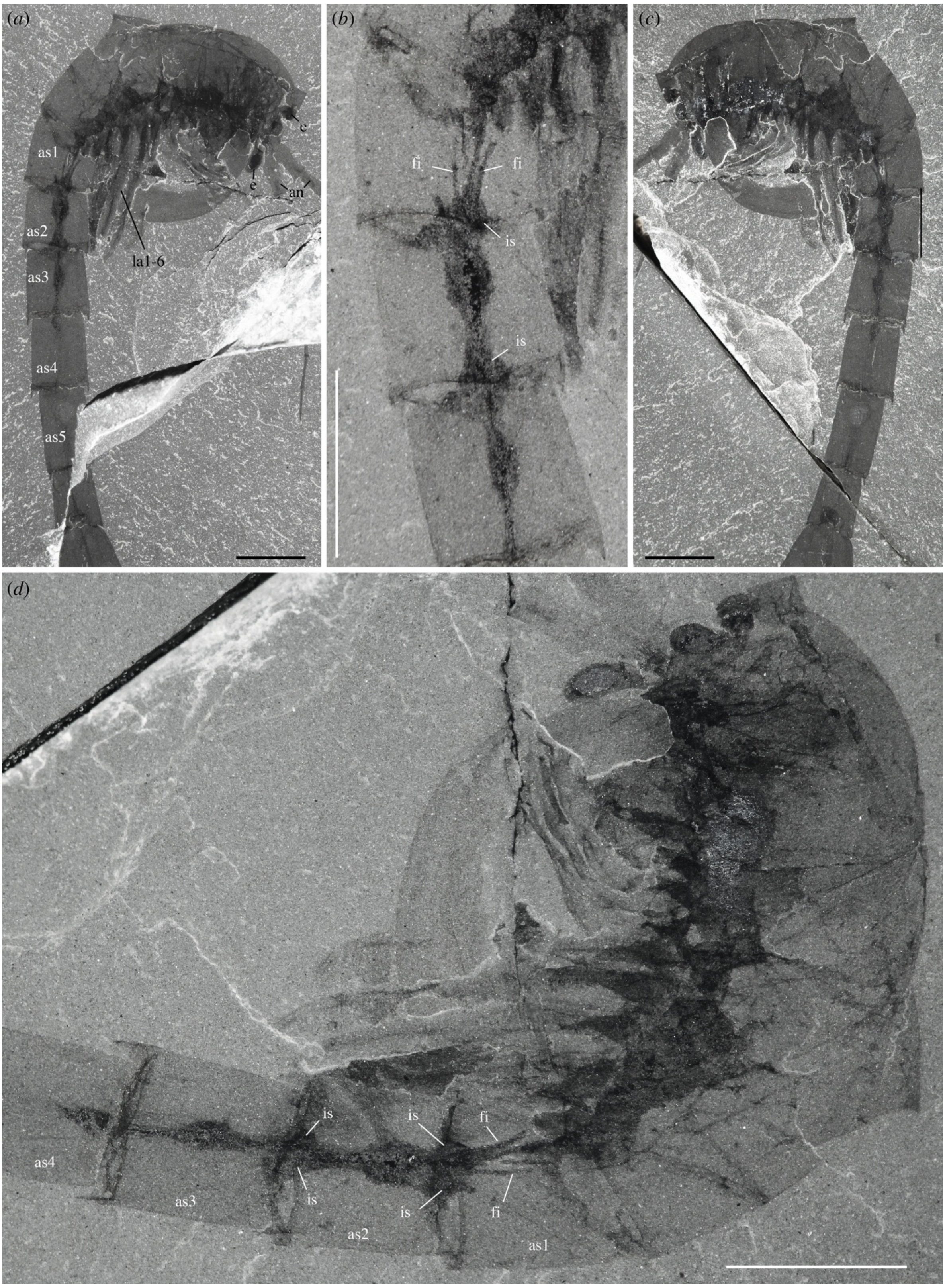

Electronic supplementary material S18. *Waptia fieldensis* Walcott, 1912 from the middle Cambrian (Series 3, Stage 5) Burgess Shale, British Columbia, Canada; digestive system, ROMIP 64287, part and counterpart. (*a, c*) General views. (b, d) Details showing intestinal swellings and filament-like structures. All images are light photographs taken under cross-polarized light. Abbreviations are as follows: as1-5, 1st to 5th abdominal segments; fi, ramifying filaments; is, intestinal swelling; la1-6, 1st to 6th pair of lamellate post-cephalothoracic appendages. Scale bars: 5 mm.

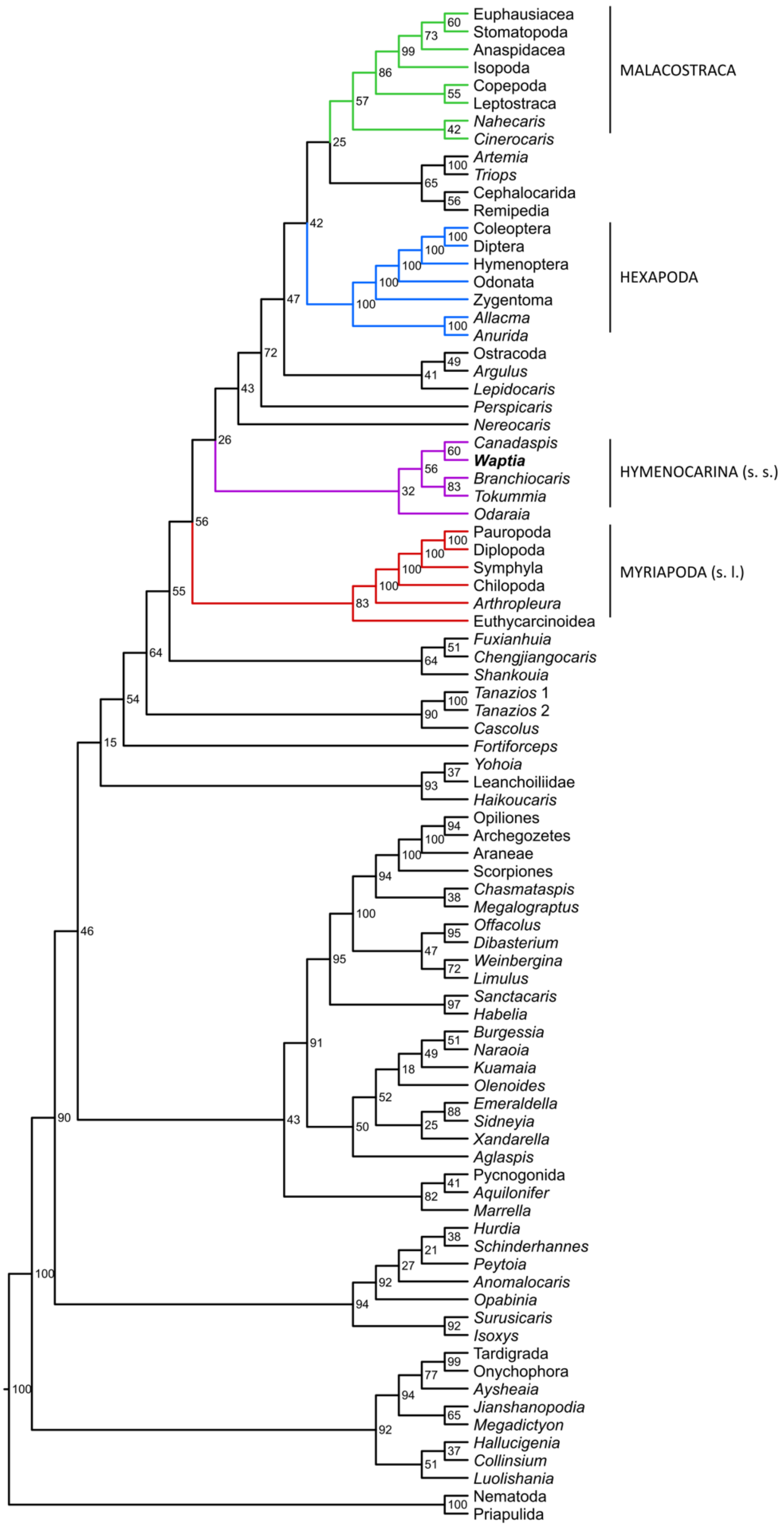

Electronic supplementary material S19. Maximum clade credibility trees of time-calibrated Bayesian analyses of panarthropod relationships (Mkv+Γ model, 219 characters). Consensus topology resulting from a dataset of 85 adult taxa. Numbers on the right of nodes are posterior probabilities.

Electronic supplementary material S20. List of characters used in the present cladistic analysis.

CHARACTER LIST

The following list is taken from Aria and Caron (2017a) and includes the changes made for the present study as well as those from Aria and Caron (2017b). Character headings refer to the original publication by [ACX], where X is the corresponding number for that character in Aria and Caron (2017). Remarks are not carried over here if no change was made. Characters either new since Aria and Caron (2017), or which have gone through substantial change in overall coding or definition are marked with an asterisk. Some other small corrections were applied to the original matrix but are not reported here in detail.

GENERAL CHARACTERS

[1] Limbs [AC1]
  0. Absent
  1. Present

[2] External cuticular segmentation [AC2]
  0. Absent
  1. Present

[3] Type of segmentation [AC3]
  0. Sclerotized
  1. Arthrodized (=tergal)

[4] Calcified cuticle [AC4]
  0. Absent
  1. Present

[5] Main calcification type [AC5]
  0. Calcium carbonate
  1. Calcium phosphate

[6] Holometaboly [AC6]
  0. Absent
  1. Present

LOBOPODIAN CHARACTERS

[7] External anteriorization restricted to a single pair of frontalmost appendages [AC7]
  0. Absent
  1. Present

[8] Lobopodous limbs [AC8]
  0. Absent
  1. Present

[9] Type of main lobopodous trunk limb [AC9]
  0. Short, conical, subequal or shorter than trunk width
  1. Elongated, slender, longer than trunk width

[10] Flap-like lateral limbs [AC10]
  0. Absent

1. Present

[11] Nodes/tubercles/dermal papillae [AC11]
0. Absent
1. Present

[12] Differentiation at limb insertion [AC12]
0. Absent
1. Present

[13] Dorso-lateral sclerites above limb insertion [AC13]
0. Absent
1. Present

[14] Median spine above limb insertions [AC14]
0. Absent
1. Present

[15] Lobopod tip (main trunk limb) [AC15]
0. Double claw
1. Juxtaposed series of claws
2. Pad

[16] Posterior-most single claws [AC16]
0. Absent
1. Present

[17] Posterior claws pointing anteriad [AC17]
0. Absent
1. Present

VISUAL ORGANS

[18] Ocelli as primary ocular units [AC18]
0. Absent
1. Present

[19] Median eyes [AC19]
0. Absent
1. Present

[20] Number of median eyes [AC20]
0. 2
1. 3
2. 4

[21] Rhabdomeric lateral eye [AC21]
0. Absent
1. Present

[22] Type of lateral eyes [AC22]
0. Simple lens with cup-shaped retina
1. Faceted (compound)
2. Stemmata

[23] Type of corneagenous cells [AC23]

0. Many
1. Two

[24] Tetraconate condition [AC24]
0. Absent
1. Present

[25] Number of nested optic neuropils [AC25]
0. 1
1. 2
2. 3

[26] Multi-layered rhabdomeres [AC26]
0. Absent
1. Present

[27] Eyes embedded within tergal shield [AC27]
0. Absent
1. Present

[28] Opthalmic ridges [AC28]
0. Absent
1. Present

[29] Lateral eyes pedunculate [AC29]
0. Absent
1. Present

[30] Pedunculate eyes large and ovate, part of a prominent ocular segment projecting anteriad [AC30]
0. Absent
1. Present

HEAD AND CEPHALIC CHARACTERS

[31] Somital head (as tagma I) defined by series of appendages and/or external segmentation [AC31]
0. Absent (only anteriormost defines head)
1. Present

[32] Somites defining anteriormost tagma* [AC31]
0. 5
1. 6
2. 7
3. 8

Remark: Habeliidans and relevant synziphosurines are coded for seven-segmented heads following Aria and Caron (in press).

[33] Tergite of the ocular (protocerebral) somite [AC127]
0. Absent
1. Present

[34] Tergite of the ocular (protocerebral) somite, type [AC128]
0. Rounded
1. Sub-triangular

[35] Tergal sclerotization of the post-ocular somite [AC33]

0. Absent
1. Present

[36] Tergal sclerotization type [AC34]
0. Tergites with posterior expansion, some cephalic tergites can be freely articulating (carapace)
1. Tergites with limited expansion, cephalic tergites fused (shield)

[37] Carapacal valves [AC35]
0. Bivalved
1. Fused

[38] Bivalved carapace type*
0. Type I: Sub-straight cross-section, covering body similar to head shield
1. Type II: Convex cross-section, enveloping body laterally

[39] Type II bivalved configuration [AC36]*
0. Unfused along most of dorsal margin
1. Fused along most of dorsal margin

Remark: Among hymenocarines, we consider that *Odaraia* and *Waptia* present an extensive fusion of the valves with effacement of the median fold.

[40] Covering of the type II bivalved carapace (when body fully extended antero-posteriorly) [AC37]
0. At least two thirds of body length
1. Cephalothorax

[41] Articulation of posterior margin of shield with first trunk segment [AC39]
0. Tergal overlap
1. Closure

[42] Segmental impression in shield [AC40]
0. Absent
1. Present

[43] Occipital lobe [AC41]
0. Absent
1. Present

[44] Pair of occipital carinae [AC42]
0. Absent
1. Present

[45] Anterior reduction of segments and/or appendages [AC43]
0. Absent
1. Present

[46] Compaction of the cephalic unit [AC44]
0. Absent
1. Present

[47] Doublure [AC45]
0. Absent
1. Present

[48] Cephalic kinesis [AC46]
0. Absent

1. Present

BRAIN CHARACTERS

[49] Ganglia of post-oral appendages fused into single nerve mass [AC47]
0. Absent
1. Present

[50] Contiguity of the first two post-protocerebral ganglia [AC48]
0. Absent
1. Present

[51] Fan-shaped body in brain [AC49]
0. Absent
1. Present

[52] Position of midline neuropil [AC50]
0. Superficial to protocerebrum
1. Embedded within protocerebral matrix

[53] Olfactory lobes linked to a lateral component of protocerebrum by olfactory globular tract [AC51]
0. Absent
1. Present

[54] Deutocerebral olfactory lobe with glomeruli [AC52]
0. Absent
1. Present

[55] Lateral eyes pedunculated [AC53]
0. Absent
1. Present

STERNITES (CEPHALON)

[56] Sternites [AC54]
0. Absent
1. Present

[57] Endosternum [AC55]
0. Absent
1. Present

[58] Labrum*
0. Absent
1. Present

Remark: Characters describing the hypostome-labrum complex have been reorganized and expanded compared to Aria and Caron (2017). Here, we code the labrum as a common feature shared by all euarthropods, which arguably originates from the protocerebral somite (Scholtz and Edgecombe 2006). Classically, in mandibulates, the "labrum" covers the mouth and such pre-oral structure generally takes two forms: the typical fleshy protrusion of oligostracans, anostracans and other crustaceans, and the sclerotic plates encountered in myriapods, malacostracans and hexapods—which are commonly designed as either "epistome-labrum" or "clypeo-labrum" depending on the group of interest. Pre-labral sclerites in mandibulates are often distinct from the labrum per se and are usually closely associated with the insertion of the antennules. For this reason, the pre-labral sclerite is sometimes referred to as the "hypostome," a

common term from the pre-oral sclerite used among extinct euarthropods, especially trilobites and their allies. Such a terminology has been used for instance for Cephalocarida (Olesen *et al.* 2011) but arguably applies to all other crustaceans, including copepods (Schram 1986), leptostracans (Olesen and Walossek 2000) and stomatopods (Haug *et al.* 2012). Herein (chars. 62-64), we therefore harmonize the terms "epistome," "clypeus" and "hypostome" under the same concept of "hypostome."

[59] Labrum expression and location*
0. Expressed anteriormost or not distinct
1. Expressed as ventral pre-oral structure

Remark: This character fundamentally distinguishes the anteriormost chelicerate "epistome-labrum" from ventral structures characteristic of Mandibulata. Hymenocarines are considered to belong to the first group.

[60] Type of ventral labrum* [AC58]
0. Single fleshy protrusion
1. Plate with underlying soft tissues

[61] Fusion of pre-oral structures*
0. Absent
1. Present

[62] Hypostome [AC56]*
0. Absent
1. Present

Remark: The hypostome is here defined on the basis of the artiopodan hypostome, that is, as a pre-oral sclerotic structure associated with the insertion of the anteriormost appendages.

[63] Hypostome type [AC57]*
0. Conterminant
1. Natant

[64] Hypostome accommodating antennules and extensively covering the mouth [AC59]
0. Absent
1. Present

Remark: This is a character mostly discriminating the typical artiopodan hypostome.

[65] Labium [AC60]
0. Absent
1. Present

[66] Post-hypostomal sternites externally developed within segments 2–4 [AC61]
0. Absent
1. Present

[67] Fusion of post-hypostomal sternites externally developed within segments 2–4 [AC62]
0. Absent
1. Present

[68] Metastoma [AC63]*
0. Absent
1. Present

Remark: The metastoma is the modified sternite of the first opisthosomal segment in euchelicerates—typically, eurypterids (Dunlop 1997). We also code this character for scorpions (sternum) and harvestmen (aculi genitales) (Dunlop and Lamsdell 2016).

[69] Coxosternite [AC64]
0. Absent
1. Present

[70] Both larval and imaginal head has tendention to form a hypostomal bridge [AC65]
0. Absent
1. Present

FRONTALMOST APPENDAGES

[71] Arthrodization of first axial appendage [AC66]
0. Absent
1. Present

[72] Ocular (=peduncular) lobes [AC67]
0. Absent
1. Present

[73] Bipartite frontalmost ventral protrusion [AC68]*
0. Absent
1. Present

Remark: This character directly relates to chars. 59-60 insofar as the frontalmost bipartite features observed in hymenocarines are homologized here with the labral lobes of chelicerates. We also code the state present for a number of "Orsten" larvae, given that the "median eyes" identified in these fossils and their subtriangular median sockets are virtually indistinguishable from the anteriormost bipartite lobes seen in protocaridids (Aria and Caron 2017).

[74] Branching frontalmost appendage [AC69]
0. Absent
1. Present

[75] Rami of branching frontalmost appendage originating from different podomeres [AC70]
0. Absent
1. Present

[76] Frontalmost appendage with flagellate extensions [AC71]
0. Absent
1. Present

[77] Frontalmost appendage a chelicera, i.e. chelate or subchelate with only two opposing faces [AC72]
0. Absent
1. Present

[78] Orientation of first axial appendage [AC73]
0. Ventro-frontal
1. Dorsal

[79] Segmentation of frontalmost arthrodized appendage [AC74]
0. Multi-segmented
1. Reduced

[80] Arthrodized frontalmost appendage, multi-segmented type [AC75]
0. Robust, thick branch
1. Long antennular

[81] Inner (ventral) spinose outgrowths on arthrodized frontalmost appendage [AC76]
0. Absent
1. Present

[82] Type of inner (ventral) spinose outgrowths on frontalmost appendage [AC77]
0. Sub-equal length or tapering gradually along entire margin
1. Elongate mid-margin

[83] Secondary spines on inner (ventral) spinose outgrowths of frontalmost appendage [AC78]
0. Absent
1. Present

[84] Outer (dorsal) spinose outgrowths on arthrodized frontalmost appendage [AC79]
0. Absent
1. Present

[85] Outer (dorsal) spinose outgrowth on arthrodized frontalmost appendage with elongate terminal spine [AC80]
0. Absent
1. Present

OTHER CEPHALIC LIMBS

[86] All cephalic endopods posterior to second pair well-developed [AC81]
0. Absent
1. Present

[87] Endopod of second appendage pair [AC82]
0. Developed
1. Reduced

[88] Endopod of third appendage pair [AC83]
0. Developed
1. Reduced

[89] Endopod of fourth appendage pair [AC84]
0. Developed
1. Reduced

[90] Exopod of fourth appendage pair [AC112]*
0. Developed
1. Reduced

[91] Endopod of fifth appendage pair [AC85]
0. Developed
1. Reduced

[92] Some cephalic endopods are walking limbs [AC86]
0. Absent
1. Present

[93] Repeated appendage morphology in tagma I [AC87]
0. Absent

1. Present

[94] Dichotomy in appendage morphology between tagma I and tagma II [AC88]
0. Absent
1. Present

[95] Proximo-distal differentiation of endopod podomeres in head (tagma I) [AC89]
0. Absent
1. Present

[96] Podomere number in head (tagma I) [AC90]
0. 7
1. <7
2. >7

[97] Post-antennular appendage expressed [AC91]
0. Absent
1. Present

[98] Post-antennular appendage differentiated [AC92]
0. Absent
1. Present

[99] Chelate or sub-chelate termination of post-antennular appendage [AC93]*
0. Absent
1. Present

[100] Ramification of post-antennular appendage [AC95]
0. Uniramous
1. Biramous

[101] Developed endites on endopod of post-antennular appendage [AC96]
0. Absent
1. Present

[102] Endopod of post-antennular appendage annulate or flagellate [AC97]
0. Absent
1. Present

[103] Podomere number of endopod of post-antennular appendage [AC98]
0. <7
1. 7

[104] Coxa on post-antennular appendage [AC99]
0. Absent
1. Present

[105] Exopod of post-antennular appendage, type [AC100]
0. Stenopodous
1. Annulate
2. Rod-shaped
3. Paddle
4. Tripartie

[106] Exopods on cephalic appendages excluding two anteriormost pairs [AC101]
0. Absent

1. Present

[107] Exopod of cephalic appendages excluding two anteriormost pairs, type [AC102]
0. Stenopodous
1. Annulate
2. Rodiform
3. Paddle
4. Tripartite

[108] Multisetose, rounded tip on cephalic exopods [AC103]
0. Absent
1. Present

[109] Partial detachment of exopods from main limb branch in head tagma*
0. Absent
1. Present

Remark: This character expresses the peculiar condition of habeliidans, *Offacolus* and *Dibasterium*, in which the cephalic exopods preserve as partially dissociated from their main biramous branch (Aria and Caron 2017). The exact attachment remains unknown.

[110] Enditic outgrowths on cephalic endopods excluding two anteriormost pairs [AC104]
0. Absent
1. Present

[111] Endopod of third cephalic appendage chelate or subchelate [AC105]
0. Absent
1. Present

[112] Third cephalic appendage with a well-developed gnathobase [AC106]
0. Absent
1. Present

[113] Third cephalic appendage a mandible [AC107]
0. Absent
1. Present

[114] Mandible with three-segmented endopod, appressed on the ventral side of the head, curving inward [AC94]*
0. Absent
1. Present

Remark: This characterizes the endopod of the fuxianhuiid mandible.

[115] Mandibular palp [AC107]
0. Non-developed
1. Developed

[116] Telognathic mandible [AC108]
0. Absent
1. Present

[117] Mandibular gnathal edge [AC109]
0. Consisting of molar and incisor process
1. Only ellipsoid pars molaris present
2. Row of parallel teeth

3. Shovel with terminal teeth
4. Group of paired teeth and hair pad

[118] Mandibular lamellate combs [AC110]
0. Absent
1. Present

[119] Hypopharynx [AC111]
0. Absent
1. Present

[120] Modified endopod/palp on fourth cephalic appendage [AC113]
0. Absent
1. Present

Remark: This character implies the modification of the appendage basis as a mouthpart and the reduction of the endopod of the fourth appendage pair (char. 89), whereby the complete reduction of the endopod is coded "0".

[121] Modified endopod/palp on fourth cephalic appendage, type [AC114]*
0. Reduced, vestigial, undeveloped
1. Well developed

[122] Post-mandibular plate formed by the fusion of the maxilla and the intermaxillary sternum [AC115]
0. Absent
1. Present

[123] Cephalic appendages 4 and 5 ending with chelate termination [AC116]
0. Absent
1. Present

[124] Fifth cephalic appendage [AC117]
0. Integrated to gnathal plate (labium)
1. Reduced, enditic

[125] Fifth cephalic appendage vestigial [AC118]
0. Absent
1. Present

[126] Fifth cephalic appendage with developed palp [AC119]
0. Absent
1. Present

Remark: Same requirements for coding as for char. 120, but with respect to fifth cephalic pair.

[127] Internalization of mouthparts [AC120]
0. Absent
1. Present

[128] Oral cone [AC121]
0. Absent
1. Present

[129] Atrium oris [AC122]
0. Absent
1. Present

MOUTH AND STOMODAEAL AREA

[130] Mouth opening anteriorly (as opposed to ventrally or dorso-ventrally) [AC123]
0. Absent
1. Present

[131] Type of circumoral structures [AC124]
0. Toothed lips
1. Lamellae
2. Plates
3. Hypostome-labrum complex

[132] Circumoral structures sclerotized [AC125]
0. Absent
1. Present

[133] Proboscis [AC126]
0. Absent
1. Present

ALIMENTARY TRACT AND OTHER INTERNAL CHARACTERS

[134] Stomach [AC129]
0. Absent
1. Present

[135] Stomach in a frontal position [AC131]
0. Absent
1. Present

[136] Stomach—additional pouch [AC132]
0. Absent
1. Present

[137] Secondary organs connected to the central digestive duct [AC132]
0. Absent
1. Present

[138] Secondary digestive organs serially repeated along the post-cephalic portion of the gut [AC133]
0. Absent
1. Present

[139] Shape of post-cephalic secondary digestive structures [AC134]
0. Reniform
1. Bulgy triangles
2. Caeca

[140] Striations on post-cephalic secondary digestive structures [AC135]
0. Absent
1. Present

[141] Branching of post-cephalic secondary digestive structures [AC136]
0. Absent
1. Present

[142] Differentiation of cephalic secondary digestive structures (compared to trunk) [AC137]
0. Absent
1. Present

[143] Ramification of cephalic secondary digestive structures [AC138]
0. Absent
1. Present

[144] Branching of cephalic secondary digestive structures [AC139]
0. Absent
1. Present

[145] Peritrophic membrane [AC140]
0. Absent
1. Present

[146] Metameric ganglia on nerve cord [AC141]
0. Absent
1. Present

[147] Metanephridia with sacculus containing podocytes [AC142]
0. Absent
1. Present

[148] Segmental invaginations of neuroectoderm giving rise to ventral organs [AC143]
0. Absent
1. Present

TRUNK

[149] Thorax [AC144]
0. Absent
1. Present

[150] Number of thoracic somites [AC145]
0. 11
1. 5
2. 8
3. 3

[151] Abdomen [AC146]
0. Absent
1. Present

[152] Number of core trunk segments [AC147]
0. >14
1. 12-14
2. 9
3. 7-8
4. <7

[153] Seventh appendage integrated into the prosoma [AC148]*
0. Absent
1. Present

Remark: This character only applies to Chelicerata and their stem groups (see also char. 32).

[154] Tergite of eighth somite (counting the ocular somite as the first) drastically reduced as a "microtergite"*
0. Absent
1. Present

Remark: See Dunlop and Lamsdell (2016) for a review of this character across chelicerates.

[155] Post-cephalic appendages covered by sclerotic plates (opercula)*
0. Absent
1. Present

Remark: Arguably the strongest apomorphy of Euchelicerata (Dunlop and Lamsdell 2016; Aria and Caron in press).

[156] Multisegmentation [AC149]
0. Absent
1. Present

[157] Tergo-sternal decoupling [AC150]
0. Absent
1. Present

[158] Tergo-sternal decoupling, type [AC151]
0. Polypody
1. Polypody and "polysternity"
2. "Polytergity" (autapomorphy of symphylan myriapods)

[159] Pleurae [AC152]
0. Reduced or fused
1. Developed

[160] Tergo-pleural rings [AC153]*
0. Absent
1. Present

[161] Pleural orientation [AC154]
0. Horizontal
1. Around body

[162] Pleural length [AC155]
0. Short, i.e. equal or inferior to body diameter
1. Long, i.e. exceeding body diameter

[163] Articulating ridge [AC156]
0. Absent
1. Present

[164] Articulating ridge, type [AC150]
0. Single
1. Antero-posterior

[165] Transverse stipital muscle [AC150]
0. Absent
1. Present

TRUNK APPENDAGES AND GENERAL APPENDICULAR CHARACTERS

[166] Proximo-distal differentiation of endopod podomeres in tagma II [AC159]
0. Absent
1. Present

[167] Podomere number in tagma II [AC160]
0. 7
1. <7
2. >7

[168] Maxillipeds [AC161]
0. Absent
1. Present

[169] Tergites of maxilliped segments fused to head shield [AC162]
0. Absent
1. Present

[170] Single main maxilliped [AC163]
0. Absent
1. Present

[171] Slit sensilla [AC164]
0. Absent
1. Present

[172] Basis (basipod) [AC165]
0. Absent
1. Present

[173] Basipod formed of at least two elements [AC166]
0. Absent
1. Present

[174] Basipod multi-segmented [AC167]
0. Absent
1. Present

[175] Multiple endites on basipod [AC168]
0. Absent
1. Present

[176] Proximal endite [AC169]
0. Absent
1. Present

[177] Coxa as entire pre-basal podomere [AC170]
0. Absent
1. Present

[178] Precoxa as whole pre-coxal podomere [AC171]
0. Absent
1. Present

[179] Pleurites formed by several sclerotic elements surrounding limb insertion [AC172]

0. Absent
1. Present

[180] Arrangement of pleurites [AC173]
0. Outer/proximal and distal/inner sets
1. Multiple sclerotic pieces

[181] Gnathobases [AC174]
0. Absent
1. Present

[182] One or more gnathobase(s) reduced in tagma I [AC175]
0. Absent
1. Present

[183] Secondary appendicular outgrowths on trunk [AC176]
0. Absent
1. Present

[184] Secondary appendicular outgrowths on trunk, type [AC177]
0. Lobopodous
1. Sclerotized

[185] Proximal lamellae [AC178]
0. Absent
1. Present

[186] Proximal lamellae internalized [AC179]
0. Absent
1. Present

[187] Trunk endopod reduced posterior to head tagma [AC180]
0. Absent
1. Present

[188] Limb arthrodization in trunk [AC181]
0. Absent
1. Present

[189] Trunk exopod posterior to head tagma, type [AC182]*
0. Paddle/lobe
1. Rodiform
2. Annulate
3. Reduced
4. Three-segmented
5. Phyllopodous

[190] Endopod strongly developed in thorax (or anterior trunk if thorax undifferentiated) [AC183]
0. Absent
1. Present

[191] Phyllopodous-type limbs anywhere on body [AC184]
0. Absent
1. Present

[192] Terminal endopods stenopodous [AC185]

0. Absent
1. Present

[193] Identical morphology of endopod and exopod rami on pleopods/post-thorax [AC186]
0. Absent
1. Present

[194] Annulation of at least one pair of exopods [AC187]
0. Absent
1. Present

[195] Subdivision of at least one pair of exopods [AC188]*
0. Absent
1. Present

[196] Subdivision of at least one pair of exopods, type [AC188]*
0. Bipartite
1. Tripartite

[197] Attachment segment in lobate exopod [AC189]
0. Absent
1. Present

[198] Epipod [AC191]
0. Absent
1. Present

[199] Endite as a latero-distal projection on endopod podomeres [AC192]
0. Absent
1. Present

[200] Pusher legs with paddle tips [AC193]
0. Absent
1. Present

[201] Developed endites on endopod podomeres in trunk (tagma II and III) [AC194]
0. Absent
1. Present

[202] Paired spines on endopod podomere [AC195]
0. Absent
1. Present

[203] Short spines on endopod podomere [AC196]
0. Absent
1. Present

[204] Multiple setae on endopod podomere [AC197]
0. Absent
1. Present

[205] Paired elongate spines distally on endopod [AC198]
0. Absent
1. Present

[206] Limb tip [AC199]

0. Pad
1. Juxtaposed claws
2. Trident of claws
3. Chelate or sub-chelate
4. Double claw
5. Multiple spines
6. Single claw

POSTERIOR TERMINATION

[207] Sclerotization of termination [AC200]
0. Absent
1. Present

[208] Telson developed [AC201]
0. Absent
1. Present

[209] Telson type [AC202]
0. Spine
1. Plate
2. Spatula

[210] Anus location [AC203]
0. Terminal, last segment
1. Base of telson

[211] Caudal rami [AC204]
0. Absent
1. Present

[212] Additional caudal processes [AC205]
0. Absent
1. Present

[213] Furca [AC206]
0. Absent
1. Present

[214] Uropods sensu stricto [AC207]
0. Absent
1. Present

[215] Caudal rami, type [AC208]
0. Spinose
1. Rounded
2. Filamentous
3. Annulate

[216] Pygidium [AC209]
0. Absent
1. Present

[217] Type of pygidial fusion [AC210]
0. Partial
1. Complete

[218] Axial elevation of pygidium [AC211]
0. Absent
1. Present

[219] Pygidial ornamentation [AC212]
0. Smooth
1. Spinose

REFERENCES

ARIA, C. and CARON, J.-B. 2017a. Burgess Shale fossils illustrate the origin of the mandibulate body plan. *Nature*, **545**, 89-92.

--- 2017b. Mandibulate convergence in an armoured Cambrian stem chelicerate. *BMC Evolutionary Biology*, **17**, 261.

DUNLOP, J. A. 1997. The origins of tetrapulmonate book lungs and their significance for chelicerate phylogeny. *In* SELDEN, P. A. (ed.) *Proceedings of the 17th European Colloquium of Arachnology*. Edinburgh.

DUNLOP, J. A. and LAMSDELL, J. C. 2016. Segmentation and tagmosis in Chelicerata. *Arthropod Structure & Development*, **46**, 395-418.

HAUG, C., SALLAM, W. S., MAAS, A., WALOSZEK, D., KUTSCHERA, V. and HAUG, J. T. 2012. Tagmatization in Stomatopoda - reconsidering functional units of modern-day mantis shrimps (Verunipeltata, Hoplocarida) and implications for the interpretation of fossils. *Frontiers in Zoology*, **9**.

OLESEN, J., HAUG, J. T., MAAS, A. and WALOSZEK, D. 2011. External morphology of *Lightiella monniotae* (Crustacea, Cephalocarida) in the light of Cambrian 'Orsten' crustaceans. *Arthropod Structure & Development*, **40**, 449-478.

OLESEN, J. and WALOSSEK, D. 2000. Limb ontogeny and trunk segmentation in *Nebalia species* (Crustacea, Malacostraca, Leptostraca). *Zoomorphology*, **120**, 47-64.

SCHOLTZ, G. and EDGECOMBE, G. D. 2006. The evolution of arthropod heads: reconciling morphological, developmental and palaeontological evidence. *Development Genes and Evolution*, **216**, 395-415.

SCHRAM, F. R. 1986. *Crustacea*. Oxford University Press, 606 pp.

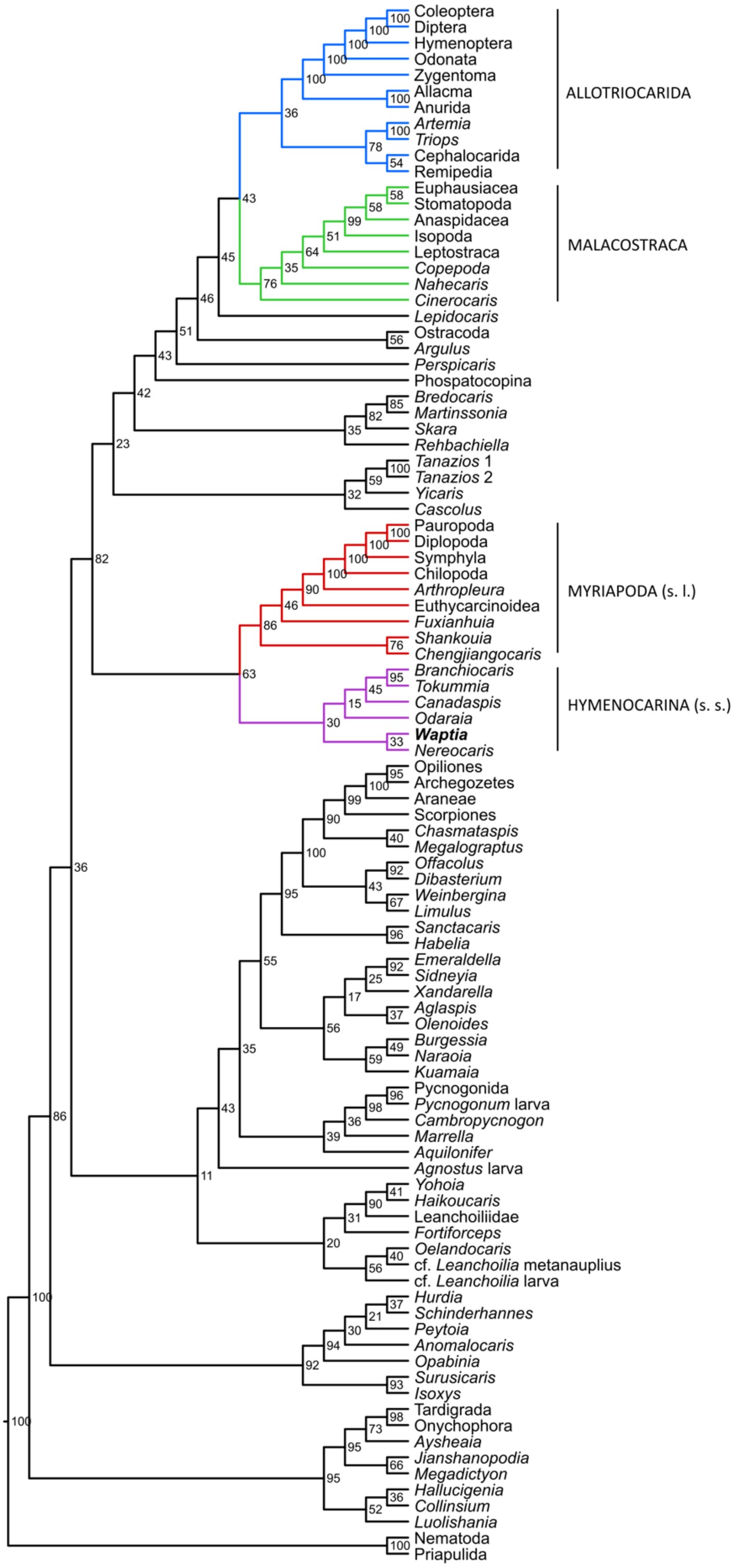

Electronic supplementary material S22. Maximum clade credibility trees of time-calibrated Bayesian analyses of panarthropod relationships (Mkv+Γ model, 219 characters). Consensus topology resulting from a dataset of 97 larval and adult taxa. Numbers on the right of nodes are posterior probabilities.
